# Chemometric Combination of Ultrahigh Resolving Mass Spectrometry and Nuclear Magnetic Resonance Spectroscopy for a Structural Characterization of Lignin Compounds

**DOI:** 10.1021/acsomega.3c06222

**Published:** 2023-12-19

**Authors:** Lara Dütsch, Klara Sander, Erica Brendler, Martina Bremer, Steffen Fischer, Carla Vogt, Jan Zuber

**Affiliations:** † Institute of Analytical Chemistry, TU Bergakademie Freiberg, Leipziger Strasse 29, Freiberg 09599, Germany; ‡ Institute of Plant and Wood Chemistry, TU Dresden, Pienner Strasse 19, Tharandt 01737, Germany

## Abstract

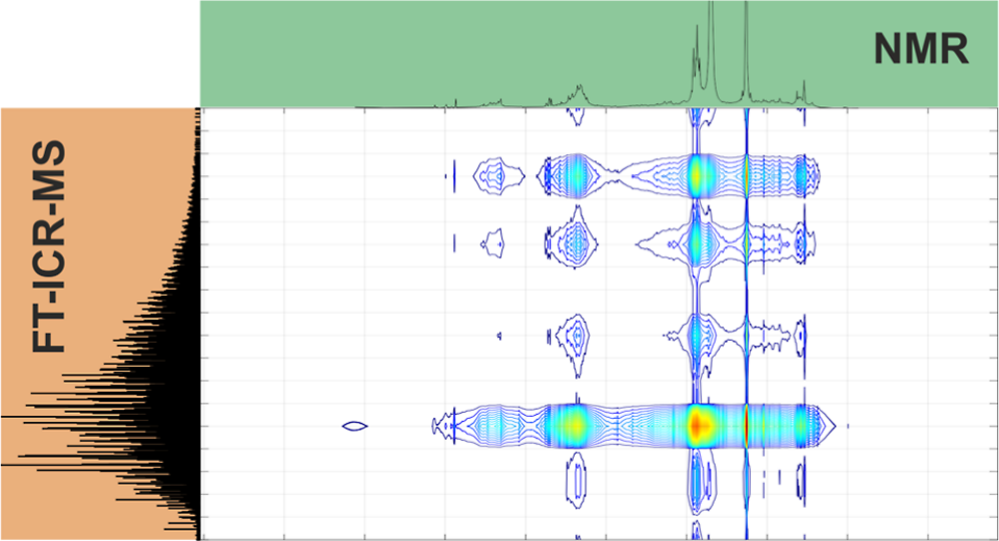

In recent years, the potential of lignins as a resource for material-based applications has been highlighted in many scientific and nonscientific publications. But still, to date, a lack of detailed structural knowledge about this ultracomplex biopolymer undermines its great potential. The chemical complexity of lignin demands a combination of different, powerful analytical methods, in order to obtain these necessary information. In this paper, we demonstrate a multispectroscopic approach using liquid-state and solid-state Fourier transform ion cyclotron resonance mass spectrometry (FT-ICR-MS) and nuclear magnetic resonance (NMR) spectroscopy to characterize a fractionated LignoBoost lignin. Individual FT-ICR-MS, tandem MS, and NMR results helped to determine relevant information about the different lignin fractions, such as molecular weight distributions, oligomer sizes, linkage types, and presence of specific functional groups. In addition, a hetero spectroscopic correlation approach was applied to chemometrically combine MS, MS/MS, and NMR data sets. From these correlation analyses, it became obvious that a combination of tandem MS and NMR data sets gives the opportunity to comprehensively study and describe the general structure of complex biopolymer samples. Compound-specific structural information are obtainable, if this correlation approach is extended to 1D-MS and NMR data, as specific functional groups or linkages are verifiable for a defined molecular formula. This enables structural characterization of individual lignin compounds without the necessity for tandem MS experiments. Hence, these correlation results significantly improve the depth of information of each individual analysis and will hopefully help to structurally elucidate entire lignin structures in the near future.

## 1 Introduction

Lignin is the second most abundant biological resource on the earth, after cellulose, with an amount of 20–30% in the dry mass of woody plants.^1,2^ Especially during lignocellulosic refinery and pulp production, lignin is accumulated in considerable amounts (>80 million tons per year).^2^ The overall structure of this complex, phenolic, three-dimensional, and amorphous macromolecule is not entirely understood to this date. Hence, material-based utilizations of lignin are currently limited to a small application range (preparation of humus substitutes, depot fertilizers,^3^ or hydrogels^4^) and most of the lignin is still used for power generation. In order to enhance the application range of lignins, a more detailed understanding of the lignin structure is necessary. This information is only derivable if high-resolving analytical techniques are utilized.

Usually, lignin characterization is performed by using size-exclusion chromatography (SEC), infrared (IR) and Raman spectroscopy, or pyrolysis-gas chromatography-mass spectrometry (Py-GC/MS).^5−12^ These analytical methods are, in most cases, time-consuming and only able to assist in the characterization of a specific part of the lignin sample, such as the molecular weight (SEC), monolignol content (Py-GC/MS), or characteristics of specific structural subunits (IR and Raman). More comprehensive structural information about the lignin samples are obtainable, if more powerful analytical techniques such as nuclear magnetic resonance (NMR) spectroscopy and high-resolution MS (HR-MS) are utilized.

NMR spectroscopy can be helpful to determine structural information about linkage types or the presence of functional groups in lignin compounds. By using one-dimensional (1D) liquid-state NMR methods, a general overview about the structural diversity can be provided.^13^ Quantitative ^13^C NMR measurements allow for an evaluation of the structural elements. This way, the number of methoxy groups per aromatic unit and therefore the ratio of the monolignols (S/G/H-ratio), as well as linkages between them, can be obtained.^14,15^ Additionally, two-dimensional (2D) approaches result in better resolved spectra and simplify the assignment of signals. Especially, heteronuclear single-quantum coherence (HSQC)-NMR approaches, where correlations between directly coupled protons and carbons can be detected, allow to distinguish linkages as well as the aromatic carbon atoms of the different monolignols.^10,14,16−21^ By using quantitative ^1^H,^13^C-HSQC-NMR experiments like HSQC_0_,^9,22^ or quick and quantitative (QQ)-HSQC,^9,23,24^ the proportions of the lignin substructures can be determined. In addition to liquid-state NMR, solid-state NMR spectra in combination with magic angle spinning (MAS) can be used to gain information about insoluble components in a lignin sample. The ^1^H,^13^C-heteronuclear correlation (HETCOR)-NMR spectroscopy helps to verify correlations between carbons and attached protons. By increasing the contact time, additional correlations between spatially adjacent nuclei with a maximum distance of 6 Å can be revealed.^25^

In contrast, HR-MS gives the opportunity to study the complexity of lignin compounds, with regard to the molecular weight, oligomer size, linkage types, and the presence of functionalities in lignin molecules from different sources.^26−28^ Various research groups were able to demonstrate that HR-MS in combination with electrospray ionization (ESI)^26,29−34^ atmospheric pressure chemical ionization (APCI)^35^ and photo ionization (APPI)^36^ or matrix-assisted laser desorption/ionization (MALDI)^37−42^ can be utilized to study, for instance, Kraft lignins, organosolv lignins, or electro- and photochemically degraded lignins. In addition, tandem MS analyses are helpful to obtain detailed structural information about different lignin molecules.^27,29,34,35,43−47^ Comprehensive investigations of lignins and other complex mixtures are possible by utilizing the most powerful MS technique, Fourier transform ion cyclotron resonance MS (FT-ICR-MS).^48,49^ For example, the potential of FT-ICR-MS for lignin analyses was recently demonstrated by characterizing soluble and insoluble compounds from different Kraft and LignoBoost lignins (LBL)^50^ with the help of ESI and graphite-assisted laser desorption/ionization (GALDI).^51−54^

Despite the advancements in the field of lignin characterization by NMR and HR-MS in the last years, to date, a holistic characterization of lignin and its complex structure seems only possible if results from different analytical techniques are combined. An efficient conjunction of data sets from different methods to enhance the information content of each individual technique is possible by using the chemometric method of hetero spectroscopic correlation analysis.^55−57^ For this method, an original sample is processed by applying an external perturbation (temperature, pressure, etc.), which leads to a set of different samples that can be analyzed by different analytical techniques. The spectral variation of these different data sets is then used to investigate the complex cross correlation, which is usually visualized by a 2D correlation spectrum.^57^ For instance, a hetero spectroscopic correlation of NMR and MS or MS/MS data sets was already utilized by other research groups to structurally identify tomato metabolites^58^ or to investigate typical biomarkers in the urine of rats.^59^

The aim of this paper is to demonstrate how a hetero spectroscopic correlation approach can help to comprehensively study the structure of lignin compounds. For this purpose, a set of fractionated LignoBoost lignin (LBL) samples was analyzed by means of liquid-state ^1^H NMR, quantitative ^13^C NMR, and ^1^H,^13^C-HSQC-NMR spectroscopy, as well as solid-state ^13^C NMR and ^1^H,^13^C-HETCOR-NMR spectroscopy. In addition, the fractions were characterized by ESI-FT-ICR-MS and solid GALDI-FT-ICR-MS. Selected molecular ions were also structurally investigated by MS/MS with the help of collision-induced dissociation (CID). In a first step, the result from the individual NMR, FT-ICR-MS, and MS/MS analyses helped to characterize the different LBL fractions in general. From these analyses, we were able to derive relevant information about the different lignin fractions, such as molecular weight distributions, oligomer sizes, linkage types, and presence of specific functional groups. In a second step, data sets from FT-ICR-MS or MS/MS analyses of the different samples were correlated with results from liquid-state ^1^H NMR and solid-state ^13^C NMR analyses. From these correlation analyses, it became obvious that a combination of tandem MS and NMR data sets gives the opportunity to comprehensively study the overall structure of lignins and other complex biopolymer samples. Compound-specific structural information are obtainable if this correlation approach is applied to 1D-MS and NMR data. From these hetero spectroscopic correlation results, specific functional groups or linkages can be verified for single molecules without the need for tandem MS experiments. The applied correlation approach improves the depth of information of each individual analysis and will hopefully help to structurally elucidate structures of lignins from different sources and pulping or modification processes in the near future.

## 2 Experimental Section

### 2.1 Fractionated LBL Samples

The original LBL sample was prepared from coniferous wood by Mercer Rosenthal GmbH. Shredded wood was subjected to a Kraft process, where a biomass sample is treated with sodium hydroxide (NaOH) and sodium sulfide (Na_2_S) for several hours at temperatures between 155 and 175 °C. In this process, cellulose remains as a solid residue and lignin is dissolved in the black liquor.^2^ This black liquor is processed with CO_2_ afterward, before sulfuric acid (H_2_SO_4_, 0.1 M) is utilized to neutralize the solution and to reduce the ash and sugar content of the lignin sample.

The fractionation was performed with the help of a water–acetone solvent mixture, which was used to dilute both polar and less-polar lignin compounds. With an increasing fraction number, the amount of acetone increased by 10% (v). The first fraction was generated by using a solvent mixture consisting of 90% H_2_O (v/v) and 10% acetone (v/v), whereas the last fraction was produced with a 30% H_2_O (v/v) and 70% acetone (v/v) solvent mixture. In total, seven different LBL fractions (F1–F7) were produced from this fractionation as F7 represents the fraction with all insoluble residues from F6. F2 could not be analyzed by NMR spectroscopy and FT-ICR-MS as the amount of the fractionated sample was too low to realize an analysis with both analytical techniques. This can be attributed to the high chemical similarity of the compounds, which are dilutable with the first two solvent mixtures. Thus, only F1 and F3–F7 were subjected to the different analytical methods and characterized for this research work.

### 2.2 NMR Spectroscopy

Solution NMR analyses were performed with a BRUKER Avance Neo 700 MHz spectrometer (Bruker Biospin), equipped with a triple-resonance probe (TXI) for ^1^H (spectrometer frequency 700.13 MHz) and ^1^H,^13^C-HSQC measurements. Approximately, 150 mg of the sample was dissolved in 0.7 mL (F1) or 0.75 mL (F3–F7) of deuterated dimethyl sulfoxide (DMSO-*d*_6_, deutero 99.5 at. % D). Due to broad signals in the ^1^H-spectrum of F1, this measurement was repeated in a diluted solution, consisting of 10–20 μL of the stock solution in 0.65 mL of DMSO-*d*_6_. All samples were stored under a nitrogen atmosphere in 5 mm Young tubes for better stability and to prevent oxidation processes. 2D-HSQC-spectra were recorded using a repetition time of 2.0 s, 400 scans, 256 increments, and 50% nonuniform sampling (NUS) in the indirect dimension.

For quantitative ^13^C NMR spectra (inverse gated decoupling, spectrometer frequency 176.09 MHz), a broad band observe probe was used. To achieve quantitative spectra, the relaxation of all of the nuclei must be complete. ^13^C nuclei have a long T_1_ relaxation time (spin-lattice relaxation time), which leads to long experimental times. To reduce T_1_, paramagnetic relaxation agents like chromium(III)acetylacetonate [Cr(acac)_3_] can be used. The unpaired electron in Cr(acac)_3_ leads to a faster relaxation of the ^13^C nuclei due to electron–nucleus dipolar–dipolar interactions.^60,61^ F1 has a high content of paramagnetic impurities [Mn^2+^, confirmed by electron spin resonance spectroscopy (ESR),^62^ see Section S2 in the Supporting Information of this paper]. The number of paramagnetic centers in F3–F7 was too low for a reasonable fast relaxation. Hence, new samples with 0.01 M Cr(acac)_3_ (Merck Schuchardt, for synthesis, purity ≥98%) in DMSO-*d*_6_ were prepared, allowing for an experiment repetition time of 2.0 s. Up to 30,000 scans were recorded.

^13^C solid-state NMR measurements were carried out at 100.6 MHz on a 400 MHz Bruker AVIII HD WB spectrometer, equipped with a CP/MAS probe using 4 mm ZrO_2_ rotors at 10 kHz spinning speed. A contact time of 7 ms, optimized for sample F3, with an 80% ramp on the ^1^H channel was applied for CP. LBL F3 was used because prior analyses indicated the presence of lignin compounds with lower molecular masses and no paramagnetic substances in this fraction in comparison to F1 (see Supporting Information, Section S2). Experiment recycle delay was 3.0 s (see S6 and S7 in the Supporting Information), acquisition times from 15 to 30 ms and a tppm15 decoupling sequence were applied during acquisition, and 4k scans were accumulated. The chemical shift values were referenced externally using the CH_2_ group signal of adamantane (38.5 ppm with respect to TMS = 0 ppm). ^1^H,^13^C-HETCOR-NMR spectra were recorded using contact times of 200 μs as well as 3.0 ms and 100 F1 increments with 400 scans each.

The acquisition and processing of all spectra were performed using *Topspin* (version 4.0.6 and 3.6.2) by Bruker Biospin. The data were further analyzed and visualized in *MestReNova* (version 14.1.0-24037).

### 2.3 Mass Spectrometry

All LBL fractions were analyzed by means of ESI- and GALDI-FT-ICR-MS. For the ESI-MS analyses, solid lignin samples were weighed in amber glass vials and then diluted with DMSO, analogous to the liquid-state NMR experiments, to a concentration of 1 g/L. These stock solutions were further diluted to a final concentration of 100 mg/L with a solvent mixture consisting of 80% (v/v) MeOH, 19% (v/v) dichloromethane (DCM), 0.9% (v/v) H_2_O, and 0.1% (v/v) triethylamine (NEt_3_). Analyte solutions were transferred to a 1 mL gastight syringe and pumped into the ESI source at a flow rate of 10 μL/min.

For the solid GALDI-FT-ICR-MS analyses, 30 mg of a 1:5 (m/m) mixture of lignin and graphite was weighed in amber glass vials. 200 μL of tetrahydrofuran and 15 μL of paraffin oil were added to this solid mixture. Afterward, this suspension was sonicated for 10 min and 1.2 μL of the suspension was applied spot-wise on a MALDI steel target and air-dried.

All utilized solvents were of HPLC grade quality and were purchased from Merck Chemicals, Carl Roth, Chemsolute, or VWR Chemicals. Graphite powder was purchased from Mikro to Nano (purity ≥99.9%, particle size 5 μm).

MS experiments were conducted on a 15 T solariX FT-ICR-MS instrument from Bruker Daltonics, equipped with an ESI source as well as a MALDI source with a Smart Beam II laser (frequency tripled Nd/YAG laser, λ = 355 nm, pulse duration 3 ns, pulse energy 500 μJ, peak power 170 kW, and average power 1.5 W). For the ESI-FT-ICR-MS analyses, the capillary voltage was set to +2800 V, an end-plate-offset of −500 V, a nebulizer gas pressure of 1.0 bar, a dry gas flow of 4.0 L/min, and a dry gas temperature of 300 °C were utilized. GALDI analyses were performed by using the ultralarge laser focus (laser spot size of approximately 250 μm), a laser shot number of 20, a plate offset of −60 V, a deflector plate voltage of −180 V, and a laser frequency of 500 Hz. All analyses were conducted in the negative ion mode using a scan range from 153.52 to 5000.00 Da and a Q1 mass of 170 Da. Resulting data sets had a size of 8 M, while all fractions were analyzed using 256 scans. The resolving power was *R* = 800,000 at *m*/*z* = 400 Da for all analyses.

Tandem MS analyses were performed by isolating 60 representative ions in an *m*/*z* range from 290 to 820 Da with the quadrupole unit of the FT-ICR-MS. Fragmentation reactions were induced with the help of CID by applying individual collision energies (CEs) from 0 to 40 eV and argon as collision gas. 64 scans were accumulated for these experiments, and all ions in an *m*/*z* range from 153.52 to 2000.00 Da were detected.

Data calibration and evaluation of the FT-ICR-MS and tandem MS experiments were conducted according to previously published procedures.^50,63^

### 2.4 Correlation Spectroscopy

The combination of MS, tandem MS, and NMR data sets was performed by applying a 2D correlation spectroscopic approach.^57^ For this chemometric combination, the NMR and mass spectra were exported as text files and imported into *MATLAB* R2022b using in-house scripts. Hence, for each analyzed LBL fraction, data sets from the MS and NMR analyses could be utilized. From these data sets, the correlation coefficient Φ was calculated, according to eq 1, using the self-written *MATLAB* programs.
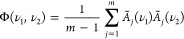
1

According to eq 1, *m* represents the overall number of analyzed samples (*m* = 6), *A* the vector of signals (e.g., intensities or integrals), ν the data dimension for each analytical technique, and *j* the current sample number. As a result, an individual correlation matrix could be calculated for the analyses of each fraction, based on the individual MS, tandem MS, and NMR results. In a last data evaluation step, these correlation matrices were summed up and plotted as heatmaps to visualize general trends between MS or tandem MS and NMR results. Thus, the perturbation, which is required to generate samples and data sets that are applicable for hetero spectroscopic correlation analysis, was the fractionation of the LBL sample into the seven fractions from which six were analyzed.

With this approach, it is possible to chemometrically combine unfiltered spectral data (raw spectra) with each other, as well as other spectral features, such as intensity values for selected typical lignin fragment ions (tandem MS) or quantitative amounts of lignin substructures (^13^C NMR). The information depth of each of these different evaluations will be discussed in detail in the upcoming section of this paper.

## 3 Results and Discussion

### 3.1 Liquid-State NMR Spectroscopy

**Figure 1:**
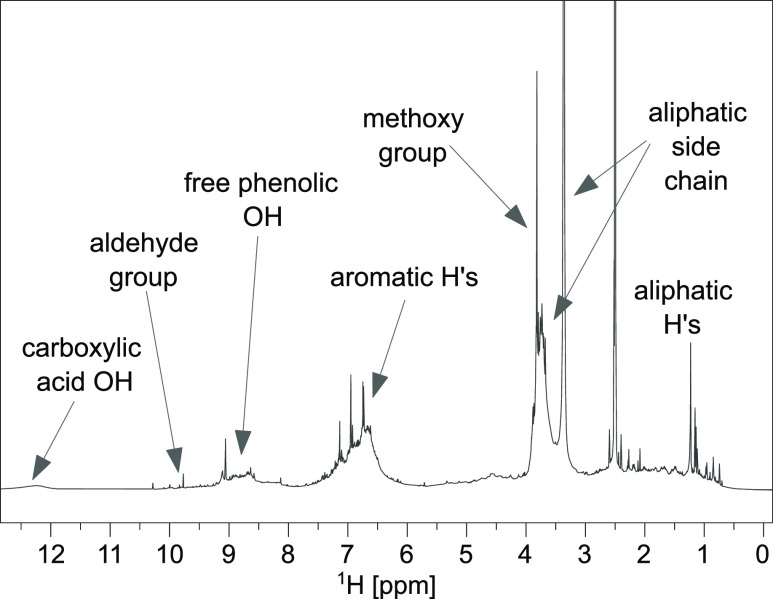
^1^H NMR spectrum of F3 in DMSO-*d*_6_. The solvent (2.5 ppm), aliphatic side chain (3.4 ppm), and methoxy group (3.8 ppm) signals were cut off.

1D- and 2D-NMR spectra were recorded to analyze the monolignol composition as well as the different linkages between them. Due to its high sensitivity, easy preparation, and short experiment time, ^1^H NMR spectroscopy is suitable to obtain a general overview about structural elements in the samples. Figure 1 shows the ^1^H NMR spectrum of F3 because this sample showed better resolution in comparison to the other samples (also see S1). Additionally, in Table S1, the most characteristic spectral regions are summarized and assigned. Besides the signals for the aromatic unit (7.5–6 ppm) as well as the side chains (4–3.5 ppm), between 10.5 and 9.5 ppm, additional signals of aldehyde groups are visible. This indicates the presence of cinnamyl aldehydes. The broad signal at 12.4 ppm is caused by fast exchanging protons of carboxylic groups due to oxidized side chains. Below 2.7 ppm, signals of remaining aliphatic components, such as C–H groups bonded to aromatics or carbonyls (2.7–1.7 ppm) and aliphatic C–H-groups (below 1.7 ppm), are visible.^18^

By comparison of the ^1^H NMR spectra of all six fractions (see Figure S2), a few differences become visible. The original sample of F1 showed broad signals due to the high amount of paramagnetic ions, mainly Mn^2+^ (see the ESR spectroscopy evaluation in S2). To achieve better resolved signals, 10–20 μL of the sample was diluted in 0.6 mL of DMSO-*d*_6_. The signal of the alkoxy groups is one of the most intense signals in all six spectra (around 3.8 ppm). However, it is overlapping with the signals of the side chains between 4.0 and 3.3 ppm.^64^ With an increasing fraction number, the resolution decreases, which can be attributed to an increasing molecule size with a higher fraction number. This increasing molecule size results in limited motion and greater structural variation, which leads to broader signals.

**Figure 2:**
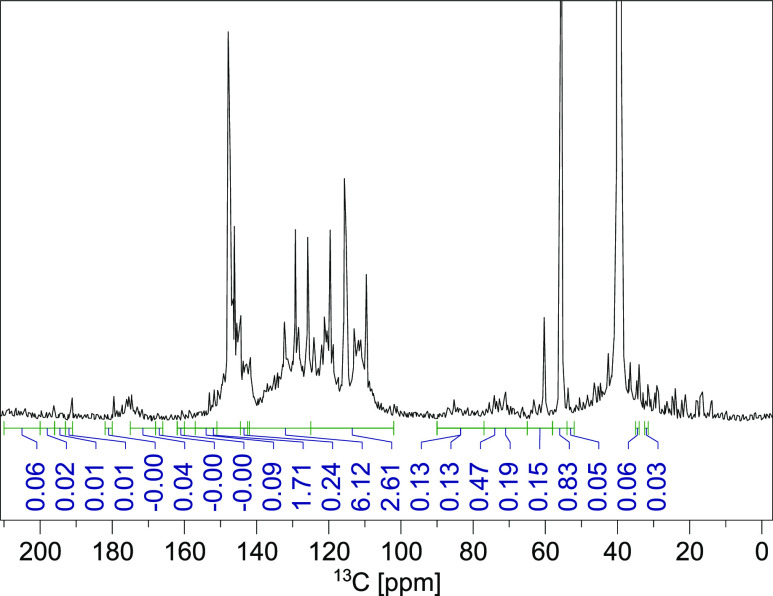
Quantitative ^13^C NMR spectrum of F3.

The composition of different lignins differs depending on their origin. Generally, lignin is composed of phenylpropanoid units that are linked by mainly ether bonds. There are three different phenylpropanoids: coumaryl, coniferyl, and sinapyl alcohol that form in the polymer *p*-hydroxyphenyl (H), guaiacyl (G), and syringyl (S) units, which vary only in the number of methoxy groups (zero to two). Hard woods are mainly composed of G units, soft woods additionally contain S units, and grasses and straws are formed of all three types.^2^ Hence, by identifying their S/G/H-ratio, information about the origin of a sample can be determined.^65^ The quantitative ^13^C NMR spectroscopy is useful for investigating the S/G/H-ratio as well as the amount of linkages and structural groups.^2^ Quantitative ^13^C NMR spectra were recorded for all six samples and integrated according to Capanema et al.^15^ by setting the integral between 162 and 102 ppm to 6.12 as a calibration standard. This area contains the signals of all six aromatic carbon atoms as well as the vinylic carbon of ferulate, *p*-coumarate, cinnamyl alcohol, and cinnamyl aldehyde.^15,20^ In Figure 2, the spectrum of F3 is shown. In S3 in the Supporting Information, the most important signals are assigned to their structural group (Figure S4 and Table S2). Here, the differences between the spectra are discussed additionally. The integrated regions, their assignments, and their integrals for the most important regions are summarized in Table 1, exemplary for F3. The results of the quantitative ^13^C NMR for all samples and integrated areas are presented in Section S4 in the Supporting Information.

**Table 1:** Assignments and Quantification (per C_9_ Unit) of the Most Important Structural Units, According to Capanema et al.^15^ (Complete Table in S4—Table S3)

no	δ [ppm]	assignment	per C_9_ unit
8	162–142	C_Ar–O_	1.78
12	142–125	C_Ar–C_	1.82
13	125–102	C_Ar–H_	2.52
14	90–58	C_Alk_—O–R	0.84
15	90–77	C_Alk_–O–Ar, C_α_–O–Alk	0.20
16	77–65	C_γ_–O–Alk, C–OH_sec_	0.35
17	65–58	C–OH_prim_	0.28
18	58–54	methoxy group	0.93

Basically, the integrals of C_Ar–O_, C_Ar–C_, and C_Ar–H_ (no. 8, 12, and 13, see Table 1) show important trends within the samples (also see Figures S6 and S7). All integral values of fractions F1 and F3–F7 are listed in Table S4. The C_Ar-H_ area is the most abundant structural feature in all samples (2.31 in F6 and up to 2.80 in F1). C_2_ and C_6_ in the aromatic units are generally unsubstituted. Depending on the amount of linkages derived from C_5_, it can be unsubstituted as well. According to the C_Ar–H_ integral in F1, only 20% of C_5_ is substituted, whereas in the fractions F5 and F6, more β-5′ linkages are present, indicating again an increasing *M*_w_ and cross-linking with an increasing fraction number. Therefore, the trend of C_Ar–C_ is inverse to C_Ar–H_. The number of C_Ar–O_ groups is similar in all analyzed fractions (1.71–1.93). Generally, in lignin extracted from coniferous woods, two O-containing substituents are present: the methoxy group at position 3 and the phenolic hydroxy group at position 4. However, the C_Ar–O_ integrals are lower than 2.0 and hence there is a modification, e.g., by the loss of one of these groups due to the Kraft process. The amount of methoxy groups can be quantified by the integral between 58 and 54 ppm (no. 18, see Table 1). F1 showed the highest content with 1.1 methoxy groups per Ar-unit, whereas the other fractions have 0.93–0.99 methoxy groups. This confirms the presence of mainly G units, which is characteristic for coniferous wood. By quantifying the aliphatic bonded hydroxy and ether groups (90–58 ppm), information about the linkage types can be gained. F1 has the highest content of C_Alk_–O–R groups (no. 14, see Table 1). Thus, in F1, the most β-O-4′ bonds between the monolignols are present. The content of β-O-4′ linkages is decreasing with a minimum at F4 and, additionally, the C_Ar-C_ signal is increasing, which indicates a higher amount of β-5′ linkages. Therefore, the content of β-O-4′ linkages is decreasing and more β-5′ linkages are present. In fraction F7, however, high contents of both linkage types exist. Hence, bigger lignin molecules can be supposed. Primary hydroxy groups C–OH_prim_ are mainly present at terminal C_γ_ groups of the side chain, for example, at β-O-4′ or β-5′ linkages. Statistically, in every second to fourth unit, a C–OH_prim_ group is present (no. 17, see Table 1). The content of secondary hydroxy groups C–OH_sec_ is higher (0.3 to 0.7), and they are mostly located at C_α_ or C_β_ carbon atoms.

**Figure 3:**
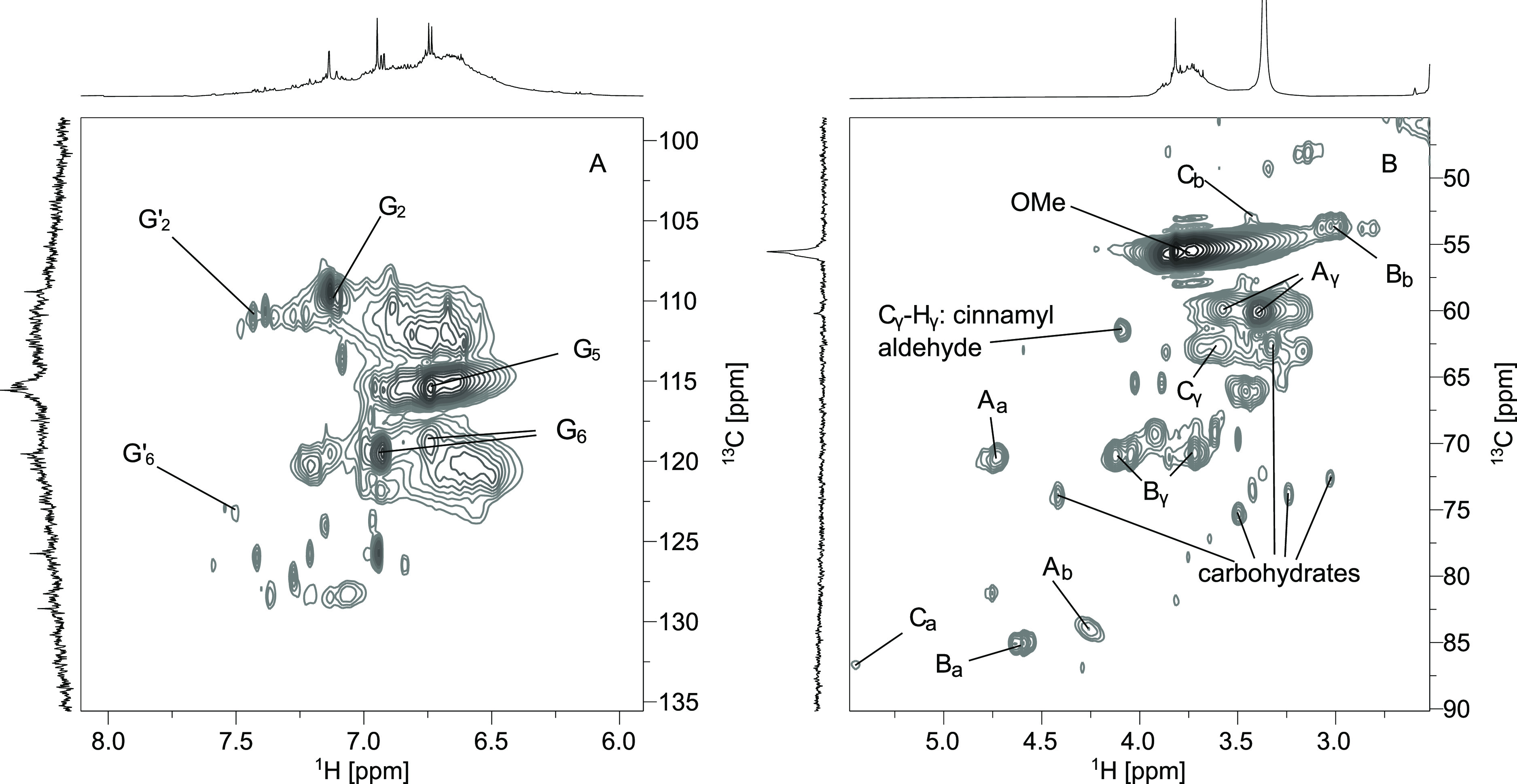
^1^H,^13^C-HSQC-NMR spectrum of F3 in DMSO-*d*_6_: (A) aromatic region and (B) aliphatic region.

The signals of the lignins are poorly resolved in the 1D-NMR spectra. By recording 2D-^1^H,^13^C-HSQC spectra, the resolution can be enhanced for the CH_*x*_ groups. Three different areas are distinguishable. Aromatic groups are located at δ_C_/δ_H_ 135–100/8.5–5.5 ppm, between δ_C_/δ_H_ 90–50/6.0–2.5 ppm signals of the aliphatic side chains can be assigned, and at even lower chemical shift values, aliphatic components are present. In Figure 3, the HSQC spectrum of F3 is shown.

The spectrum confirms that the lignin is composed only of G units and, hence, originates from softwoods. Additionally, three different types of linkages are identified: β-O-4′ (A), β–β′ (B), and β-5′ (C), which are also characteristic for softwood lignins. In the aromatic region, signals of oxidized G units (C_α_=O) can be identified. The signals between δ_C_/δ_H_ 80–75/3.5–4.0 ppm can be assigned to residual polysaccharides, which are still present in the samples. The HSQC spectra of all samples are presented and further compared in S5 of the Supporting Information.

### 3.2 Solid-State NMR Spectroscopy

**Figure 4:**
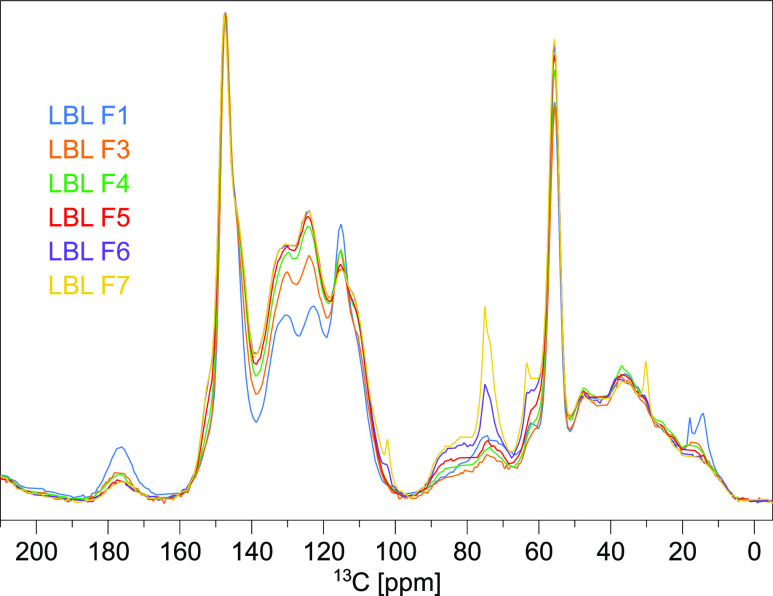
Stacked plots of all ^13^C-CP/MAS NMR spectra of F1 and F3–F7, D1 = 3.0 s, P15 = 7.0 ms.

When preparing the samples for the liquid-state NMR analyses, it was noticeable that the solubility of the lignins with increasing fraction deteriorated. Hence, not all of the components could be identified. Therefore, solid-state ^13^C—CP/MAS NMR analyses were performed. Figure 4 gives a stacked plot of the spectra of all analyzed fractions scaled to the signal with the highest intensity at 148 ppm. A more detailed analysis of the most important signals is presented in Table S7 in S8. F1 shows the highest content of carbonyl groups (signal at 177 ppm). In the aromatic region (150–110 ppm), samples F4, F5, F6, and F7 show similar signal intensities. In contrast, F1 shows different trends as the signal at 115 ppm has a higher intensity than the signals at 124 and 130 ppm. The signal at 115 ppm is caused by C_Ar-H_, the C_5_ atom of G units with β-O-4′ and β–β′ linkages, as well as by the C_2_ atom within a carboxy group in position 1.^66^ Hence, the high relative intensity of this signal correlates with the higher content of carboxy groups (see 177 ppm).^66,67^ In the aliphatic region between 80 and 30 ppm, the signal intensity of the methoxy group at 55 ppm is similar in all samples. The signal intensity at 75 ppm (C_α_ and C_β_ atoms of β-O-4′ linkages) increased in samples F6 and F7. With an increasing fraction number, the amount of linkages increases, which leads to bigger molecules, resulting in incomplete solubility in DMSO-*d*_6_, as seen in the liquid-state NMR analyses.

^1^H,^13^C-HETCOR-NMR spectra were recorded to show the heteronuclear correlation between ^1^H and ^13^C. Depending on the contact time, intermolecular couplings with a distance up to 6 Å can be examined.^25^ The spectra of F3 with a contact time of 200 μs and 3 ms are presented in S9 (Figure S14). At the short contact time, only correlations between carbons and hydrogens of the aromatic region as well as of the methoxy group and aliphatic side chain can be observed. By increasing the contact time, aromatic and aliphatic atoms correlate through space, and additionally, correlation peaks between C_Ar-O_ and different hydrogens (aromatic and aliphatic) occur, confirming the network structure of the lignin.

### 3.3 FT-ICR-MS Analysis of Soluble and Less-Soluble Lignin Compounds

**Figure 5:**
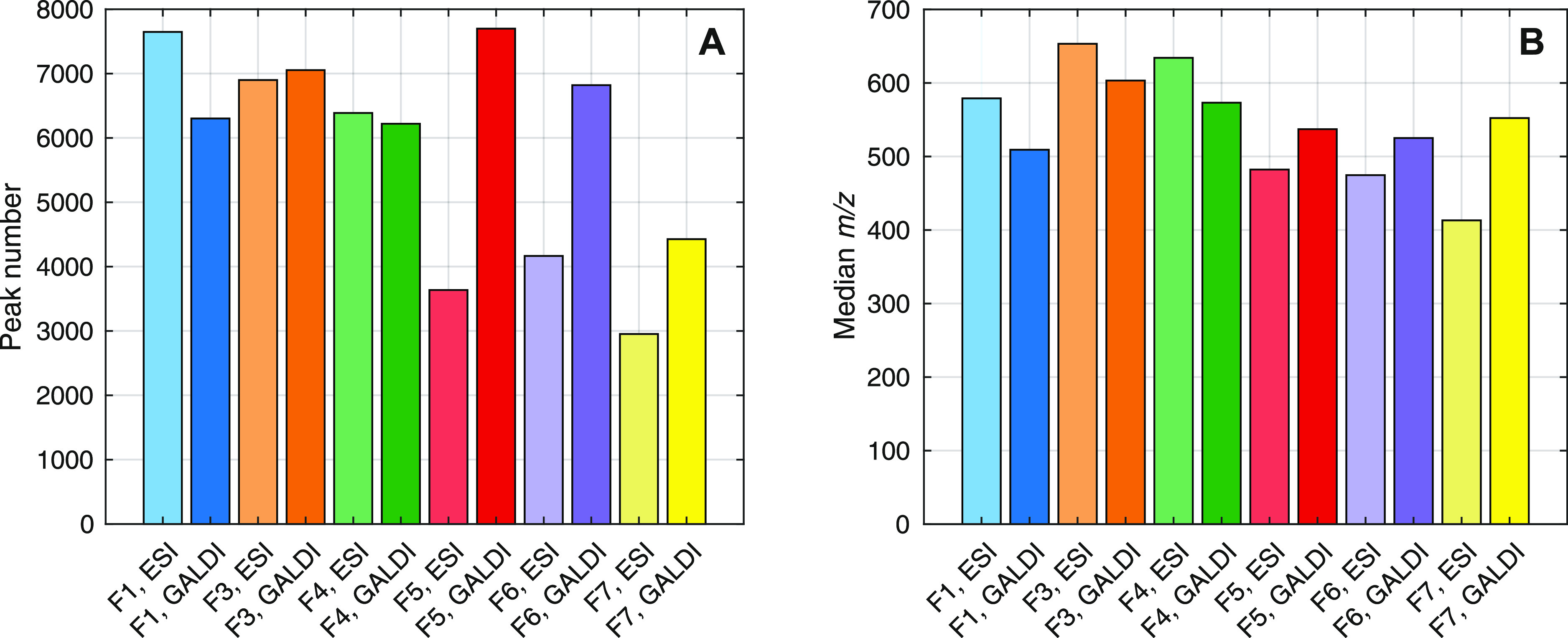
Comparison of mass spectral results for ESI- and GALDI-FT-ICR-MS analyses of the six different LBL fractions. (A) Peak number and (B) median *m*/*z*.

In order to obtain information about the molecular mass distribution of the six fractions, as well as further compositional information, all samples were analyzed by means of FT-ICR-MS. More specifically, ESI(−)-FT-ICR-MS was applied to characterize soluble analytes, and GALDI(−)-FT-ICR-MS was used to obtain information about less-soluble or insoluble molecules. The mass spectra for all six LBL fractions, analyzed by ESI- and GALDI-MS, are presented in the Supporting Information in S10 (see Figures S15 and S16). Additionally, the peak number and median *m*/*z* value comparisons were conducted with these data sets. The results of this data evaluation are visualized in Figure 5.

According to Figure 5, peak numbers and median *m*/*z* values for fractions F1–F4 are higher, if ESI-MS is utilized. This trend is completely inverted for fractions F5–F7, as the GALDI-MS data sets show considerably higher peak numbers and median *m*/*z* values. From these results, we can assume that the amount of smaller lignin compounds, which possess a higher polarity due to the presence of terminal functional groups (methoxy, carboxy, hydroxy, sulfate, sulfonate, etc.), decreases with an increasing fraction number (i.e., amount of acetone in the solvent mixture). Larger, less-polar lignin oligomers can be assumed in fractions F5–F7, according to the GALDI(−)-FT-ICR-MS data. The solubility of the lignin compounds seems to decrease with an increasing fraction number, as the high differences between GALDI-MS and ESI-MS results show, confirming the conclusions of the comparison of solution and solid-state NMR investigations. Thus, GALDI-FT-ICR-MS seems to be more appropriate to analyze all fractions of a fractionated lignin sample. Nonetheless, a more sensitive characterization of soluble lignin molecules is possible by using ESI-FT-ICR-MS. This is emphasized by the fact that the ESI-MS mass spectra (Figure S15) show higher intensities for individual ions, whereas GALDI-MS mass spectra (Figure S16) possess less-abundant peaks but in a broader *m*/*z* range.

**Figure 6:**
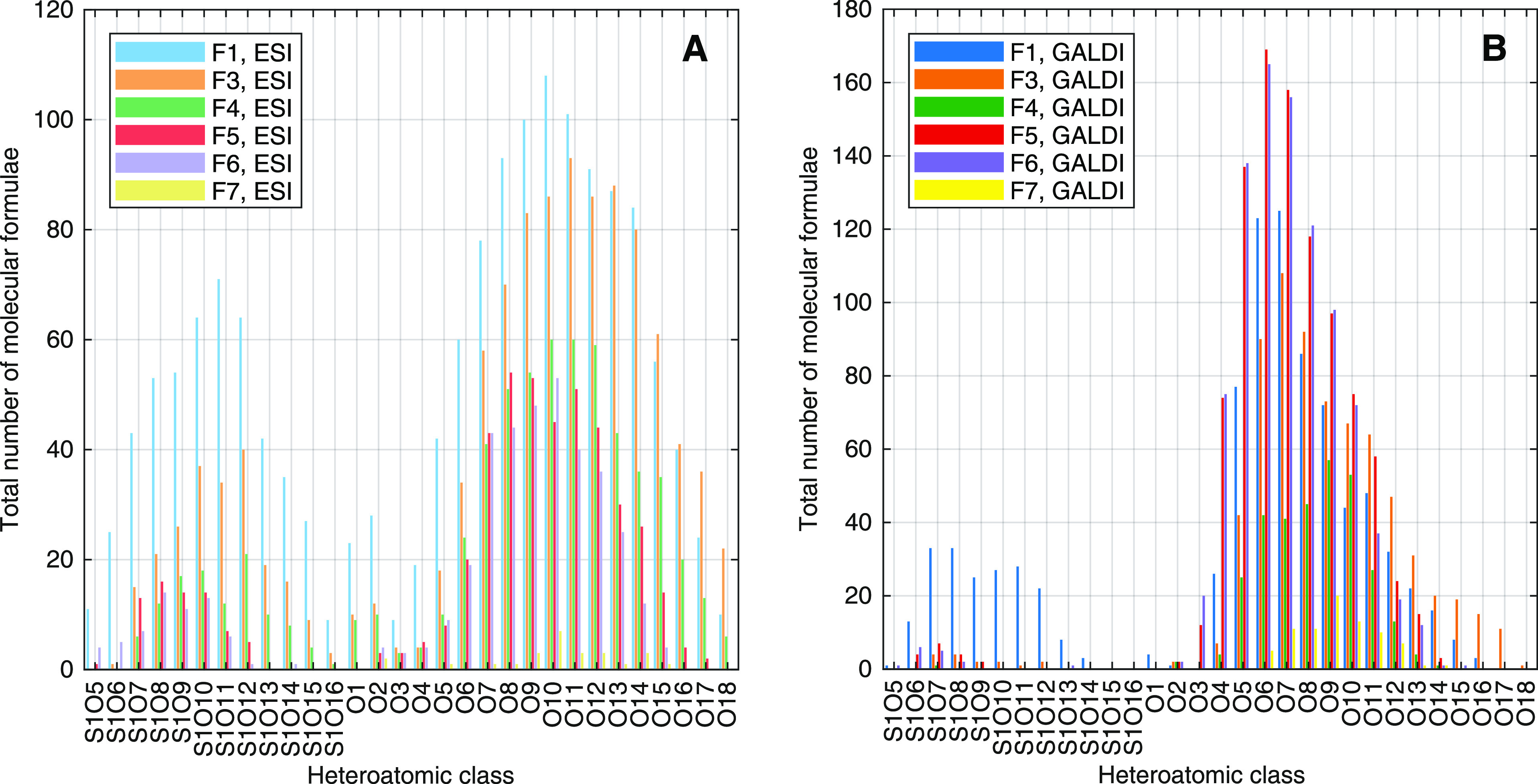
Comparison of the assigned number of molecular formulas to the FT-ICR-MS data sets of the six LBL fractions. (A) ESI and (B) GALDI.

In a next step, the molecular formulas, which were assigned to each ion’s *m*/*z* in the data evaluation process, were further evaluated with the help of statistical plots. For this purpose, assigned molecular formulas were clustered into heteroatomic classes, according to the number of nitrogen, oxygen, and/or sulfur, and these clusters were illustrated with regard to the total number of assigned molecular formulas and relative abundance of each class. The visualization for the number of assigned molecular formulas for heteroatomic classes S_1_O_5_–S_1_O_16_ and O_1_–O_18_ is presented in Figure 6. In addition, a bar chart plot of the relative abundancies for the same classes can be found in S11 (Figure S17) in the Supporting Information.

According to the visualized statistical plots, most molecular formulas and highest relative abundancies for sulfur- and oxygen-containing heteroatomic classes S_1_O_5_–S_1_O_16_ are observable for fractions F1 and F3. In all other fractions, only minor amounts of sulfur- and oxygen-containing compounds can be supposed. Thus, a separation of these molecules from the rest of the LBL sample in the first fractions seems obvious. Most of these compounds from fractions F1 and F3 also seem to possess a comparably high solubility and polarity because the highest numbers of assigned molecular formulas and relative abundancies are observable for the ESI-MS data sets of these two samples.

Oxygen-containing molecules with up to 18 oxygen atoms were assigned to the MS data sets of the analyzed fractions. Highest numbers of molecular formulas and relative abundancies for molecules with *o* ≤ 2 are observed for samples F1 and F3 in the ESI(−)-FT-ICR-MS data sets. Compounds with medium oxygen values (3 ≤ *o* ≤ 10) are mainly assigned to the GALDI-MS data sets of all six fractions. Molecules with oxygen numbers of 11 and higher are predominantly found in the ESI(−)-FT-ICR-MS data sets of samples F1–F5. Thus, these molecules seem to possess a high amount of polar functional groups (methoxy, carboxy, hydroxy, etc.) as they are still soluble, despite their considerable molecular size. A similar trend was also observed for different Kraft and LignoBoost lignin samples, as demonstrated in a previous work^50^ from our research group. Hence, the results of the LBL fractions, once again, show the necessity to combine ESI- and GALDI-MS analyses to obtain realistic and comprehensive insights into complex biomass samples.

Interestingly, we were also able to observe molecules in heteroatomic classes N_1_O_3_–N_1_O_5_ and N_1_O_15_–N_1_O_21_ for sample F7 using GALDI(−)-FT-ICR-MS (not shown here). Most likely, these compounds are nonpolar nitrogen-containing molecules, which could not be diluted by the water–acetone solvent mixtures and are therefore detectable in the insoluble residue of the LBL sample.

In order to achieve more detailed information about the potential molecules that can be detected in the different fractions by the two FT-ICR-MS methods, *n*_C_-DBE plots can be utilized. Plots of the heteroatomic classes O_7_–O_10_ as well as sulfur-containing classes S_1_O_9_–S_1_O_12_ for all analyzed fractions are visualized in Figures S18 and S19. According to these plots, comparable lignin oligomer sizes are assumable for each sample with the two different MS methods. The *n*_C_-DBE plots, which were generated from the GALDI-MS data sets, in general show more molecules in a broader *n*_C_ and DBE range for the illustrated compound classes. Hence, GALDI-MS seems to help to analyze larger lignin oligomers with an oxygen number of 7–10, which are less polar and less soluble. According to the plots of both MS methods, ESI- and GALDI-FT-ICR-MS, an increase in the *n*_C_ and DBE ranges with an increasing oxygen number is observable. This indicates that the molecular sizes of detected lignin oligomers also increase with increasing oxygen numbers. For samples F3 and F4, a maximum DBE value of 31 is observable in compound class O_10_, which is indicative for oligomer sizes up to pentamers. Even larger oligomers up to octamers are imaginable for the higher oxygen-containing heteroatomic classes (*o* ≤ 18). Considerably lower DBE values (DBE ≤ 24) are observable for the ESI-MS data sets for all analyzed fractions (see Figure S18) for compound classes O_7_–O_10_, which speaks for lignin compounds that contain up to two aromatic units less than the molecules, which were detected by GALDI-FT-ICR-MS. The smallest potential lignin oligomers, which can be detected by our FT-ICR-MS methods, comprise monomers and dimers, according to the *n*_C_-DBE plots of compound classes with *o* ≥ 2 (not shown here).

The lowest number of data points is observable for F7, if the *n*_C_-DBE plots for all analyzed samples are compared (see Figures S18 and S19). In the ESI-MS data sets, only small numbers of molecules are observable for the heteroatomic classes O_7_–O_10_ and S_1_O_9_–S_1_O_12_. The number of molecules is drastically larger in the GALDI-MS data sets, at least for compound classes O_7_–O_10_, which illustrates, once again, the potential of this ionization method to ionize larger compounds with less-polar functionalities.

Further information on the different compounds and compound classes, which are contained in the six fractions, are obtainable by using van Krevelen plots.^68^ Illustrations of these plots for the LBL samples, analyzed by ESI-MS and GALDI-MS, are presented in the Supporting Information (Figure S20). These plots show that besides lignin compounds, also condensed hydrocarbons, lipids, peptides, amino sugars, and carbohydrates can be supposed in most fractions. Interestingly, none of these potential impurities are vanishing with an increasing fraction number, according to the GALDI(−)-FT-ICR-MS data sets. Hence, one can assume that also larger condensed hydrocarbon, lipid, peptide, amino sugar, or carbohydrate impurities are fractionated together with lignin oligomers, whose molecular size increases with an increasing fraction number (i.e., amount of acetone in the solvent mixture).

### 3.4 Tandem MS Analysis of Selected Lignin Molecular Ions

Comprehensive structural characterizations of lignin molecules usually require at least a second mass spectrometric analysis. Hence, we conducted MS/MS experiments by utilizing CID as an ion activation method to obtain more structural information about specific molecular lignin ions. In total, 60 different molecular ions from all six samples in an *m*/*z* range between 290 and 820 Da that showed high intensities were quadrupole-selected and fragmented by ESI-MS/MS and GALDI-MS/MS. These MS/MS analyses revealed that most of the selected precursor ions predominantly consist of G units, which are connected via β-5′ bonds (absence of [M–H–H_2_O–CH_2_O]^−^ fragment ion). The fragmentation patterns for most of the molecular ions also indicate to a presence of methoxy (detection of [M–H–CH_3_^•^]^−^ and [M–H–2CH_3_^•^]^−^ ions) and carboxy groups (detection of [M–H–CO_2_]^−^ and [M–H–HCOOH]^−^ ions), as well as for some ions, to the presence of primary hydroxy groups (detection of [M–H–OH]^−^, [M–H–H_2_O]^−^ and [M–H–CH_2_O]^−^ ions).^29,35,47^ Thus, these MS/MS results are in good alignment with the results of the ^1^H- and ^13^C NMR analyses of the six fractions.

Structural suggestions for four exemplary precursor ions [493.15 Da (C_27_H_25_O_9_, [M – H]^−^), 507.17 Da (C_28_H_27_O_9_, [M – H]^−^), 567.19 Da (C_30_H_31_O_11_, [M – H]^−^), and 627.22 Da (C_36_H_35_O_10_, [M – H]^−^)] are presented in S14. These suggestions are based on the detected fragment ions, which are indicative for specific structural subunits and functional groups.

In order to compare the different samples and two ionization methods with each other, a molecular ion with *m*/*z*_theoretical_ = 507.166056 Da (C_28_H_27_O_9_, [M – H]^−^) was quadrupole-selected (507.17 Da) in each fraction and analyzed by ESI-MS/MS and GALDI-MS/MS. This process should help us to understand how the positions and amounts of functionalities for a specific lignin molecule change with ongoing fractionation. In addition, the tandem MS data of all characterized precursor ions were evaluated with the help of correlation and cluster analysis. For this purpose, the presence of 17 different fragment ions was investigated, which are typically formed during fragmentation, if certain lignin linkages and functionalities^29,35^ (see Table S8 for more information) are present. In the next step, all tandem MS spectra were searched for these 17 fragment ions. If a matching *m*/*z* was found, the observed and relative intensities were automatically transferred to a MATLAB matrix. This matrix was the basis for the chemometric evaluation as it allowed us to compare the types of fragment ions that are observable for different precursor ions and ionization methods. Our aim was to identify specific lignin substructures and functionalities that are detectable for different molecular ions. In this way, molecules with similar structural characteristics were found as the MS/MS data of these ions showed either high correlation coefficients (Pearson’s ρ, correlation analysis) or low distances from each other in the dendrogram (cluster analysis).

**Figure 7:**
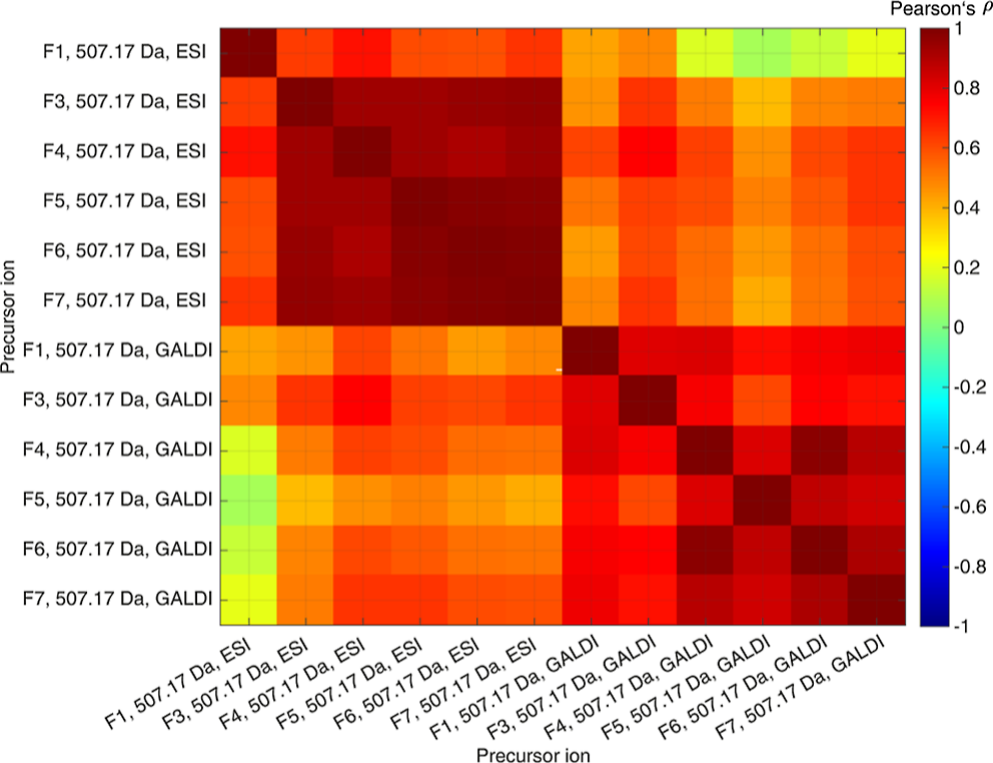
Correlation heatmap of the ESI-MS/MS and GALDI-MS/MS analyses of the precursor ion 507.17 Da (C_28_H_27_O_9_, [M – H]^−^) for all six LBL fractions. For this chemometric evaluation, the relative intensity information and a confidence interval of 99.9% were used.

Figure 7 illustrates the results of the correlation analysis of the tandem MS data for the precursor ion 507.17 Da (C_28_H_27_O_9_, [M – H]^−^), which was characterized in each sample with both ionization methods. For the data evaluation process, the relative intensities of all relevant fragment ions were compared with each other. The results of the cluster analysis of this chemometric data set are presented in Figure S22 in the Supporting Information. According to Figure 7, two areas of high correlation are observable for the precursor ion 507.17 Da. ESI-MS/MS spectral results of the different fractions are correlating intensively with each other, which is especially obvious for samples F5–F7 (ρ = 0.97–0.99). Hence, structurally similar compounds with a molecular formula of C_28_H_28_O_9_ can be supposed in most samples, according to the ESI-MS/MS data. These molecules are, most likely, lignin trimers with a relatively high number of oxygen-containing functionalities, which results in a high solubility in the polar ESI solvent. Only the correlation coefficients between F1 and the other five fractions in the ESI-MS/MS data set are significantly lower (ρ = 0.59–0.71). Hence, structurally different isomers of the molecule, which produces the molecular ion at *m*/*z* = 507.17 Da, seem to be separated at the first fractionation step, where the solvent mixture consisted of 90% H_2_O (v/v) and 10% acetone (v/v).

The correlation coefficients for the GALDI-MS/MS data sets are in a similar range in comparison to the ESI-MS/MS data sets (ρ = 0.61–0.97). These GALDI-MS/MS data are more alike among each other than compared to the ESI-MS/MS data. Thus, we can conclude that structurally different precursor ions are generated and fragmented by GALDI-MS/MS in comparison to ESI-MS/MS. The highest correlation coefficient for the GALDI-MS/MS data was observed between F4 and F6 (ρ = 0.97). If the results from the two ionization methods are compared, we can observe that the lowest correlation coefficient is observable for F1-ESI and F5-GALDI (ρ = 0.07) and the highest for F4-ESI and F3-GALDI (ρ = 0.74).

We have included a comparison of the detailed tandem MS results of F3-ESI and F5-GALDI (ρ = 0.38) in the Supporting Information (see S15, Table S8) to illustrate the different fragment ions, which are produced from the precursor ion 507.17 Da in various samples by ESI- or GALDI-MS/MS.

In addition to the chemometric evaluation of one specific precursor ion, also the results for the correlation analysis of the tandem MS data sets for all characterized fragment ions are presented in Figure S23. These results are helpful to identify precursor ions, which seem to possess comparable lignin subunits and functional groups. Thus, by this chemometric approach, structurally similar lignin compounds can be verified that differ mainly in their oligomeric size.

### 3.5 Correlation of NMR and FT-ICR-MS Results

**Figure 8:**
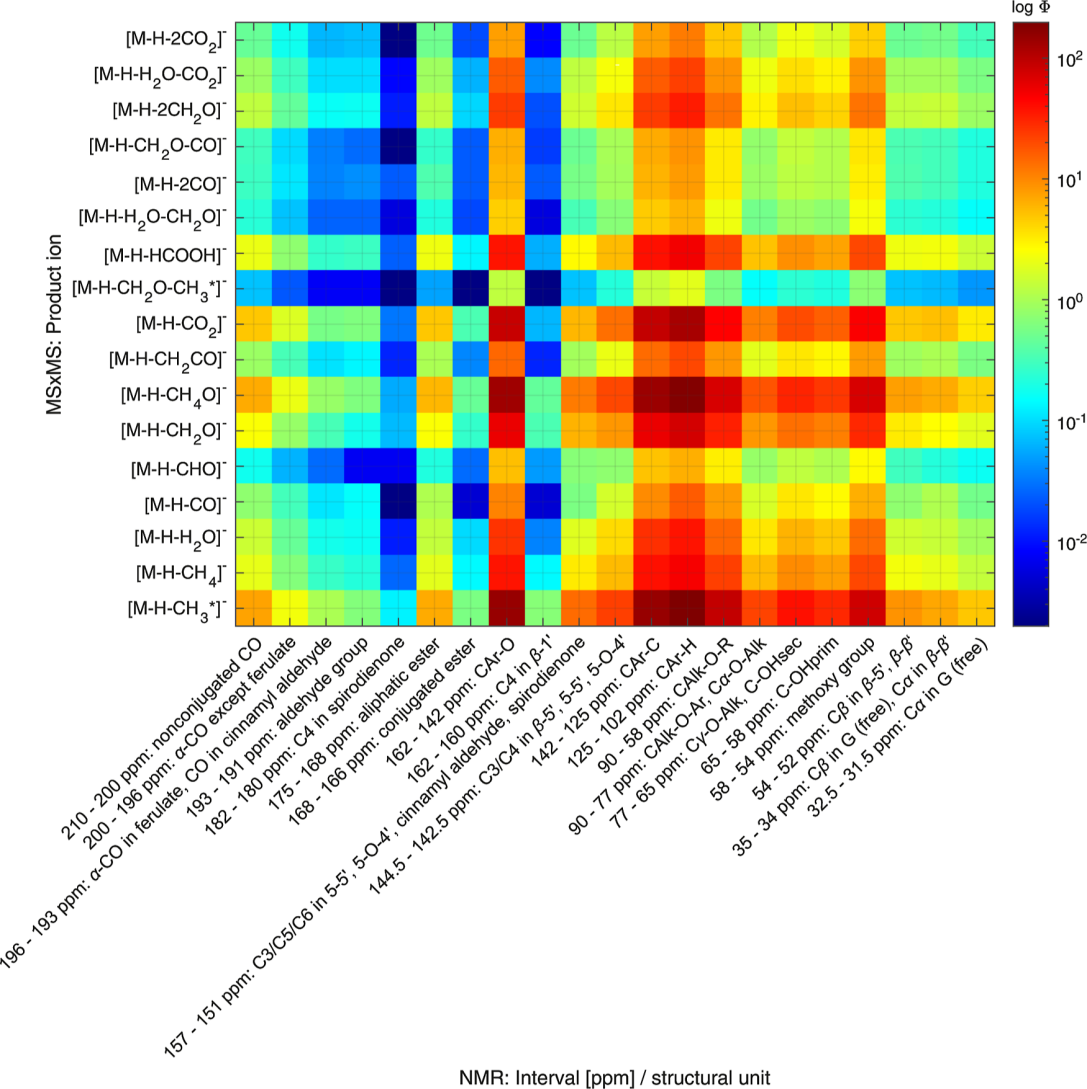
Correlation heatmap as a result of the hetero spectral combination of quantitative ^13^C NMR and ESI-MS/MS data sets. The calculated correlation coefficients Φ are presented logarithmic and color-coded.

The combination of NMR and FT-ICR-MS results by means of correlation spectroscopy was, in a first step, tested by using the quantitative ^13^C NMR results, i.e., the number of proposed chemical structures and functionalities per aryl unit (also see Table 1), and the ESI- or GALDI-MS/MS results for each characterized precursor ion of the six fractions. For this purpose, sums of the relative intensity values for each of the 17 lignin-typical fragment ions (also see Table S8) were calculated per fraction. Afterward, correlation matrices were calculated for each fraction, according to eq 1, by combining the quantitative NMR information with the tandem MS results. These correlation matrices for each fraction were then summed up to obtain a result matrix, which helped to evaluate and visualize general correlations between both data sets. The correlation heatmap for the combination of quantitative ^13^C NMR results with ESI-MS/MS data is presented in Figure 8. Results for the correlation of quantitative ^13^C NMR with GALDI-MS/MS results are illustrated in Figure S24.

Both figures illustrate the potential of the hetero spectroscopic correlation approach in a condensed and informative way. From these heatmaps, we are able to derive correlations between lignin-typical fragment ions and NMR chemical shift values, which are also indicative for certain linkages and functionalities. Thus, we are able to identify lignin-typical subunits and functional groups, which are the main reason for the production of specific fragment ions. One obvious trend that is visible in both heatmaps is a relatively high correlation of the NMR shift region for C_Ar-O_ units (162–142 ppm), which are specific for C–O–C bonds at an aryl unit, with the [M–H–CH_3_^•^]^−^ and [M–H–CH_4_O]^−^ fragment ions. This observation can be attributed to a presence of terminal methoxy groups at the lignin structure. The high correlation coefficients for these fragment ions and the C_Ar–H_ NMR shift region (125–102 ppm) emphasize this statement. In addition, a high correlation between the C_Ar–C_ region (142–125 ppm) and the [M–H–CO_2_]^−^ fragment ion can be attributed to a high amount of carboxy groups that are present in the LBL structure.

Low correlations were found between most of the MS/MS fragment ions and the integrals, which are assigned to aldehyde groups (193–191 ppm), C_4_ in spirodienone (182–180 ppm), conjugated esters (168–166 ppm), and C_4_ in β-1′ linkages (162–160 ppm, typical for 1,2-diaryl-1,3-propanediol structures).^35^ Hence, as also shown by the ^1^H NMR data, the latter structural units are present in only small quantities in the analyzed samples. It is more likely that the LBL structure in general is composed of G units, which are mainly connected via β-5′ bonds. However, also β-O-4′ and β–β′ linkages are partially present. Functional groups at the aromatic core structure are mainly methoxy, carboxy, and primary hydroxy groups. Thus, the chemometric combination of NMR and tandem MS results can help to comprehensively study the overall structure of complex biopolymer samples.

Additional results of the hetero spectroscopic correlation are presented in the Supporting Information for the combination of ^1^H NMR data with ESI-MS/MS (Figure S25) and GALDI-MS/MS (Figure S26) data.

**Figure 9:**
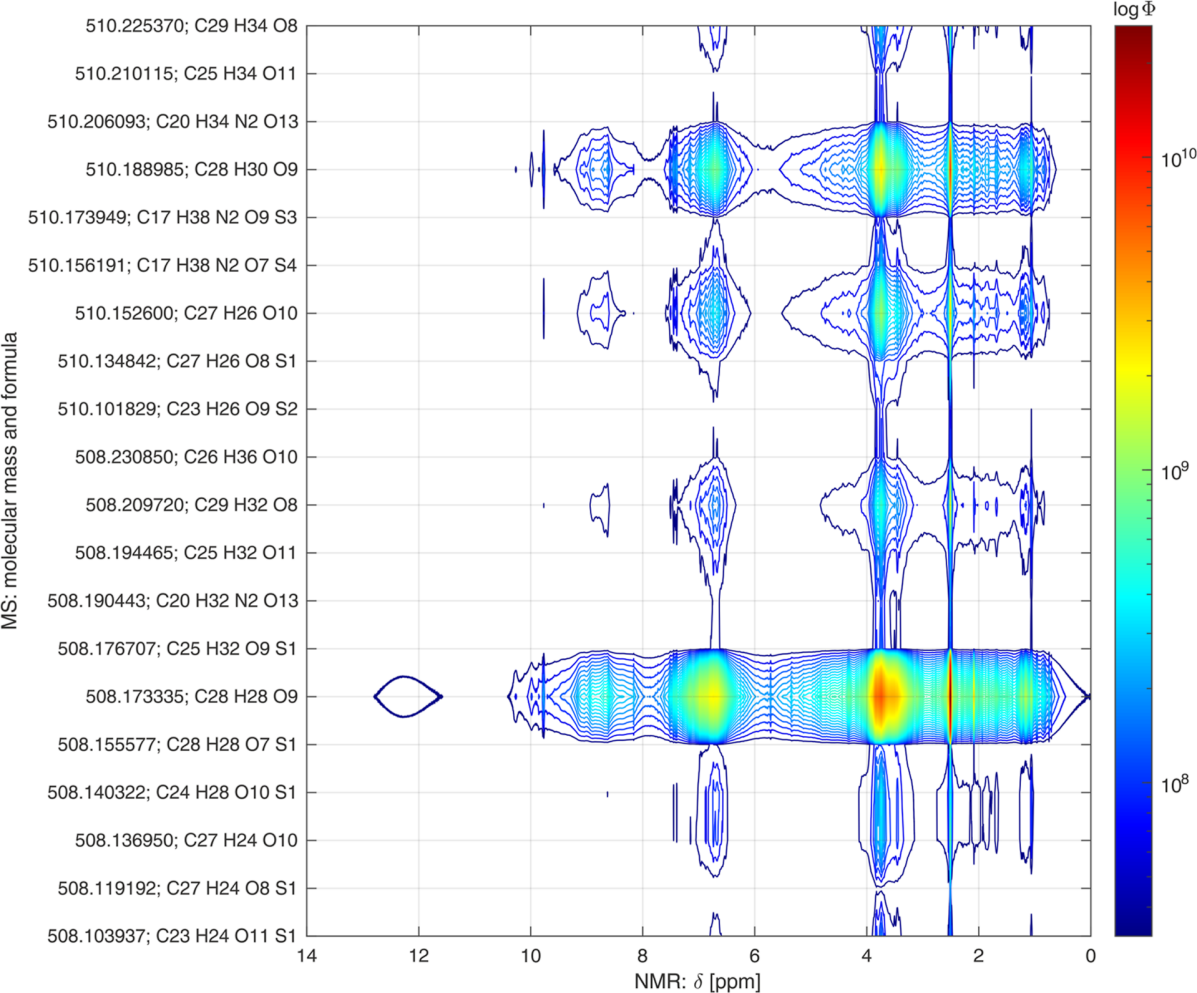
Contour plot as a result of the hetero spectral combination of ^1^H NMR and ESI-MS data sets in a mass range from 508 to 510 Da. *m*/*z* values and ion formulas were proton- and electron-corrected to obtain molecular masses and formulas. The calculated correlation coefficients Φ are presented logarithmic and color-coded.

More compound-specific structural information are obtainable, if the hetero spectroscopic correlation approach is extended to 1D-MS and NMR data. From the detected *m*/*z* values, we can calculate molecular formulas for each ion. If this information is correlated with the NMR information throughout a set of samples, we can verify specific functional groups or linkages in the molecular structure without the necessity for tandem MS experiments. The results of this correlation approach are visualized in Figure 9 for the combination of ESI-FT-ICR-MS and ^1^H NMR data sets, as well as in Figure S27 of the Supporting Information for a combination of GALDI-FT-ICR-MS and ^1^H NMR data sets.

In both figures, proton- and electron-corrected masses and molecular formulas are used to characterize the structure of molecules, which are present in the LBL fractions. We were able to detect 3384 different molecular ions in all analyzed fractions by ESI-MS and 4246 molecular ions by GALDI-MS, which shows the potential depth of information for this kind of data combination. Hence, we decided to evaluate only a small fraction of the mass spectral data. For example, in Figure 9, the correlation results for the ESI-MS and ^1^H NMR data sets are only presented in a mass range from 508 to 510 Da. In general, similar plots are possible for the entire mass range, which enables a basic structural characterization of all detected LBL compounds.

According to the correlation results presented for the combination of ESI-FT-ICR-MS and ^1^H NMR data sets, most MS signals correlate with NMR signals that are originating from aromatic protons (6–8 ppm) as well as methoxy groups (∼4 ppm). Thus, as already expected, these structural subunits can be verified for most molecules in the evaluated mass range. In contrast, correlation peaks for NMR signals, which are attributable to free phenolic hydroxy groups (∼9 ppm), are only visible for molecules with molecular formulas of C_28_H_28_O_9_ (508.173335 Da), C_29_H_32_O_8_ (508.209720 Da), C_27_H_26_O_10_ (510.152600 Da), and C_28_H_30_O_9_ (510.188985 Da). For the molecule C_28_H_28_O_9_ (508.173335 Da), an additional correlation peak can be observed at an NMR shift region of ∼12 ppm, which is specific for hydroxy groups in a carboxy group. The relatively broad correlation peak is produced by the broad NMR signal of this functional group due to a chemical exchange of the mobile protons. Hence, for the four molecules that show a high correlation within the carboxy-specific NMR shift regions, at least one terminal carboxy group can be verified. Furthermore, correlation peaks that are located at an NMR shift region of ∼10 ppm are attributable to terminal aldehyde groups, which are potential functional groups for the molecules C_28_H_28_O_9_ (508.173335 Da), C_27_H_26_O_10_ (510.152600 Da), and C_28_H_30_O_9_ (510.188985 Da).

The intensive correlation peaks that are located at an NMR shift region of ∼2.50 and 1.67–0.86 ppm can be attributed to signals from the solvent (DMSO-*d*_6_) and hydrocarbon compounds in the LBL sample/fractions. As correlations between these NMR signals and all detected molecules are observable, these correlation peaks can be considered as background peaks of the hetero spectroscopic correlation spectrum. Theoretically, these solvent signals could also be filtered out during the data evaluation process. Furthermore, the water signals in the NMR spectra could also be lowered if the samples are further dried or molecular sieves are utilized.

## 4 Conclusions

In this research work, a multispectroscopic approach, utilizing FT-ICR-MS and NMR spectroscopy, was applied to structurally characterize a fractionated LignoBoost lignin. For this purpose, liquid-state and solid-state ^1^H- and ^13^C NMR was used to determine the different linkages and functional groups, which are present in the overall lignin structure. ESI- and GALDI-FT-ICR-MS as well as MS/MS analyses helped to achieve further information on the molar mass distribution, oligomer sizes, and structural characteristics (linkages, functional groups, etc.) of the different lignin compounds contained in each LBL fraction.

In a second data evaluation step, a hetero spectroscopic correlation approach was utilized for a chemometric combination of the ^1^H- and ^13^C NMR results with the FT-ICR-MS and tandem MS data sets. The correlative combination of tandem MS and NMR data helped to understand the general structure of the fractionated and analyzed LignoBoost lignin. Thus, we were able to conclude that this lignin is mainly composed of G units, which are connected via β-5′ linkages. Furthermore, also small quantities of β-O-4′ and β–β′ bonds are verifiable. The most dominant terminal groups at the lignin structure are methoxy, carboxy, and primary hydroxy groups. In order to extend these general information to the structural characterization of individual lignin compounds, also a hetero spectroscopic correlation of NMR and 1D-MS data sets was applied. From these correlation analyses, it became obvious that this data combination helps to obtain compound-specific structural information. For instance, specific functional groups or linkages can be verified for a defined molecular formula, which can be utilized to describe the structural composition of a single lignin molecule without the need for tandem MS experiments.

Hence, with the help of our developed NMR, FT-ICR-MS, and combinatoric methods, it is possible to not only structurally characterize LignoBoost lignins but, hopefully, also other native, isolated, or modified biopolymers. The additional knowledge about the lignin structure, depending on its origin and reprocessing, will help to realize more efficient and expedient modification processes for biopolymers. These optimized processes will significantly aid in the production of lignin- and lignocellulose-based products of higher value in the near future. Thus, we hope that our research work can help to stimulate this transition of lignin and other biopolymers from an energetic source to a potential source for base chemicals and other material-based applications.

## Supporting Information Available

Liquid-state ^1^H-, quantitative ^13^C- and ^1^H, ^13^C-, ^1^H–^13^C HSQC-NMR spectra of all samples; ESR analyses of the DMSO solutions; optimization of solid-state ^13^C-CP/MAS NMR using F3; ^1^H–^13^C HETCOR spectra of F3; ^13^C-CP/MAS NMR spectra, solid-state ^13^C-SP-MAS NMR of F3; ESI-MS, and GALDI-MS mass spectra of the six analyzed fractions; comparison of relative abundancies for oxygen-containing and sulfur-/oxygen-containing molecular formula classes to the FT-ICR-MS data sets of the six different fractions; *n*_C_-DBE plots of oxygen- and sulfur-/oxygen-containing compound classes for all six fractions; Van Krevelen plots of the ESI- and GALDI-FT-ICR-MS data sets for all six fractions; structural suggestions for exemplary lignin compounds; further chemometric evaluation of tandem MS analyses of all six fractions; and additional hetero spectroscopic correlation results (PDF)
