# Supplementary material for: Chemometric Combination of Ultrahigh Resolving Mass Spectrometry and Nuclear Magnetic Resonance Spectroscopy for a Structural Characterization of Lignin Compounds
Source: ACS Omega. 2023 Dec 19;9(1):628–41. doi: 10.1021/acsomega.3c06222 (PMC10785065; doi:10.1021/acsomega.3c06222)
Supplement: Supplementary file 1 — ao3c06222_si_001.pdf [file ao3c06222_si_001.pdf]

SUPPORTING INFORMATION

Chemometric Combination of Ultrahigh Resolving Mass Spectrometry and Nuclear Magnetic Resonance Spectroscopy for a Structural Characterization of Lignin Compounds

Lara Dütsch[a], Klara Sander[a], Erica Brendler[a], Martina Bremer[b], Steffen Fischer[b], Carla Vogt[a], and Jan Zuber[a,*]

[a] *Institute of Analytical Chemistry, TU Bergakademie Freiberg, Leipziger Straße 29, 09599 Freiberg, Germany*

[b] *Institute of Plant and Wood Chemistry, TU Dresden, Pienner Strasse 19, 01737 Tharandt, Germany*

** Corresponding Author, E-Mail: Jan.Zuber@chemie.tu-freiberg.de*

Contents

S1. ^{1}H-NMR spectra of the analyzed samples F1 and F3 - F7 . . . . **S-4**

S2. Electron spin resonance (ESR) spectroscopy of all six analyzed samples, dissolved in DMSO-d$_6$ **S-6**

S3. Quantitative ^{13}C-NMR spectra of the analyzed samples F1 and F3 - F7 . . . . **S-7**

S4. Quantification of the ^{13}C-NMR spectra of F1 and F3 - F7 . . . . **S-9**

S5. ^{1}H,^{13}C-HSQC-NMR spectra of the analyzed samples F1 and F3 - F7 . . . . **S-14**

S6. Optimization of repetition time D1 and contact time P15 for ^{13}C-CP/MAS-NMR . . **S-16**

S7. ^{13}C-SP/MAS-NMR of sample F3 . . . . **S-18**

S8. ^{13}C-CP/MAS-NMR of the analyzed samples F1 and F3 - F7 . . . . **S-19**

S9. ^{1}H,^{13}C-HETCOR-NMR spectroscopy of sample F3 . . . . **S-21**

S10. ESI-MS and GALDI-MS mass spectra of the six analyzed LBL fractions **S-22**

S11. Comparison of relative abundancies for oxygen-containing and sulfur-/oxygen-containing molecular formula classes to the FT-ICR-MS data sets of the six different LBL fractions **S-25**

S12. *n_C-DBE* plots of oxygen- and sulfur-/oxygen-containing compound classes for all six LBL fractions **S-26**

S13. Van Krevelen plots of the ESI- and GALDI-FT-ICR-MS data sets for all six LBL fractions **S-29**

S14. Structural suggestions for exemplary lignin compounds **S-31**

S15. Further chemometric evaluation of tandem MS analyses of all six LBL fractions . . **S-32**

S16. Additional hetero spectroscopic correlation results **S-36**

List of Figures

Figure S1. ^{1}H-NMR spectra of the samples F1 and F3 - F7 in DMSO-d$_6$, recorded on a 700 MHz spectrometer. S-4
Figure S2. ^{1}H-NMR spectra of F1 and F3 - F7. . . . . S-5
Figure S3. ESR spectra of F1 and F3 - F7. . . . . S-6
Figure S4. Quantitative ^{13}C-NMR spectrum of F3 with integral regions. S-7
Figure S5. Quantitative ^{13}C-NMR spectra of the samples F1 and F3 - F7 in DMSO-d$_6$, recorded on a 700 MHz spectrometer. S-8
Figure S6. Comparison of the integrals of the aromatic carbons of F1 and F3 - F7. . . . . S-11
Figure S7. Comparison of the integrals of different linkages as well as different types of oxygen-substituted carbons of F1 and F3 - F7. . . . . S-12
Figure S8. ^{1}H,^{13}C-HSQC-NMR spectra of all samples F1 and F3 - F7, recorded on a 700 MHz spectrometer. S-14
Figure S9. Optimization of D1 for F3. Spectra are scaled to the intensity of the MeO group (55.6 ppm). S-16
Figure S10. Optimization of P15 for F3. Spectra are scaled to the intensity of the MeO group (55.6 ppm). S-17
Figure S11. ^{13}C-SP/MAS-NMR spectra of F3 (blue), an empty zirconium dioxide rotor with a PTFE cap (red) and the difference spectrum after subtracting the red spectrum. Spectra were recorded on a 700 MHz spectrometer and the image was created using *Topspin (version 4.1.3)*. S-18
Figure S12. ^{13}C-CP/NMR spectrum of F1 with the chemical shifts of the most important peaks. . S-19
Figure S13. ^{13}C-CP/MAS-NMR spectra of the analyzed samples F1 and F3 - F7, D1 = 3.0 s, P15 = 7.0 ms, recorded on a 400 MHz spectrometer. S-20
Figure S14. ^{1}H,^{13}C-HETCOR-NMR spectra of F3, A) contact time 200 µs, B) contact time 3.0 ms. S-21
Figure S15. ESI(−)-FT-ICR-MS mass spectra of fractions F1 and F3 - F7. . . . . S-22
Figure S16. GALDI(−)-FT-ICR-MS mass spectra of fractions F1 and F3 - F7. . . . . S-23
Figure S17. Comparison of relative abundancies of molecular formula classes S_1O_5 - S_1O_{16} and O_1 - O_{18} assigned to the FT-ICR-MS data sets of the six fractions. A - ESI, B - GALDI. . . . S-25
Figure S18. n_C-*DBE* plots of compound classes O_7 - O_{10} for all six fractions, which were analyzed by ESI- and GALDI-FT-ICR-MS. The observed intensity is presented logarithmic and color-coded (blue: low intensity, yellow: medium intensity, red: high intensity). S-27
Figure S19. n_C-*DBE* plots of compound classes S_1O_9 - S_1O_{12} for all six fractions, which were analyzed by ESI- and GALDI-FT-ICR-MS. The observed intensity is presented logarithmic and color-coded (blue: low intensity, yellow: medium intensity, red: high intensity). S-28
Figure S20. Van Krevelen plots for all six fractions, which were analyzed by ESI- and GALDI-FT-ICR-MS. The observed intensity is presented logarithmic and color-coded (blue: low intensity, yellow: medium intensity, red: high intensity). S-30

Figure S21. Structural suggestions for four different lignin compounds, which were characterized by ESI- and GALDI-MS/MS. A - 493.15 Da ($C_{27}H_{25}O_9$, $[M\text{-}H]^-$), B - 507.17 Da ($C_{28}H_{27}O_9$, $[M\text{-}H]^-$), C - 567.19 Da ($C_{30}H_{31}O_{11}$, $[M\text{-}H]^-$), D - 627.22 Da ($C_{36}H_{35}O_{10}$, $[M\text{-}H]^-$). S-31
Figure S22. Dendrogram of the hierarchical cluster analysis of the ESI- and GALDI-MS/MS data sets of the six samples for precursor ion 507.17 Da ($C_{28}H_{27}O_9$, $[M\text{-}H]^-$), using the relative intensity information of selected fragment ions. For the cluster analysis, Euclidean distance metric and Ward linkage for distance computing were applied. S-32
Figure S23. Correlation heatmap of the ESI-MS/MS and GALDI-MS/MS analyses of 60 precursor ions for all six fractions. For this chemometric evaluation the relative intensity information and a confidence interval of 99.9 % was used. S-35
Figure S24. Correlation heatmap as result of the hetero spectral combination of quantitative ^{13}C-NMR and GALDI-MS/MS data sets. The calculated correlation coefficients Φ are presented logarithmic and color-coded. S-36
Figure S25. Correlation heatmap as result of the hetero spectral combination of ^{1}H-NMR and ESI-MS/MS data sets. The calculated correlation coefficients Φ are presented logarithmic and color-coded. S-37
Figure S26. Correlation heatmap as result of the hetero spectral combination of ^{1}H-NMR and GALDI-MS/MS data sets. The calculated correlation coefficients Φ are presented logarithmic and color-coded. S-38
Figure S27. Contour plot as result of the hetero spectral combination of ^{1}H-NMR and GALDI-MS data sets in a mass range from 505 to 506 Da. *m/z* values and ion formulae were proton and electron corrected to obtain molecular masses and formulae. The calculated correlation coefficients Φ are presented logarithmic and color-coded. S-39

List of Tables

Table S1. Assignment of the characteristic regions in the ^{1}H-NMR spectrum. S-5
Table S2. Assignment of the most important peaks in Figure S4. . . . . . S-7
Table S3. Assignments of the structural units and their quantification (in amount per C_9 unit, exemplary for F3), in reference to Capanema et al.[15] S-9
Table S4. Integrals of the selected regions of the quantitative ^{13}C-NMR spectra of all analyzed fractions. S-10
Table S5. Average integration errors for the quantitative ^{13}C-NMR analyses of all LBL fractions, in reference to Capanema et al.[15] S-13
Table S6. Assignments of the signals of the HSQC-NMR spectrum of F3. . . . . . S-15
Table S7. Assignments of the signals of the solid-state ^{13}C-NMR spectrum.[66,67] S-19
Table S8. Comparison of observable fragment ions for precursor ion 507.17 Da ($C_{28}H_{27}O_9$, $[M\text{-}H]^-$) in data sets of samples F3 - ESI and F5 - GALDI. S-33

Important notice

As already mentioned in the main part of the paper, LBL fraction F2 could not be analyzed by means of NMR and FT-ICR-MS due to its low sample amount. Thus, all analyses results, which are presented in this Supporting Information file, only comprise results for fractions F1 and F3 - F7.

S1. ¹H-NMR spectra of the analyzed samples F1 and F3 - F7

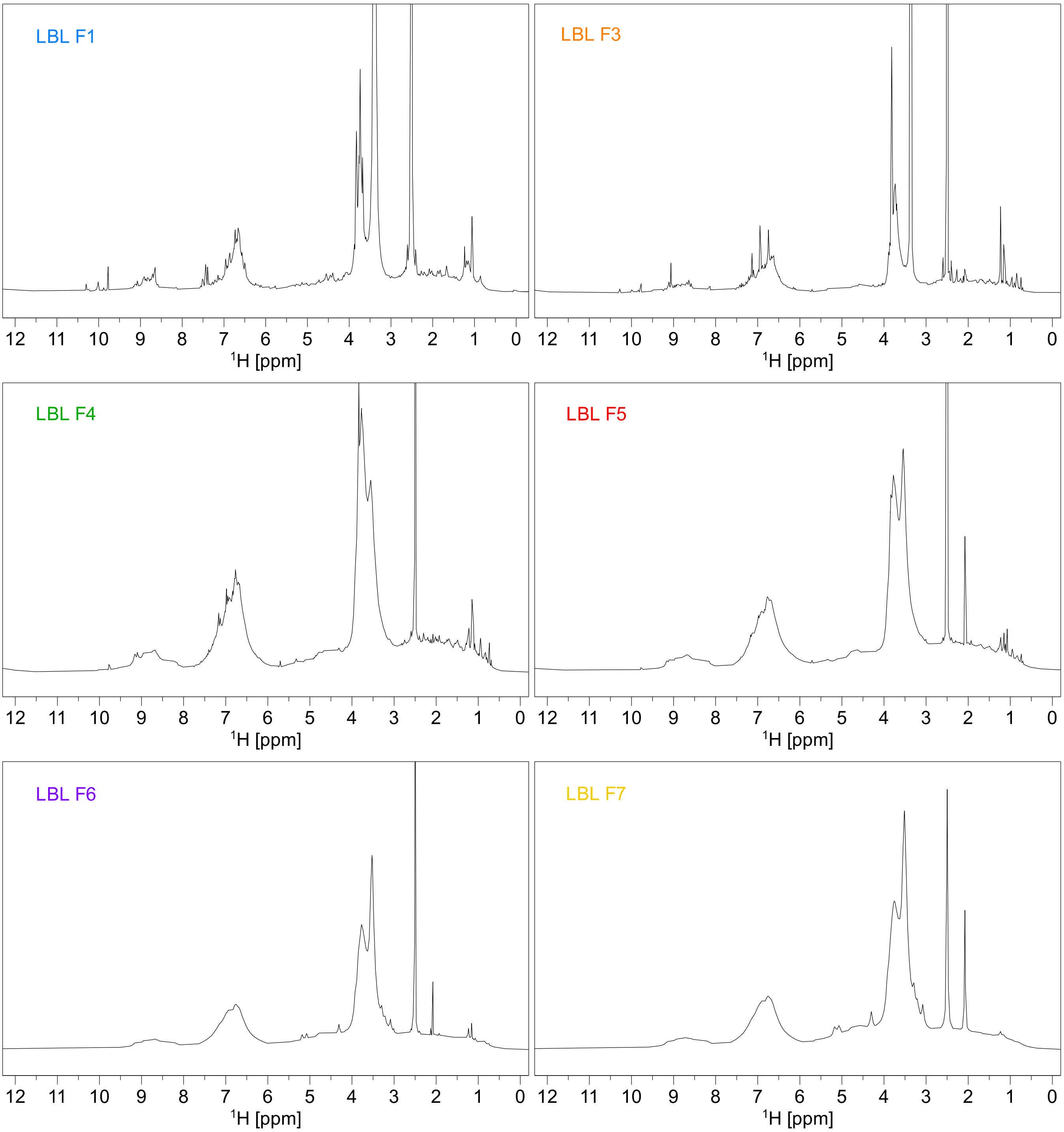

Figure S1. ¹H-NMR spectra of the samples F1 and F3 - F7 in DMSO-d_6, recorded on a 700 MHz spectrometer.

Table S1. Assignment of the characteristic regions in the ^{1}H-NMR spectrum.

δ [ppm]	**Assignment**
12.4	hydroxy groups of carboxylic acids
10.5 - 9.5	aldehyde group (e.g. cinnamyl aldehyde)
9.0 - 8.5	free phenolic hydroxy groups
7.5 - 6.0	aromatic protons
4.0 - 3.4	aliphatic side chain
3.8 - 3.7	alkoxy group
2.5	DMSO
< 2.5	aliphatic components

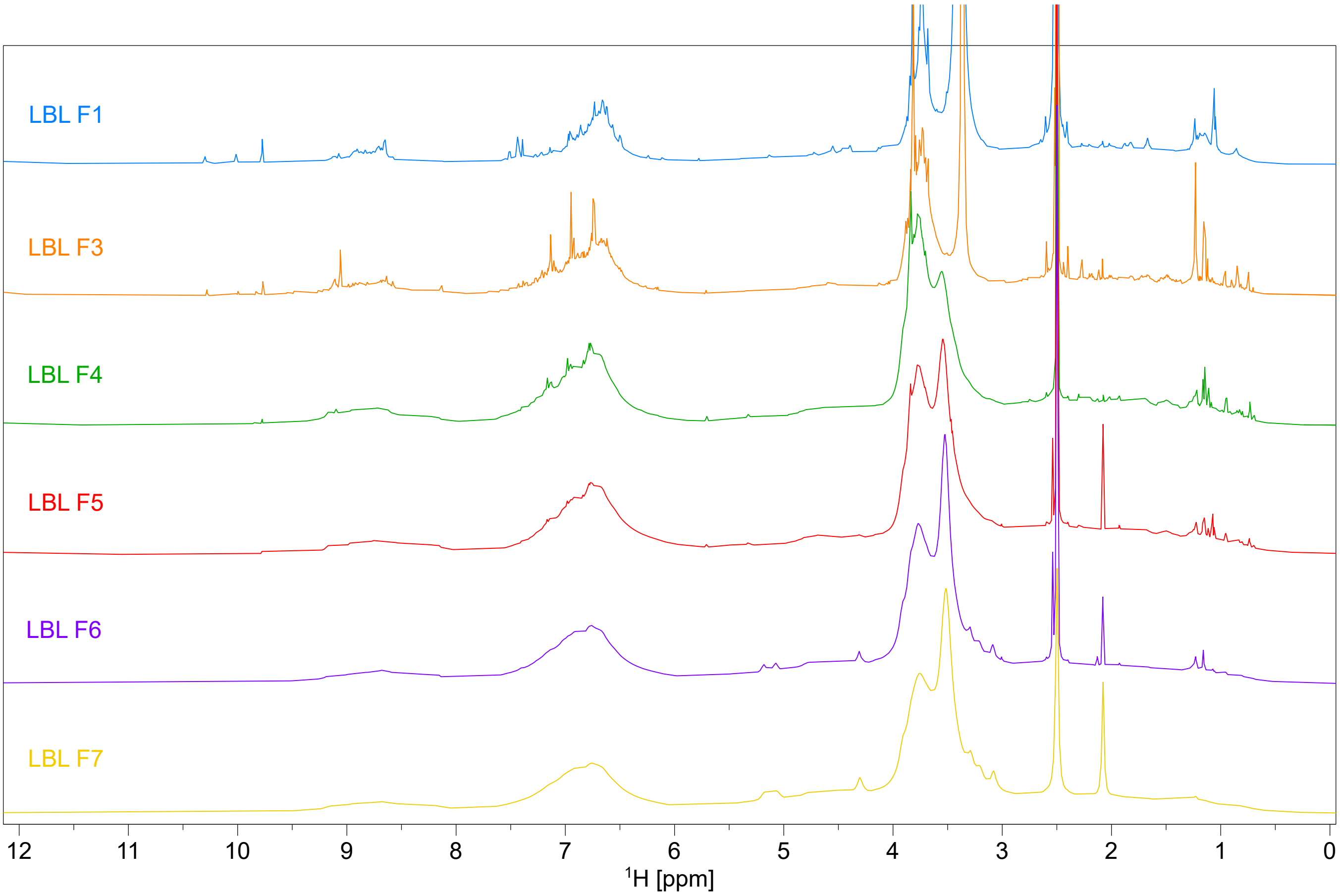

Figure S2. ^{1}H-NMR spectra of F1 and F3 - F7.

^{1}H-NMR spectra were recorded for all samples and stacked to illustrate differences between the samples. F1 is the diluted sample of 10 - 20 µL of the stock solution in deuterated DMSO. This way, a better resolved spectrum could be achieved. F1 and F3 show a better resolution than the other samples. On the one hand, the lignin molecules have better solubility in these fractions. On the other hand, the molecules in these fractions are smaller, which leads to sharper signals. In the latter fractions, parts of the sample are undissolved and due to the larger molecule size, the tumbling is inhibited and the signals get broader. Main differences between all spectra are in the region below 3 ppm and above 8 ppm.

S2. Electron spin resonance (ESR) spectroscopy of all six analyzed samples, dissolved in DMSO-d_6

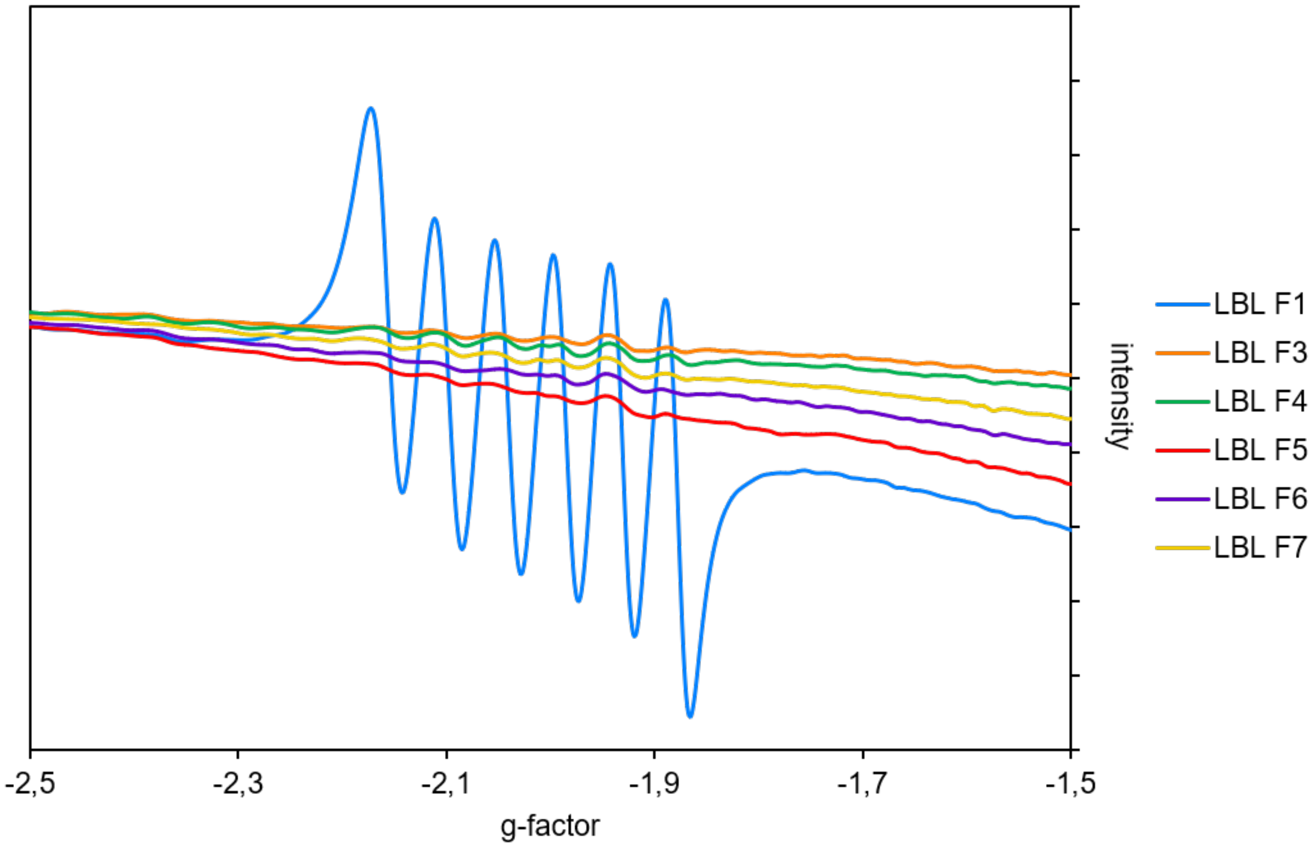

Figure S3. ESR spectra of F1 and F3 - F7.

The ^{1}H-NMR spectrum of F1 showed broad lines, which could be caused by a high number of paramagnetic ions in the sample. To check this, electron spin resonance (ESR) spectroscopy of the solutions in DMSO-d_6 was performed using a benchtop ESR spectrometer MS 5000 by Freiberg Instruments. The tubes were dipped in the solutions, filled and then sealed using plasticine. Afterwards, the tubes were put in a sample holder and inserted in the spectrometer. The microwave frequency was 9.4537 GHz and the magnetic field was changed between 130 to 500 mT. The microwave power was set to 10,000 mW, a modulation frequency of 0.5 mT and a sweep time of 60 s were used. In Figure S3 the ESR spectra of all six samples are presented. Sample F1 shows a sextet with a g factor of 2.0. According to the hyperfine coupling[62], the sextet is caused by Mn(II) ions with a spin I of 5/2. During the extraction process of the Lignoboost Lignin, the first solvent mixture extracted most of the Mn(II) ions, which leads to the high content. The concentration of Mn(II) in F3 - F7 is low. For quantitative ^{13}C-NMR analyses, therefore, the addition of relaxation agent Cr(acac)$_3$ was necessary for F3 - F7 to achieve a complete relaxation of all nuclei.

S3. Quantitative ^{13}C-NMR spectra of the analyzed samples F1 and F3 - F7

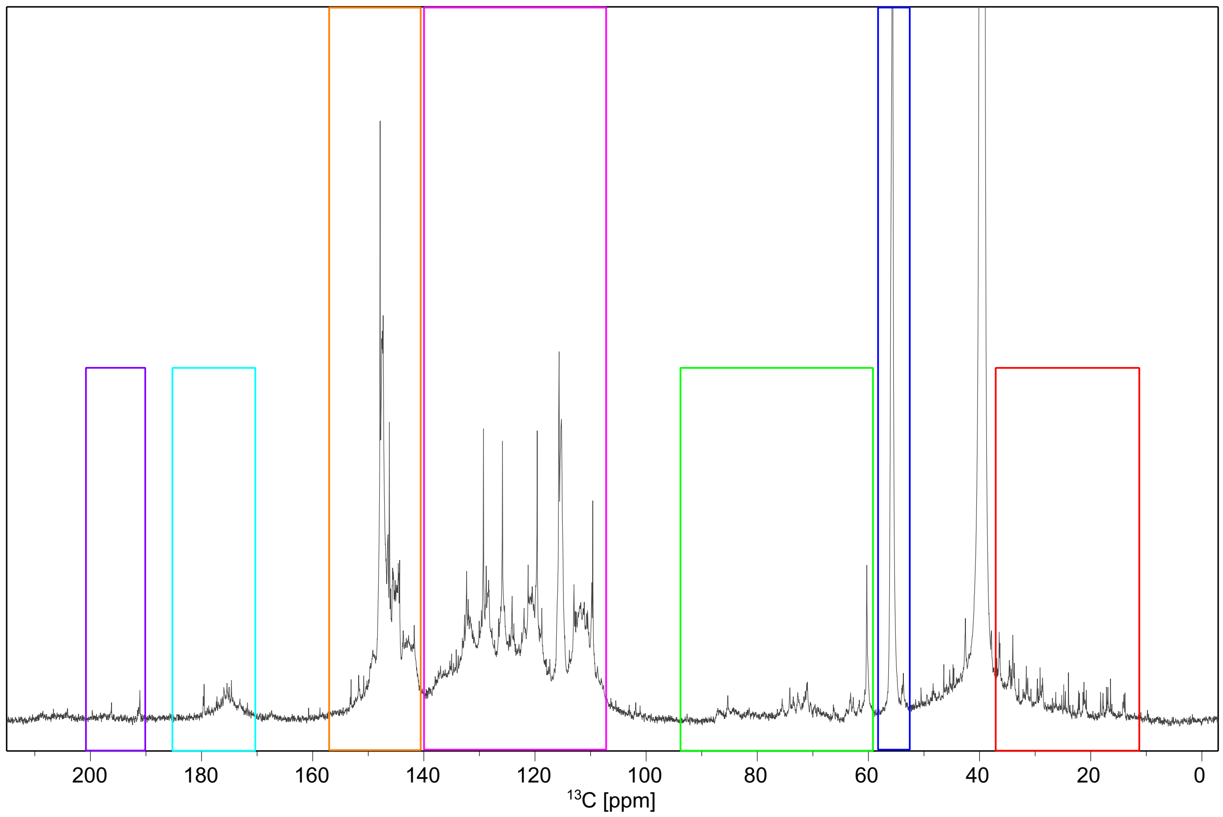

Figure S4. Quantitative ^{13}C-NMR spectrum of F3 with integral regions.

In Figure S4 the characteristic regions of the ^{13}C-NMR spectrum are presented exemplarily for F3. The integrals of these regions are further specified in Table S2. Between 200 to 190 ppm, signals of carbonyl groups, mainly aldehydes as well as ketones, are found (violet). Signals of carboxylic acids and esters are located at lower chemical shift values between 185 to 170 ppm (light blue). In the orange region, 158 to 140 ppm, are the signals of $C_{\text{Ar-O}}$ groups. In the pink region are signals of $C_{\text{Ar-C}}$ and $C_{\text{Ar-H}}$ groups. Between 90 to 60 ppm are signals of ether and hydroxy groups (green), characteristically for the linkages between the monolignols. The intense, sharp signal at 56 ppm can be assigned to methoxy groups (dark blue). Below 54 ppm are signals, characteristically for the C_α and C_β atoms of free G units as well as the C_β of β-5' and β-β' linkages. Additionally, signals of aliphatic compounds can be found in this region (red).

Table S2. Assignment of the most important peaks in Figure S4.

**Color**	δ **[ppm]**	**Assignment**
violet	200 - 190	carbonyls: ketones, aldehydes
light blue	185 - 170	carboxylic acids and esters
orange	158 - 140	$C_{\text{Ar-O}}$
pink	140 - 110	$C_{\text{Ar-C}}$
green	90 - 60	side chain
dark blue	58 - 54	alkoxy groups (mainly methoxy)
red	40 - 10	aliphatic components

In Figure S5, the ^{13}C-NMR spectra of all samples are presented to visualize differences between them. One of the biggest differences are the signals in F7 between 80 to 70 ppm. These signals can be assigned to carbohydrates that are still present in the sample and could possibly not be extracted by the solvent mixtures. Additionally, F1 has the highest intensity of all analyzed fractions for the region between 200 to 190 ppm. This indicates a higher content of aldehydes or ketones in this sample in comparison to F3 - F7. The aromatic region, on the other hand, is quite similar in all six spectra.

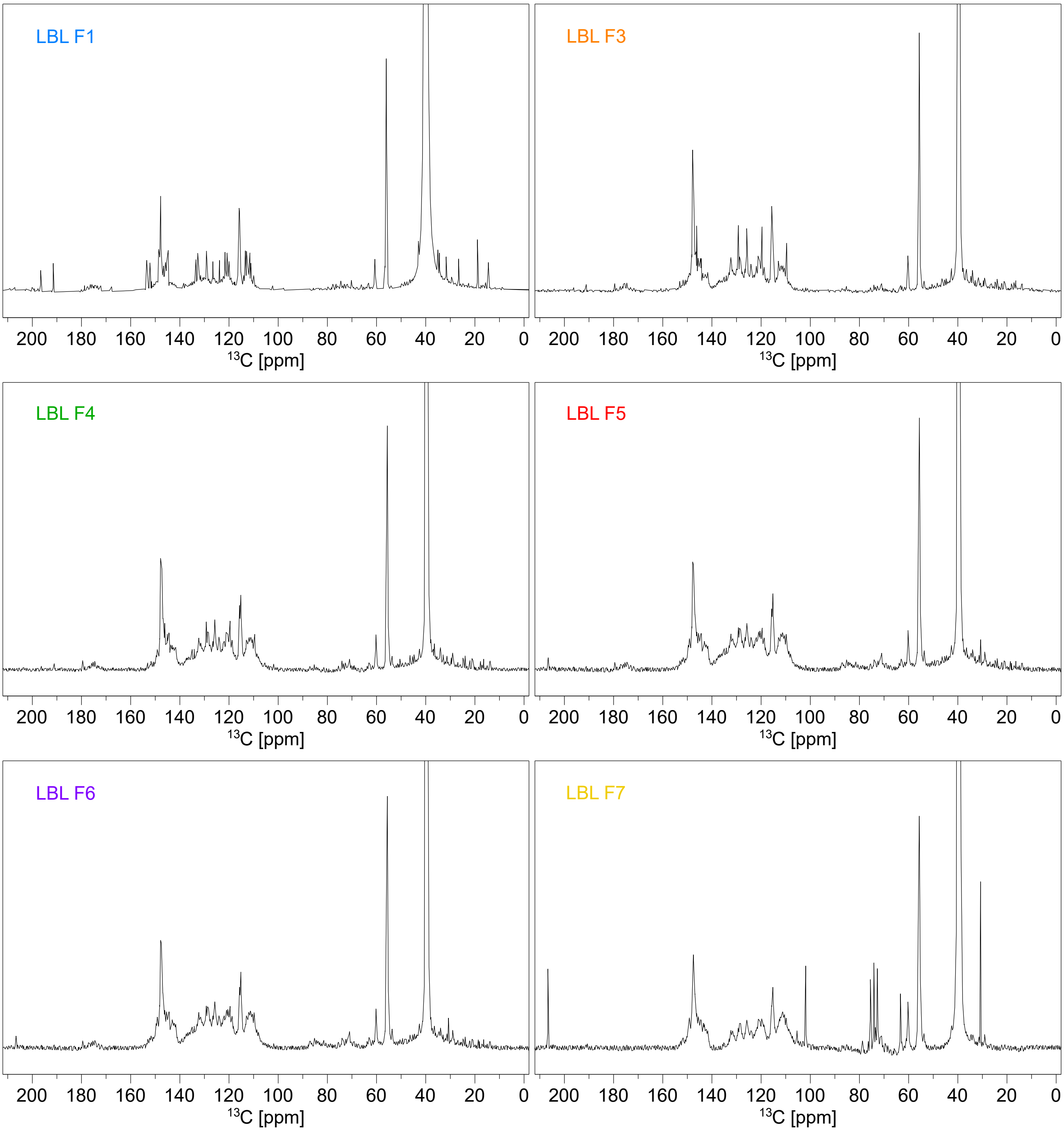

Figure S5. Quantitative ^{13}C-NMR spectra of the samples F1 and F3 - F7 in DMSO-d_6, recorded on a 700 MHz spectrometer.

S4. Quantification of the ^{13}C-NMR spectra of F1 and F3 - F7

The evaluation of the quantitative ^{13}C-NMR spectra, based on the integrals of different structural specific regions, are described in Table S3, using F3 as an example. The most important regions are additionally presented in Table 1 in the main part of this paper. The values of all integrals of the six samples are shown in Table S4. The integrals of region no. 8 ($C_{Ar\text{-}O}$), 12 ($C_{Ar\text{-}C}$) and 13 ($C_{Ar\text{-}H}$) are presented in Figure S6 as bar diagrams to visualize trends between the fractions. With increasing fraction number, the number of $C_{Ar\text{-}H}$ atoms decreases up to F6 whereas the $C_{Ar\text{-}C}$ number increases. As described, position 2 and 6 are always protonated in lignin. However, position 5 can be substituted, e.g. in β-5' linkages. Hence, with increasing fraction number, the number of β-5' linkages is also increasing.

Table S3. Assignments of the structural units and their quantification (in amount per C_9 unit), exemplary for F3, in reference to Capanema et al.[15]

No.	δ [ppm]	Assignment	per C_9 unit
1	210 - 200	nonconjugated CO	0.09
2	200 - 196	α-CO except ferulate	0.03
3	196 - 193	α-CO in ferulate, CO in cinnamyl aldehyde	0.01
4	193 - 191	aldehyde group	0.01
5	182 - 180	C_4 in spirodienone	0.00
6	175 - 168	aliphatic ester	0.08
7	168 - 166	conjugated ester	0.01
**8**	**162 - 142**	**$C_{Ar\text{-}O}$**	**1.78**
9	162 - 160	C_4 in β-1'	0.00
10	157 - 151	$C_3/C_5/C_6$ in 5-5', 5-O-4', cinnamyl aldehyde, spirodienone	0.11
11	144.5 - 142.5	C_3/C_4 in β-5', 5-5', 5-O-4'	0.25
**12**	**142 - 125**	**$C_{Ar\text{-}C}$**	**1.82**
**13**	**125 - 102**	**$C_{Ar\text{-}H}$**	**2.52**
**14**	**90 - 58**	**C_{Alk}-O-R**	**0.84**
**15**	**90 - 77**	**C_{Alk}-O-Ar, C_α-O-Alk**	**0.20**
**16**	**77 - 65**	**C_γ-O-Alk, C-OH_{sec}**	**0.35**
**17**	**65 - 58**	**C-OH_{prim}**	**0.28**
**18**	**58 - 54**	**methoxy group**	**0.93**
19	54 - 52	C_β in β-5', β-β'	0.09
20	35 - 34	C_β in G (free), C_α in β-β'	0.09
21	32.5 - 31.5	C_α in G (free)	0.06

Table S4. Integrals of the selected regions of the quantitative ^{13}C-NMR spectra of all analyzed fractions.

**No.**	**F1**	**F3**	**F4**	**F5**	**F6**	**F7**
1	0.13	0.09	0.10	0.08	0.06	0.11
2	0.06	0.03	0.03	0.03	0.02	0.03
3	0.02	0.01	0.01	0.01	0.01	0.02
4	0.03	0.01	0.01	0.01	0.00	0.01
5	0.00	0.00	0.00	0.01	0.00	0.00
6	0.21	0.08	0.07	0.10	0.07	0.08
7	0.00	0.01	0.00	0.01	0.01	0.01
**8**	**1.76**	**1.78**	**1.72**	**1.86**	**1.93**	**1.71**
9	0.00	0.00	0.00	0.01	0.02	0.01
10	0.09	0.11	0.10	0.21	0.26	0.17
11	0.26	0.25	0.29	0.28	0.27	0.28
**12**	**1.56**	**1.82**	**1.88**	**1.90**	**1.88**	**1.87**
**13**	**2.80**	**2.52**	**2.52**	**2.36**	**2.31**	**2.53**
**14**	**1.38**	**0.84**	**0.80**	**0.96**	**1.21**	**1.52**
**15**	**0.28**	**0.20**	**0.20**	**0.27**	**0.30**	**0.35**
**16**	**0.60**	**0.35**	**0.32**	**0.35**	**0.50**	**0.72**
**17**	**0.49**	**0.28**	**0.28**	**0.33**	**0.40**	**0.46**
**18**	**1.10**	**0.93**	**0.94**	**0.94**	**0.99**	**0.99**
19	0.13	0.09	0.10	0.11	0.11	0.13
20	0.20	0.09	0.09	0.08	0.08	0.08
21	0.10	0.06	0.06	0.06	0.06	0.06

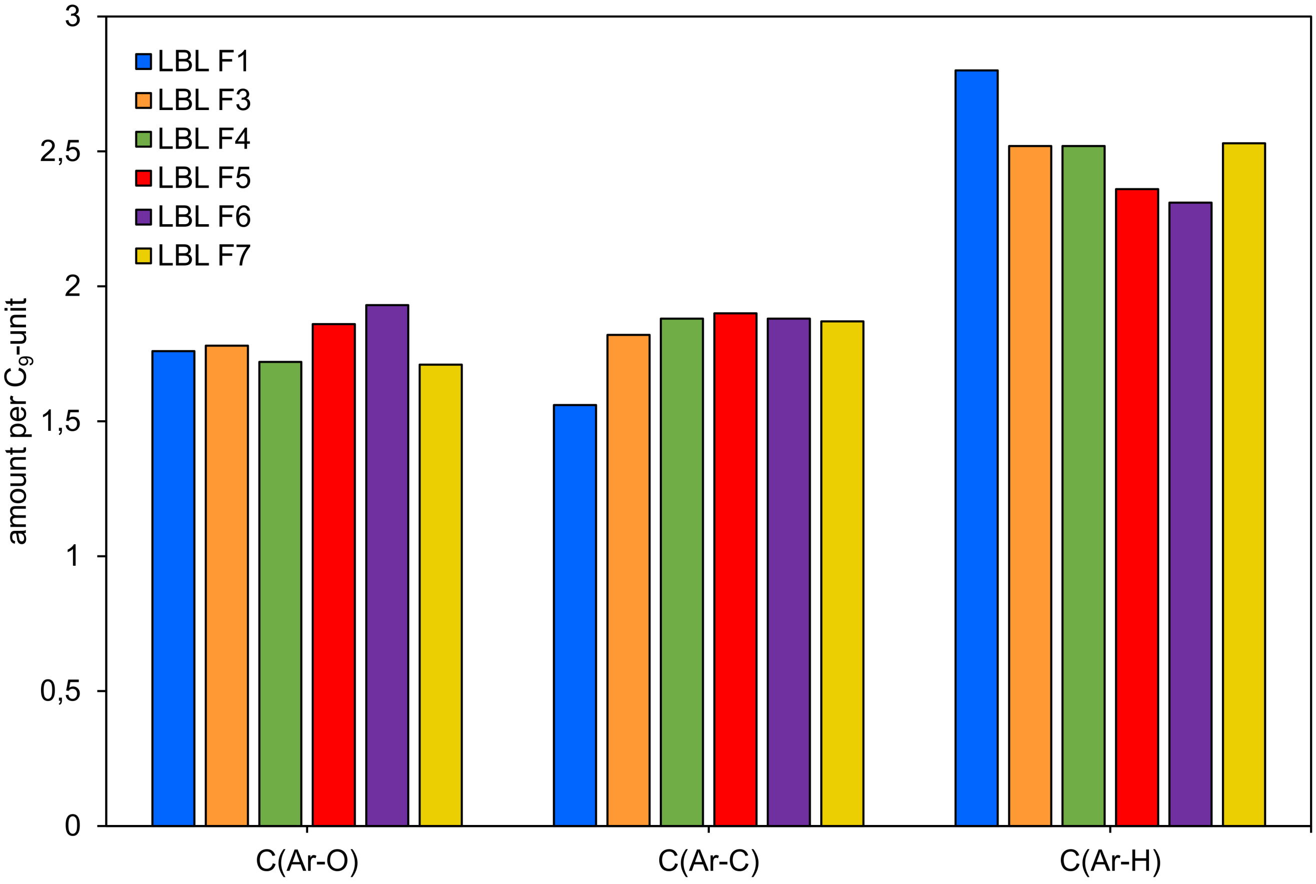

Figure S6. Comparison of the integrals of the aromatic carbons of F1 and F3 - F7.

Additionally, the integrals of different linkages as well as hydroxy and methoxy groups are compared in Figure S7. The integrals of the methoxy groups (no. 18, see Table S4) confirm that the samples originated from soft woods like spruce, because approximately one methoxy group is present in a lignin moiety. The value for F1 is slightly above 1. Either some of the molecules have additional methoxy groups or the signal is overlapping with other components in this sample and hence the methoxy content is overestimated. More interesting trends are visible in integral number 14 (see Table S4), where signals of C_{Alk}-O-R groups are present. F1 has a high content with more than 1.38. However, with increasing fraction number, the number is decreasing until F4 and then increases again with its maximum at F5. This shows that in fraction F1 mainly C_{Alk}-O-R linkages, such as β-O-4', are present and only fewer β-5' linkages. The number of β-O-4' linkages decreases until F4 and increases again. In F7 are β-O-4' and β-5' linkages present, which indicates to larger molecules. Secondary hydroxy groups are either bound on the C_α or C_β position in a lignin molecule, whereas primary hydroxy groups are located at the C_γ atom. Generally, there are more C-OH_{sec} groups present per C_9 unit than C-OH_{prim}.

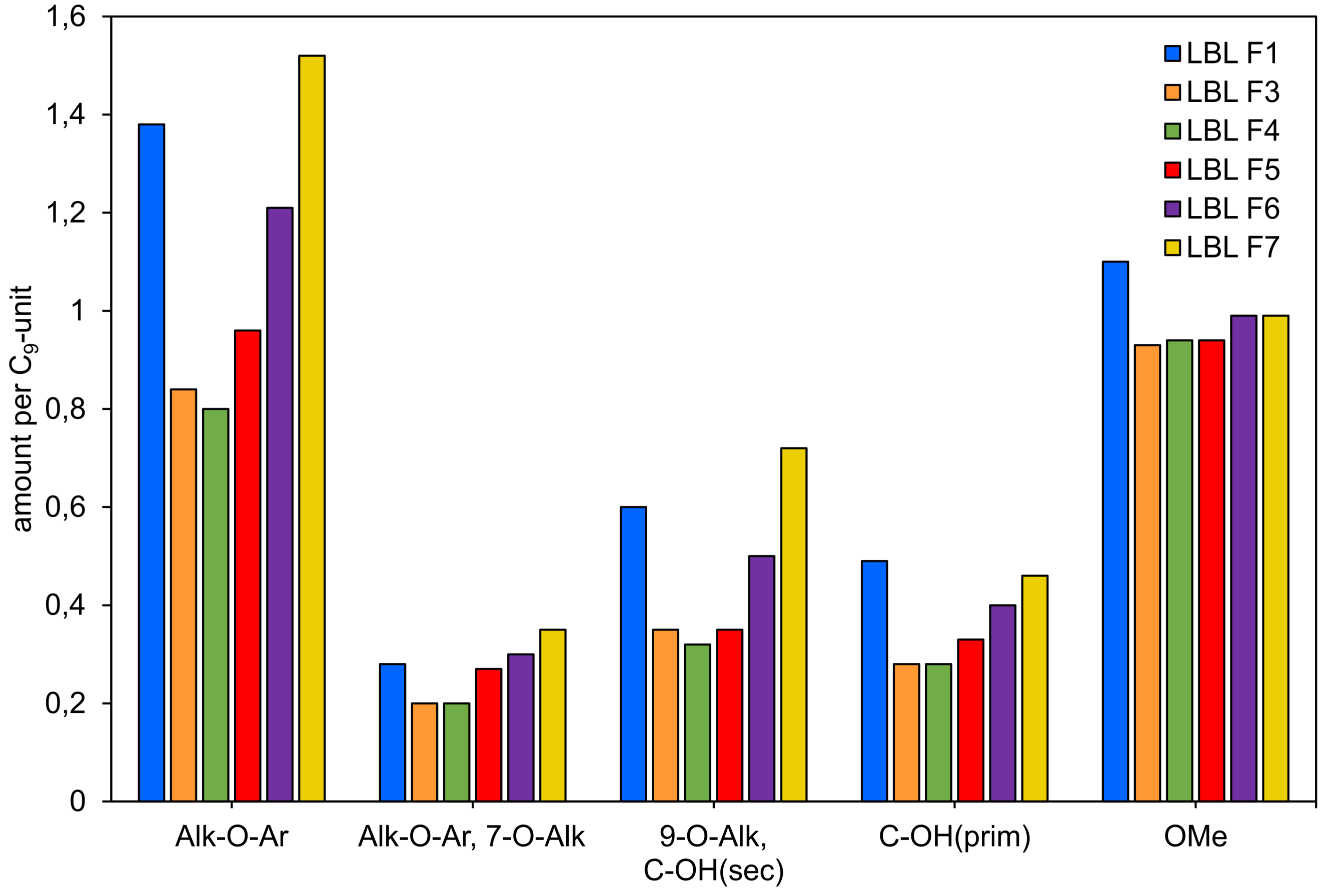

Figure S7. Comparison of the integrals of different linkages as well as different types of oxygen-substituted carbons of F1 and F3 - F7.

To assess the accuracy of these analyses, the standard deviation and coefficient of variance of the integration were calculated. For this purpose, the ^{13}C-NMR spectra of each fraction were processed three times individually using *Topspin version 4.1.3* by Bruker Biospin. An exponential multiplication was applied to the free induction decay (FID) with a line broadening of 5 Hz prior to Fourier Transformation (FT). In a next step, the phase was corrected automatically and adjusted manually, if necessary. The baseline was also adjusted manually. Afterwards, the selected regions were integrated manually following the procedure published by Capanema et al.[15] For the calculation of the standard deviation and the average error, we utilized the eight most important regions, which were also evaluated in the main part of this paper (regions no. 8 and 12 - 18, see Table 1 and Table S3). Especially regions with low integral values showed high errors, because mainly signals at the noise level were integrated.
The calculated average integration errors, which were obtained by this procedure, are presented in Table S5.

Table S5. Average integration errors for the quantitative ^{13}C-NMR analyses of all LBL fractions, in reference to Capanema et al.[15]

LBL fraction	Average integration error [%]
F1	2.92
F3	2.97
F4	3.14
F5	1.19
F6	1.73
F7	5.47
**Mean**	**2.90**

According to our calculations, an average error of 1.19 % (F5) up to 5.47 % (F7) could be determined, which is good alignment with the values reported by Capanema et al. (approximately 2 %)[15].

S5. ^{1}H,^{13}C-HSQC-NMR spectra of the analyzed samples F1 and F3 - F7

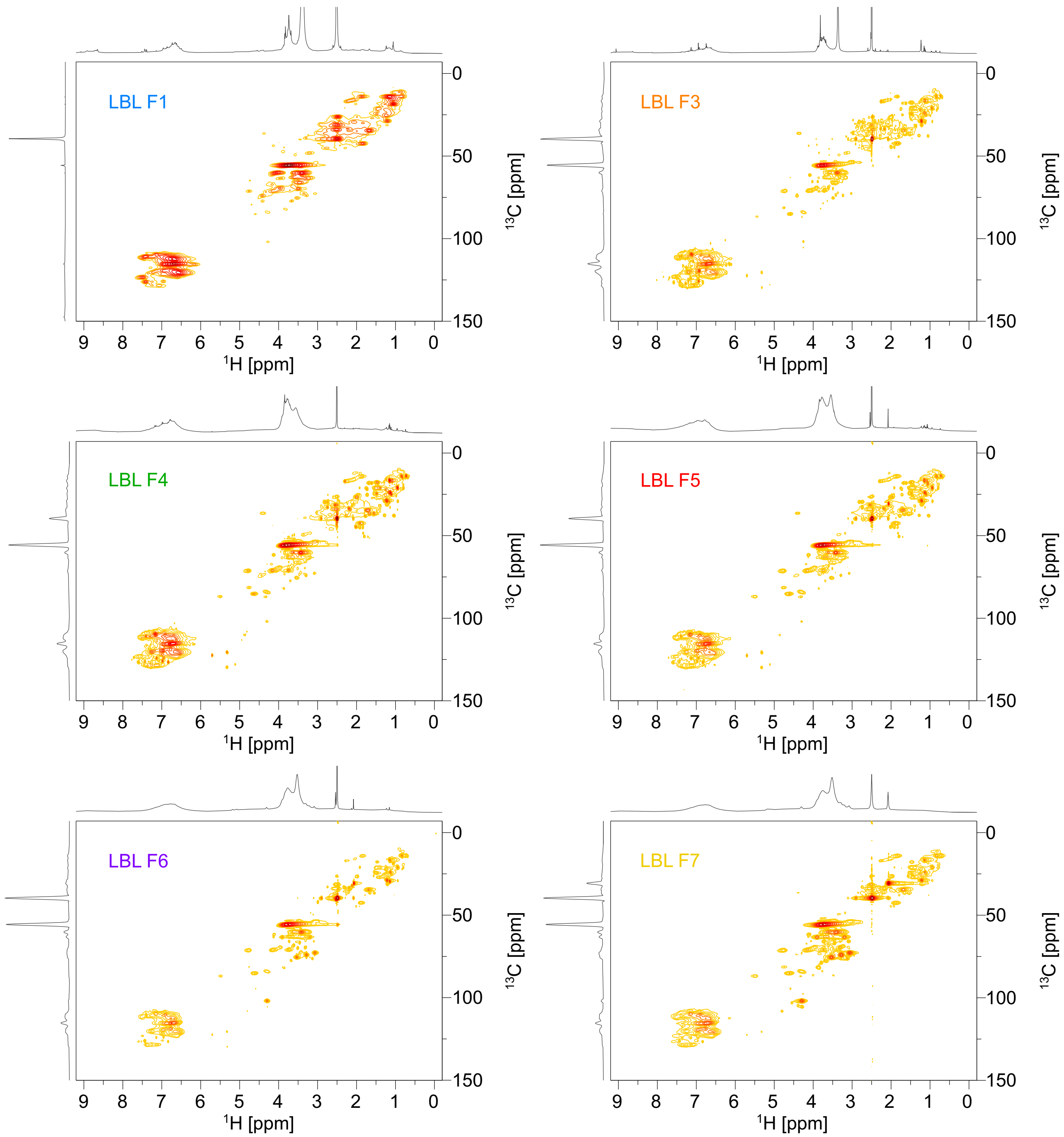

Figure S8. ^{1}H,^{13}C-HSQC-NMR spectra of all samples F1 and F3 - F7, recorded on a 700 MHz spectrometer.

If the HSQC spectra (see Figure S8) are compared, all of them look similar to each other. In the aromatic regions, the signals of the G units can be distinguished. Signals of S units, characteristically for hard woods, or H units (grasses) can not be identified. The signal of the methoxy groups is the most intense signal (except of the signal of the solvent DMSO). In F6 and F7 the signals of carbohydrate impurities are more prominent, as indicated by the three signals at δ_C/δ_H 75 ppm/3.5 - 3.0 ppm or the signal at δ_C/δ_H 101 ppm/4.3 ppm. Due to the extraction, the polysaccharides possibly are not dissolved and hence enriched in the latter fractions or lignin-carbohydrate-complexes (LCC) are present that can not be extracted with the utilized solvent mixtures. In all samples, aliphatic compounds in a region of δ_C/δ_H 45 - 10 ppm/2.5 - 0.8 ppm are observable. An assignment of the different signals is presented in Table S6.

Table S6. Assignments of the signals of the HSQC-NMR spectrum of F3.

**Label**	**Chemical shift δ_C/δ_H [ppm]**	**Assignment**
G'_6	123.3/7.50	C_6-H_6 in oxidized G (C_α = O)
G_6	119.4/6.93	C_6-H_6 in G
G_5	115.4/6.74	C_5-H_5 in G
G'_2	111.0/7.43	C_2-H_2 in oxidized G (C_α = O)
G_2	109.7/7.13	C_2-H_2 in G
C_α	86.7/5.45	C_α-H_α β-5'
B_α	85.1/4.59	C_α-H_α β-β'
A_β	84.0/4.26	C_β-H_β β-O-4'
	75.4/3.50	carbohydrates
	74.0/4.42	carbohydrates
A_α	71.0/4.73	C_α-H_α β-O-4'
B_γ	70.8/4.12	C_γ-H_γ β-β'
B_γ	70.7/3.72	C_γ-H_γ β-β'
C_γ	62.8/3.59	C_γ-H_γ β-5'
	62.6/3.33	carbohydrates
	61.5/4.10	C_γ-H_γ cinnamyl aldehydes
A_γ	60.1/3.39	C_γ-H_γ β-O-4'
A_γ	59.5/3.58	C_γ-H_γ β-O-4'
OMe	55.5/3.73	$-OCH_3$
C_β	53.2/3.43	C_β-H_β β-5'
B_β	53.6/3.03	C_β-H_β β-β'

S6. Optimization of repetition time D1 and contact time P15 for ^{13}C-CP/MAS-NMR

To achieve high signal intensities as well as a short analysis time, the repetition time D1 and contact time P15 needed to be optimized. Here, we used sample F3. At first, the contact time P15 was set to 1.0 ms and the repetition time D1 was varied between 1.0 s to 5.0 s (see Figure S9). The aim was to obtain a high intensity but a delay time as short as possible to reduce the analysis time. Between 3.0 s, 4.0 s and 5.0 s the differences between the signal intensities were not significant, so we decided to apply a delay time of 3.0 s. For the optimization of the contact time, the delay was set to 3.0 s and P15 was varied between 0.2 ms to 10 ms (see Figure S10). The contact time is depending on the CP rate as well as the relaxation time and influences the signal intensities. Our aim was to achieve high intensities as well as a good reproducibility of the signal intensities in the singe pulse (SP)/MAS-NMR spectrum. The acquisition of the SP-NMR spectrum is described in section S7. A contact time of 7.0 ms showed the highest similarities between the CP and SP spectra, as well as high intensities. Hence, for all further analyses, a contact time of 7.0 ms was used.

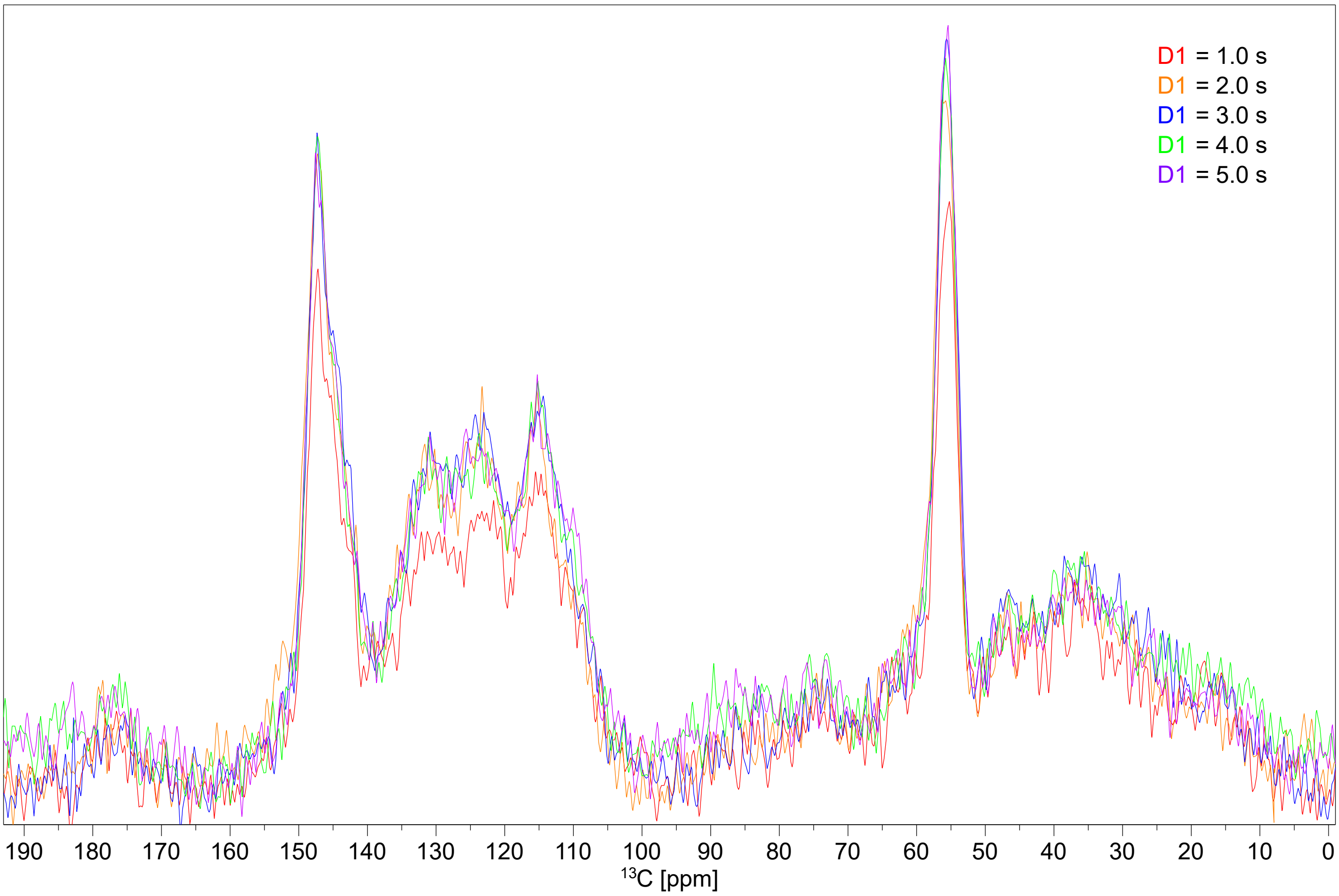

Figure S9. Optimization of D1 for F3. Spectra are scaled to the intensity of the MeO group (55.6 ppm).

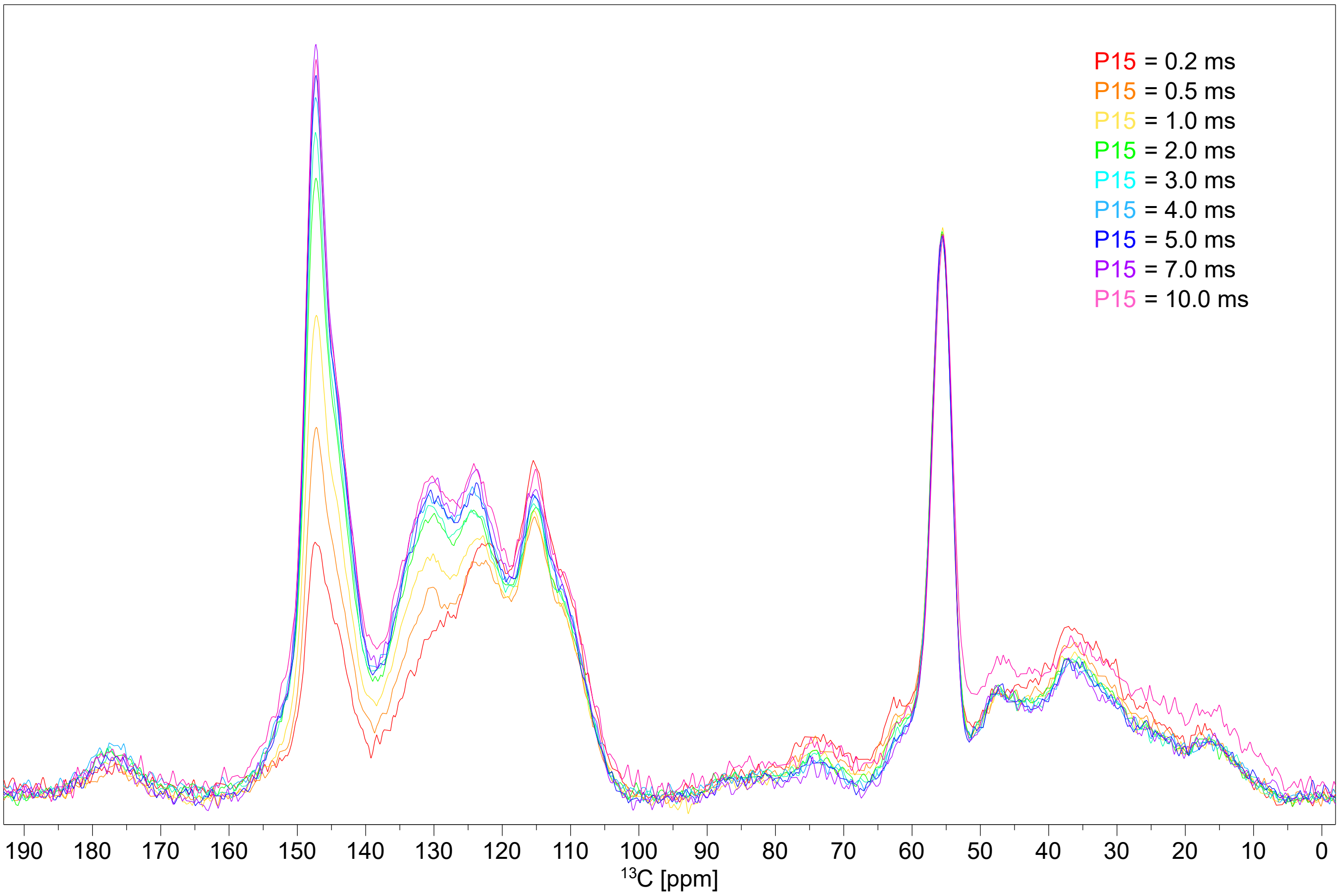

Figure S10. Optimization of P15 for F3. Spectra are scaled to the intensity of the MeO group (55.6 ppm).

S7. ^{13}C-SP/MAS-NMR of sample F3

Using a single pulse excitation leads to the detection of ^{13}C atoms in the sample as well as the carbon of the probe (mainly PTFE). Therefore, a spectrum of an empty rotor was subtracted from the spectrum of the sample to achieve a corrected spectrum (see Figure S11). Because the carbons are directly excited, longer relaxation times are necessary. Here, 300 s were used to provide a complete relaxation. However, instead of long analysis times, this results in a worse signal-to-noise ratio in comparison to the CP/MAS spectra. Nonetheless, the signals can be quantified.

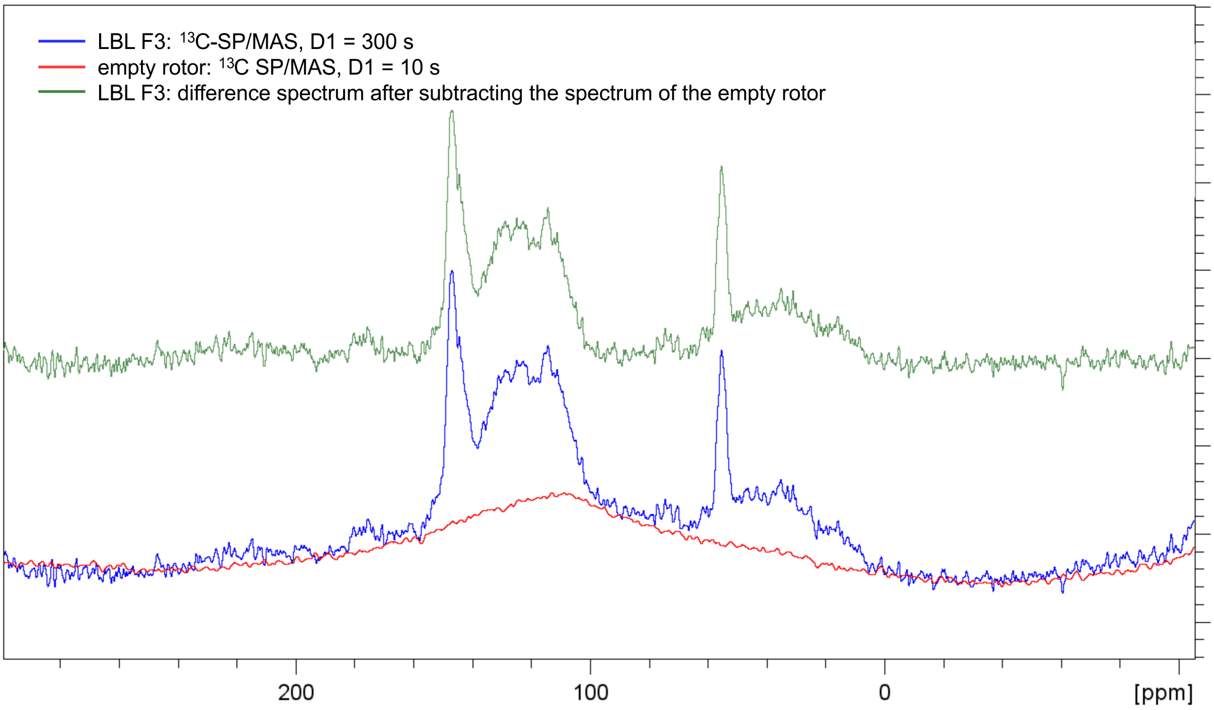

Figure S11. ^{13}C-SP/MAS-NMR spectra of F3 (blue), an empty zirconium dioxide rotor with a PTFE cap (red) and the difference spectrum after subtracting the red spectrum. Spectra were recorded on a 700 MHz spectrometer and the image was created using *Topspin (version 4.1.3)*.

S8. ^{13}C-CP/MAS-NMR of the analyzed samples F1 and F3 - F7

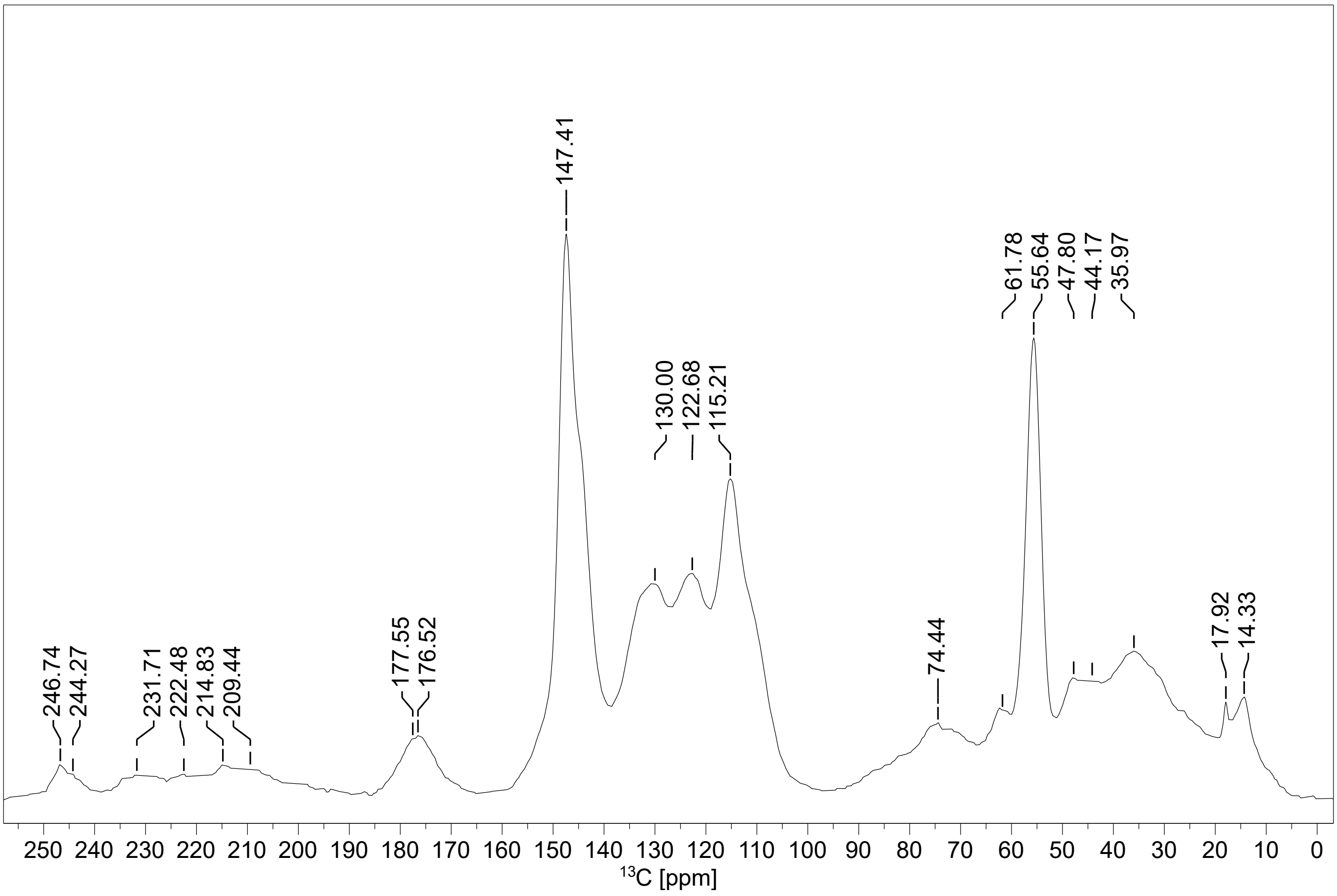

Figure S12. ^{13}C-CP/NMR spectrum of F1 with the chemical shifts of the most important peaks.

Table S7. Assignments of the signals of the solid-state ^{13}C-NMR spectrum.[66,67]

δ [ppm]	Assignment
250 - 210	spinning side bands of the aromatic region
170	carbonyls: carboxylic acids, esters
147	C_3 in G (β-O-4', 5-5', β-β', 1-CHO)
130	C_α, C_β in cinnamyl alcohols and -aldehydes
123	C_1 and C_6 in G
115	C_5 (β-O-4', β-β') and C_2 (with -COOH in C_1) in G
74	C_β in β-O-4'
62	C_γ in β-O-4' with α-OH
56	alkoxy groups, mainly methoxy
< 50	spinning side bands of aromatics, aliphatic components

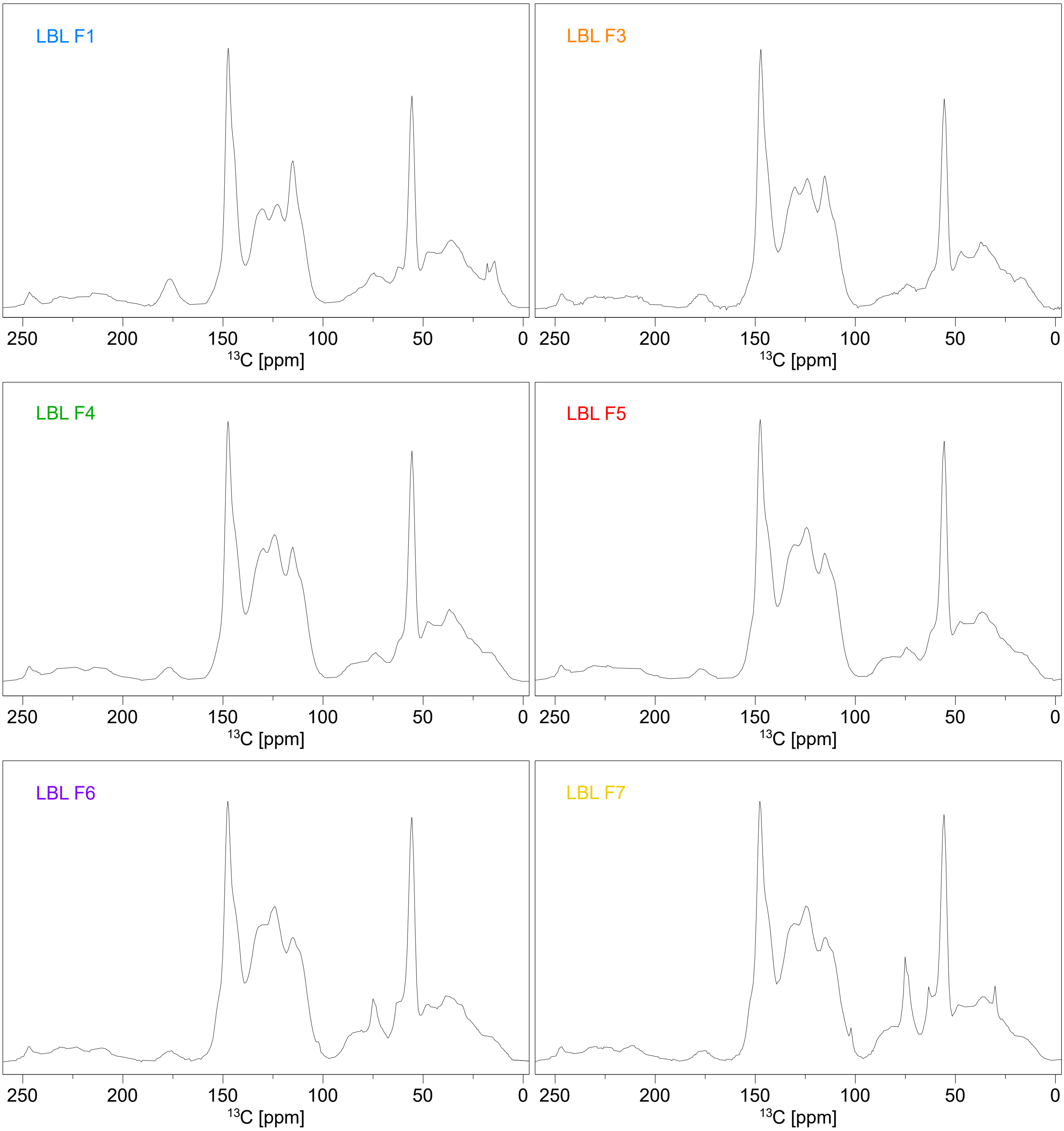

Figure S13. ^{13}C-CP/MAS-NMR spectra of the analyzed samples F1 and F3 - F7, D1 = 3.0 s, P15 = 7.0 ms, recorded on a 400 MHz spectrometer.

S9. ^{1}H,^{13}C-HETCOR-NMR spectroscopy of sample F3

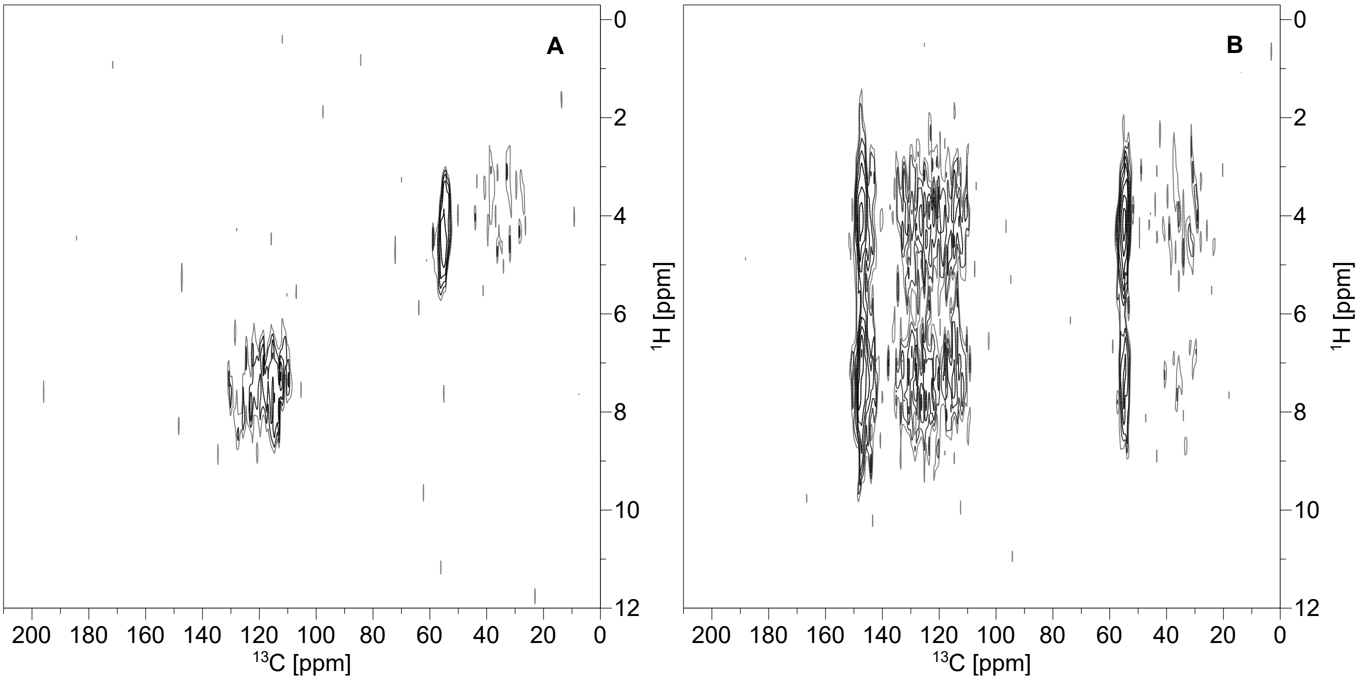

Figure S14. ^{1}H,^{13}C-HETCOR-NMR spectra of F3, A) contact time 200 µs, B) contact time 3.0 ms.

Depending on the contact time in a ^{1}H,^{13}C-HETCOR-NMR analysis, correlations between spatial adjacent nuclei can be depicted. Here, we used a contact time of 200 µs (see Figure S14, A) and 3.0 ms (see Figure S14, B). In the left spectrum (A), three regions can be distinguished: δ_C/δ_H 125 - 105 ppm/8 - 5 ppm (aromatic region, $C_{Ar\text{-}H}$), δ_C/δ_H 56 ppm/3 ppm (methoxy group), δ_C/δ_H 50 - 30 ppm/3 - 1 ppm (aliphatic region). In the aromatic region, signals with higher chemical shifts than 130 ppm are not visible, because these carbon atoms are not protonated ($C_{Ar\text{-}C}$ and $C_{Ar\text{-}O}$). Using low contact times, only correlations within the groups are present, e.g. the methoxy-C only correlates with methoxy-H nuclei. By increasing the contact time to 3.0 ms, more signals appear. The aromatic signals are correlating additionally with the methoxy groups and the side chain and vice versa. Between 50 to 30 ppm, only a few additional correlations are visible, mainly with aromatic protons. The distance between both groups is probably too large to correlate. Furthermore, signals between 150 to 140 ppm are visible. $C_{Ar\text{-}C}$ and $C_{Ar\text{-}O}$ are correlating with $C_{Ar\text{-}H}$ as well as methoxy groups and the aliphatic side chains. The ^{1}H,^{13}C-HETCOR-NMR spectrum with longer contact time shows aromatic and aliphatic functionalities, which are spatially adjacent (range < 6 Å). This proves that the monolignols are linked, networks are formed and single structural elements are not isolated, which is typical for lignin.

S10. ESI-MS and GALDI-MS mass spectra of the six analyzed LBL fractions

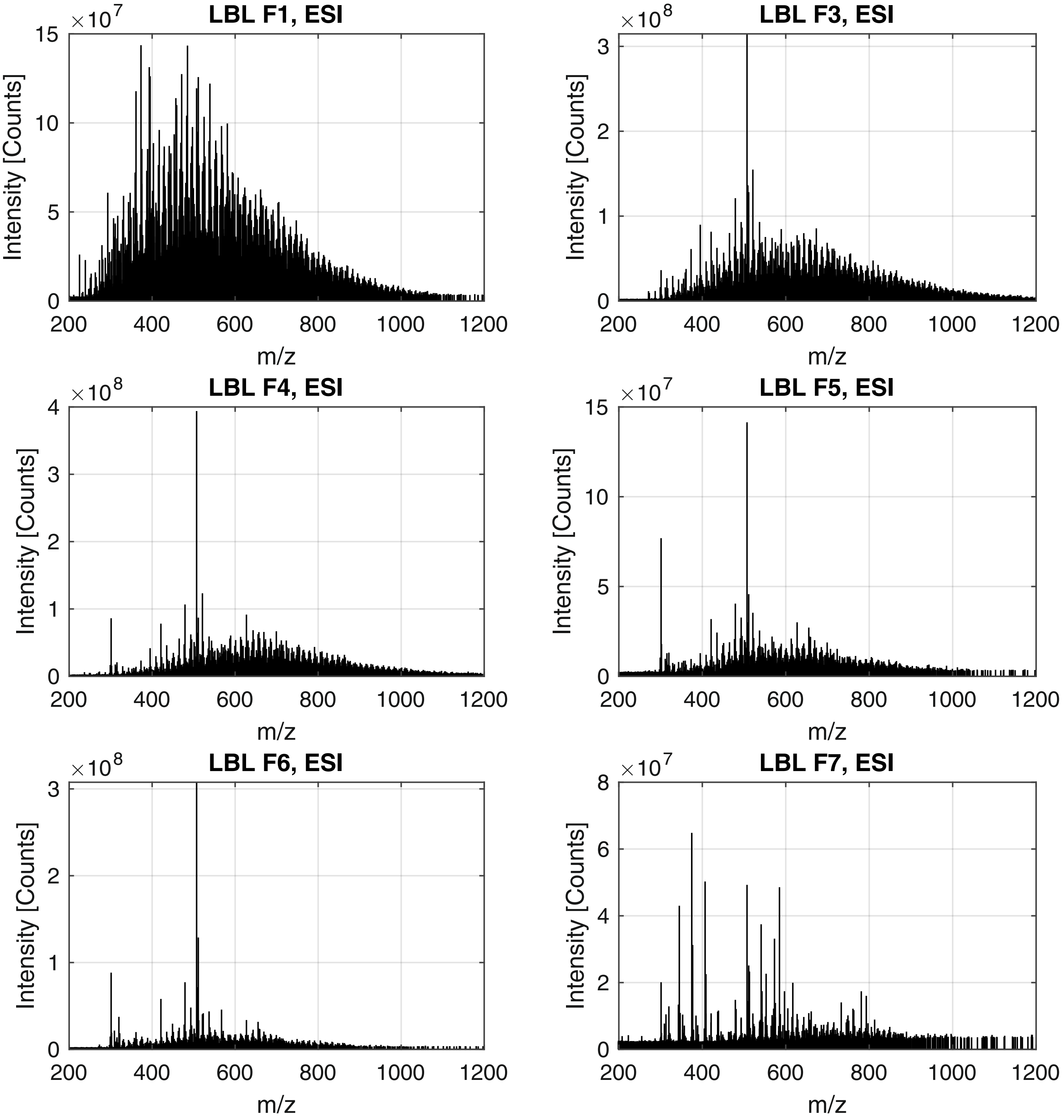

Figure S15. ESI(−)-FT-ICR-MS mass spectra of fractions F1 and F3 - F7.

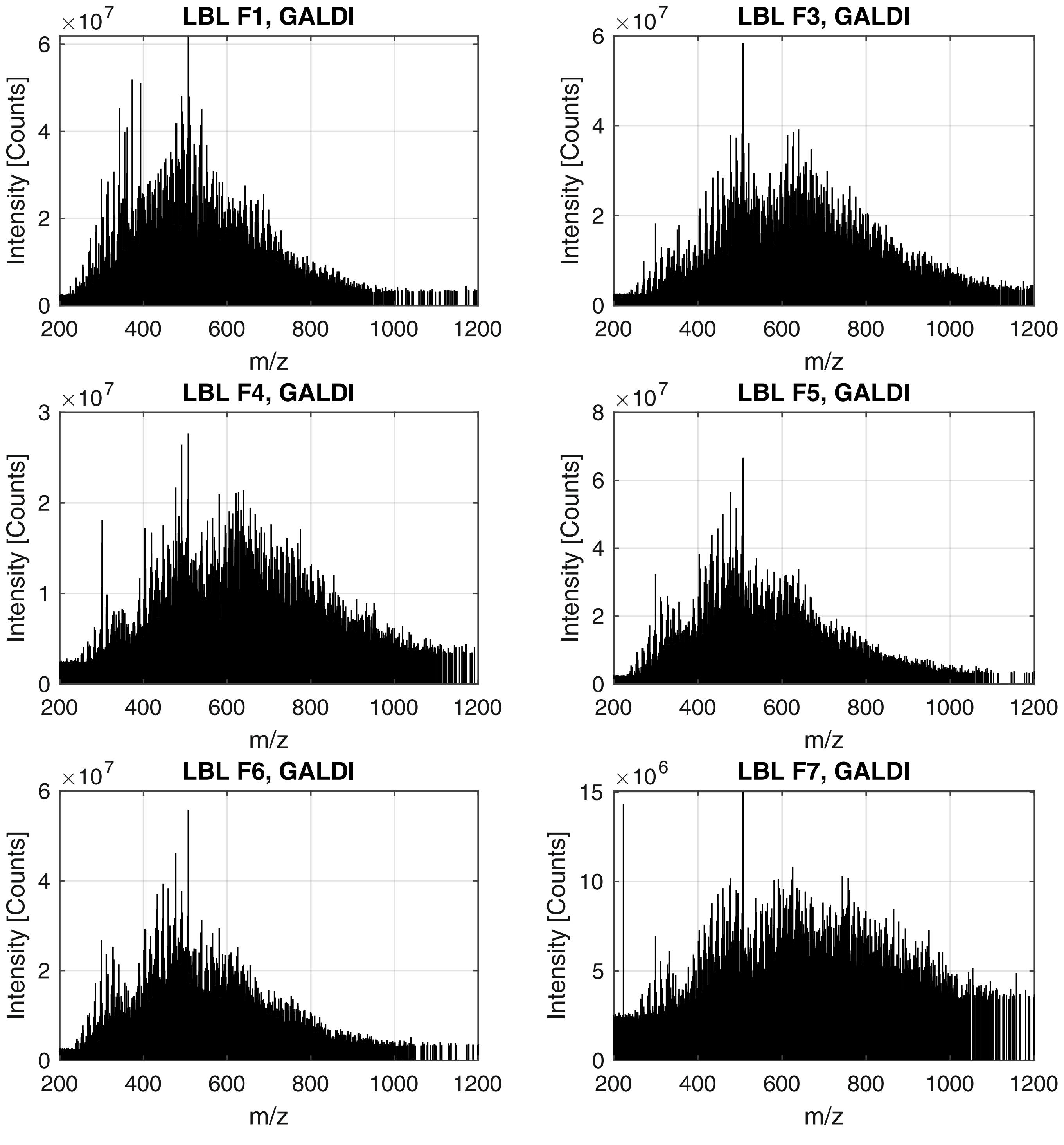

Figure S16. GALDI(−)-FT-ICR-MS mass spectra of fractions F1 and F3 - F7.

As already discussed in the main part of the paper, highest peak and median m/z values are obtained for fractions F1 - F4 by using ESI-MS. In contrast, GALDI-MS seems to be more convenient to ionize analytes from fractions F5 - F7, as the higher peak numbers and median m/z values indicate. Hence, with increasing fraction number (i.e., amount of acetone in the solvent mixture), the molecular size of the extracted lignin molecules seems to increase, whereas the polarity and solubility of these compounds decrease.
The higher sensitivity of ESI(−)-FT-ICR-MS in comparison to GALDI(−)-FT-ICR-MS can be comprehended by comparing the overall ion intensities. For ESI-MS analyses, the maximum intensity varies between $6 \cdot 10^7$ counts and $4 \cdot 10^8$ counts. The maximum intensities for the GALDI-MS analyses are roughly one magnitude below these values ($1.5 \cdot 10^7$ - $7 \cdot 10^7$ counts).

S11. Comparison of relative abundancies for oxygen-containing and sulfur-/oxygen-containing molecular formula classes to the FT-ICR-MS data sets of the six different LBL fractions

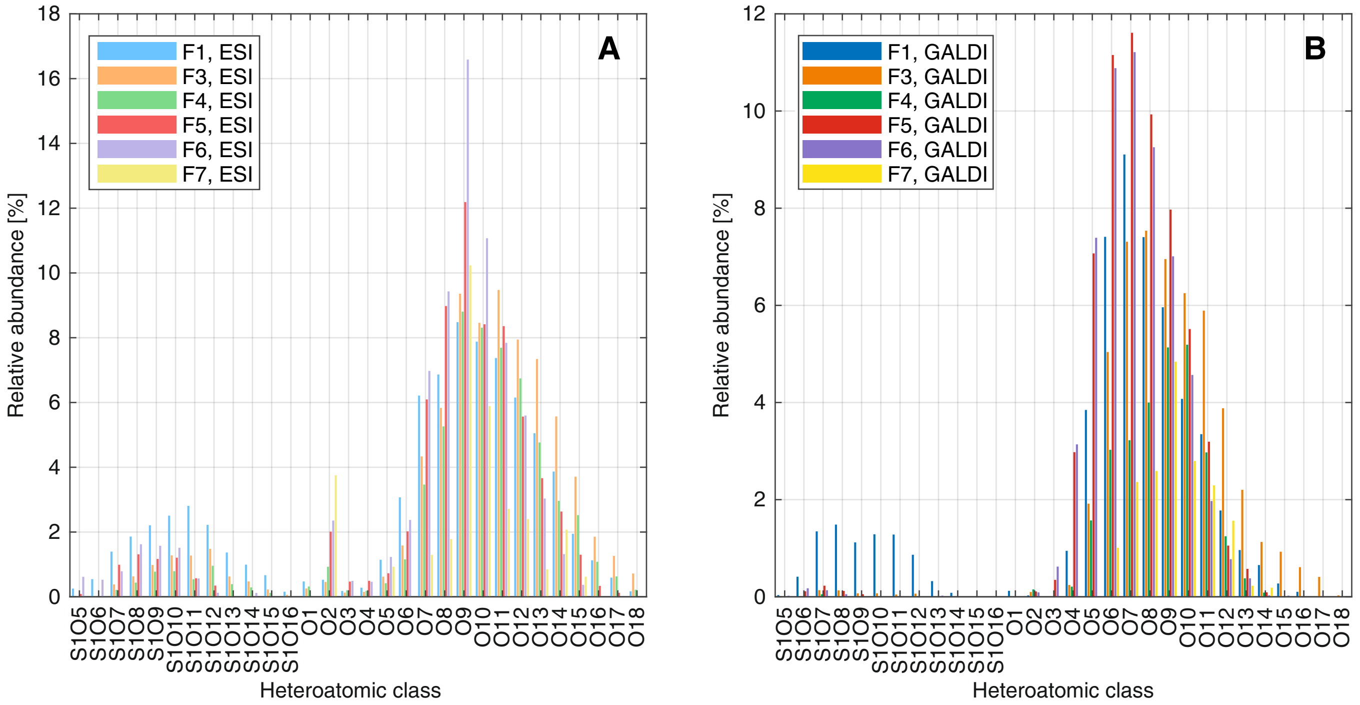

Figure S17. Comparison of relative abundancies of molecular formula classes S_1O_5 - S_1O_{16} and O_1 - O_{18} assigned to the FT-ICR-MS data sets of the six fractions. A - ESI, B - GALDI.

Figure S17 illustrates the relative abundancies for compound classes S_1O_5 - S_1O_{16} and O_1 - O_{18} for all six fractions that were analyzed by ESI(−)- and GALDI(−)-FT-ICR-MS. The general trends for the different heteroatomic classes follow a comparable trend as the total numbers of assigned molecular formulae (see Figure 6 in the main part of the paper). Fractions F1 and F3 seem to possess considerable amounts of molecules, which belong to the illustrated compound classes. Relative abundancies for oxygen-containing compound classes are higher than for sulfur-oxygen-containing classes. Thus, we can assume that the relative amount of sulfate- or sulfonate-containing compounds in these samples is smaller than the amount of oxygen-containing lignin molecules. For samples F4 - F7 the highest relative abundancies are found for medium oxygen values ($3 \leq o \leq 15$). Sulfur-oxygen-containing molecules are only assumable in small quantities for these fractions. Most likely, these compounds possess a high polarity and therefore solubility, which leads to a separation of these molecules in the first two or three fractions (F1 - F3).

S12. n_C-*DBE* plots of oxygen- and sulfur-/oxygen-containing compound classes for all six LBL fractions

Figures S18 and S19 illustrate the n_C-DBE plots for oxygen- and sulfur-/oxygen-containing compound classes O_7 - O_{10} and S_1O_9 - S_1O_{12} for all six fractions, analyzed by ESI- and GALDI-FT-ICR-MS.
With increasing fraction number (i.e., amount of acetone in the solvent mixture), an increase of n_C and DBE values for one heteroatomic class can be observed, especially if the GALDI-MS data sets are considered. For ESI-MS data sets, this trend is only obvious for samples F1 – F5. The low solubility of the larger compounds, which are contained in fractions F6 and F7, results in a notably lower number of data points for these samples in the ESI(−)-FT-ICR-MS data sets. This is especially noticeable for sample F7, as clusters of data points are only observable for the GALDI(−)-FT-ICR-MS data sets. In general, according to the plots presented in Figure S18, lignin oligomer sizes from dimers up to pentamers are possible.
Considerably lower numbers of assigned molecules are observed for sulfur- and oxygen-containing compound classes S_1O_9 - S_1O_{12} for all six samples. Nearly all of these compounds are exclusively detectable by ESI-MS. This circumstance can be contributed to a high polarity and thus solubility of these compounds. Most likely, these compounds only have a low concentration, as they are only detected by the more sensitive ESI-FT-ICR-MS. For fraction F7 no molecular ions were detected either by using ESI- or GALDI-MS for all four visualized heteroatomic classes. Maximum n_C values of 47 and DBE values of 26 are observable according to the n_C-DBE plot of class S_1O_{12} for sample F5 (ESI-MS). Thus, lignin oligomers that contain up to four monolignol units with at least one sulfur-containing functional group (e.g., sulfate or sulfonate) are potential structures.

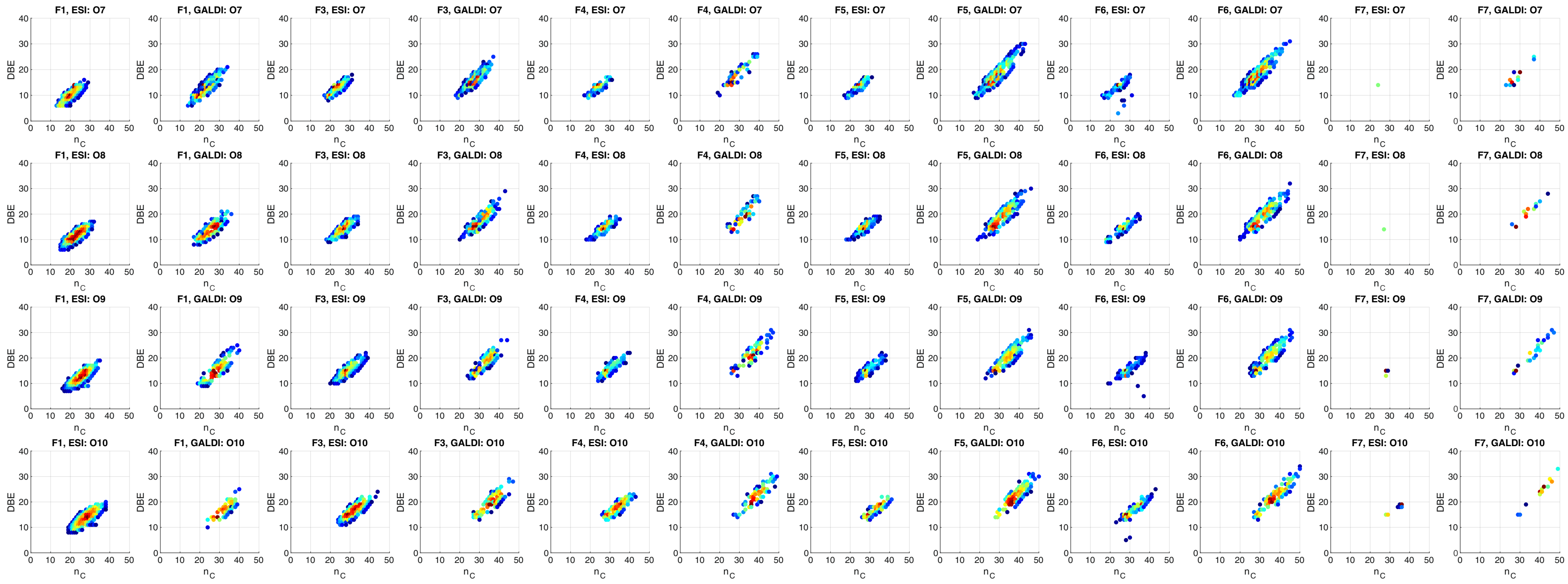

Figure S18. n_C-DBE plots of compound classes O_7 - O_{10} for all six fractions, which were analyzed by ESI- and GALDI-FT-ICR-MS. The observed intensity is presented logarithmic and color-coded (blue: low intensity, yellow: medium intensity, red: high intensity).

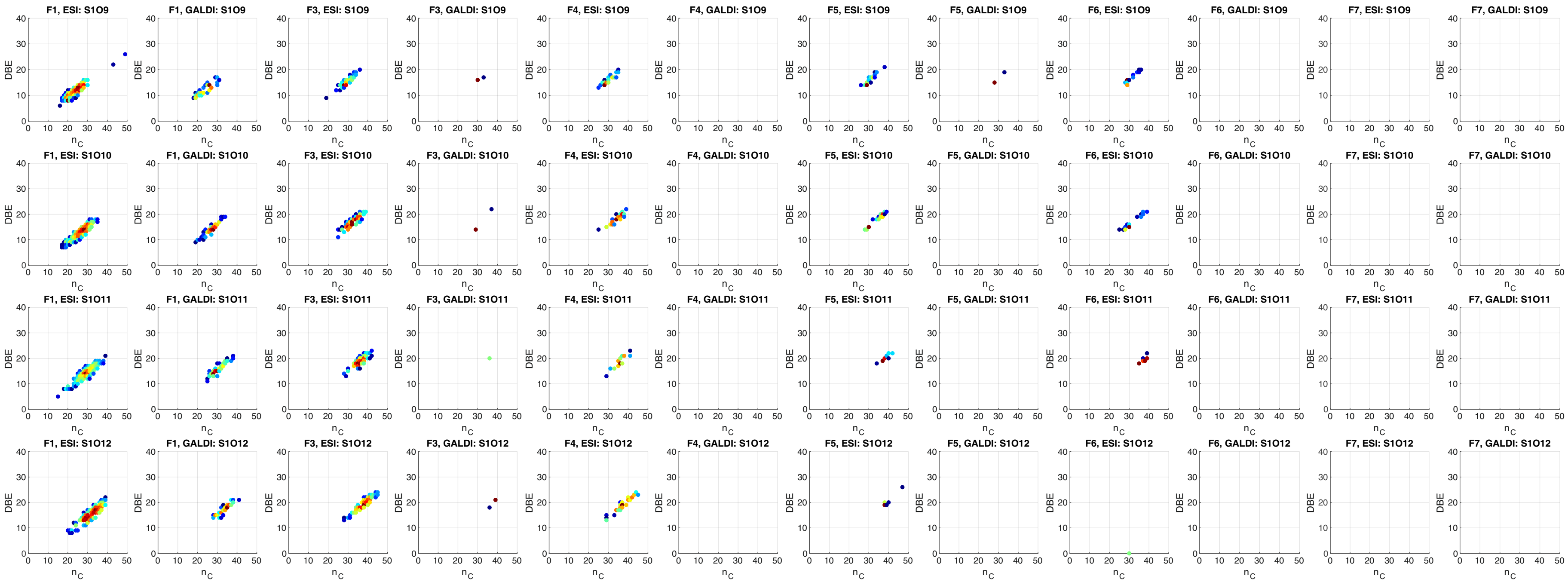

Figure S19. n_C-DBE plots of compound classes S_1O_9 - S_1O_{12} for all six fractions, which were analyzed by ESI- and GALDI-FT-ICR-MS. The observed intensity is presented logarithmic and color-coded (blue: low intensity, yellow: medium intensity, red: high intensity).

S13. Van Krevelen plots of the ESI- and GALDI-FT-ICR-MS data sets for all six LBL fractions

The van Krevelen plots for all six fractions, which were analyzed by ESI(−)- and GALDI(−)-FT-ICR-MS, are illustrated in Figure S20. For this visualization, an O/C ratio as well as a H/C ratio is calculated for each assigned molecular formula. Hence, each molecule is presented in this plot as a single point. Specific O/C and H/C regions can then be correlated to specific compound classes (lipids, sugars, peptides, lignins etc.) to reveal the composition of a complex biomass sample. Lignins are usually found between $O/C = 0.3$ - 0.8 and $H/C = 0.5$ - 1.0. Condensed hydrocarbons occupy a similar H/C region, but a lower O/C region (0 - 0.3). Also, lipids ($O/C = 0$ - 0.3, $H/C = 1.6$ - 2.3), proteins ($O/C = 0.3$ - 0.5, $H/C = 1.5$ - 2.1), amino sugars ($O/C = 0.5$ - 0.8, $H/C = 1.5$ - 2.1) and carbohydrates ($O/C = 0.8$ - 1.3, $H/C = 1.4$ - 2.4) can be distinguished from one another with the help of these van Krevelen plots.[68]

In general, the amount of data points is decreasing for the ESI-MS data sets with increasing fraction number. This illustrates the decreasing solubility and thus polarity of the molecules, which are contained in fractions F5 - F7. Nonetheless, beside lignin compounds, also condensed hydrocarbons, as well as lipids, protein structures, amino sugars and carbohydrates can be supposed according to the van Krevelen plots of all samples. Most of these non-lignin compounds are found in sample F1, which can be attributed to a separation of these potential impurities from the rest of the LBL sample by the conducted fractionation with the water-acetone solvent mixture. Most molecules, which are supposably condensed hydrocarbons, are assigned to the ESI-MS data set of fraction F4, whereas all other compound classes (lignins, lipids, proteins, amino sugars and carbohydrates) are mainly assigned to the ESI-FT-ICR-MS data sets of fractions F1 and F3.

The van Krevelen plots of the GALDI-FT-ICR-MS data sets suggest the presence of the same compound classes, which were already assigned to the ESI-MS data sets. Interestingly, a decrease of the potential lipid, protein, amino sugar and carbohydrate impurities is not observable for the GALDI-MS data sets with increasing fraction number (i.e., amount of acetone in the solvent mixture). Thus, we can conclude that the size of these impurity molecules also increases with the molecular size of the lignin oligomers in each fraction, which leads to a detection of these compounds throughout the entire sample set. This means that GALDI-MS is not only suitable to characterize larger lignin compounds, but also larger molecules from other compound classes.

If the lignin compounds are compared, which are observable by the ESI(−)- and GALDI(−)-FT-ICR-MS analyses, it becomes clear that lignin molecules with slightly higher O/C values are found for ESI (max. $O/C \approx 0.6$) than for GALDI (max. $O/C \approx 0.5$) in the van Krevelen plots. Hence, more polar lignin compounds that possess a higher number of oxygen-containing functionalities (methoxy, carboxy, hydroxy, sulfate, sulfonate etc.) are detectable in the six fractions by utilizing ESI-FT-ICR-MS. In comparison, GALDI-FT-ICR-MS seems to be more suitable for a characterization of less polar lignin molecules, which have a lower number of oxygen-containing functionalities or linkages (e.g., C-O-C bonds).

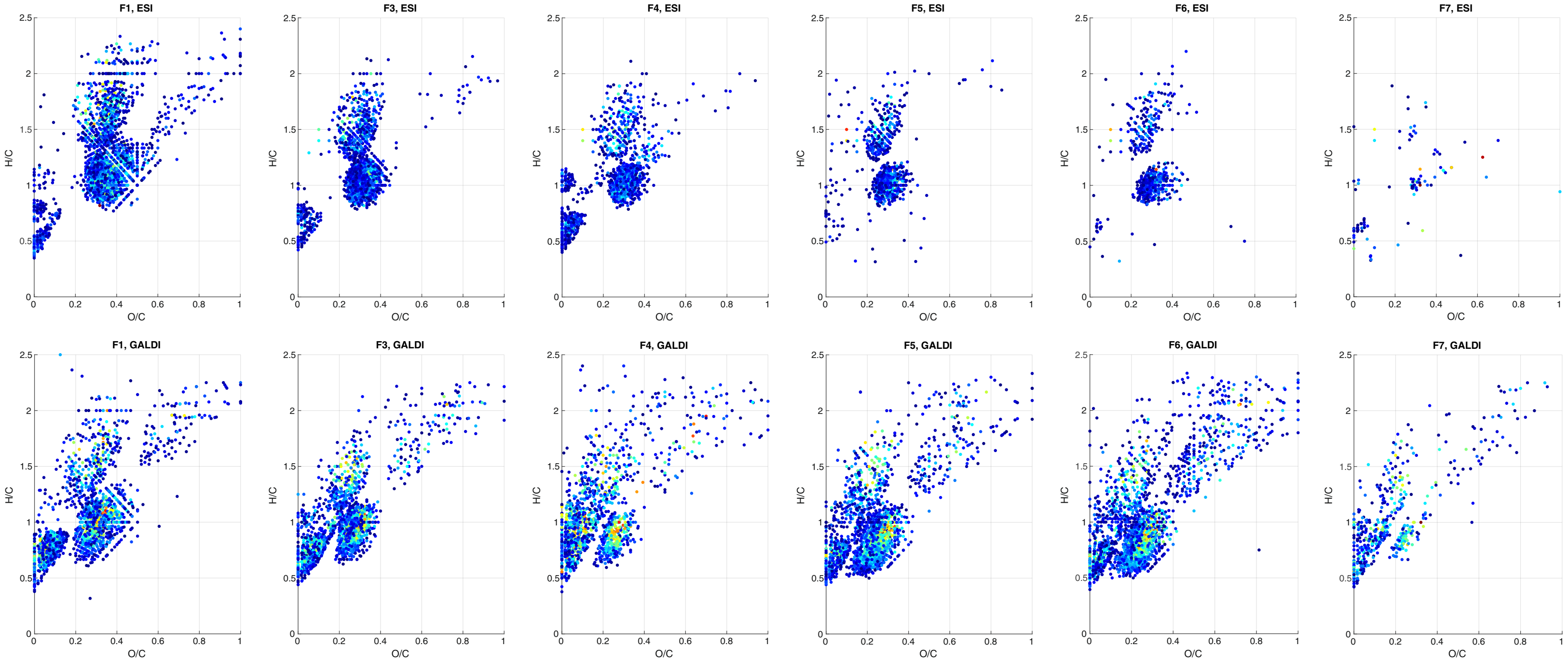

Figure S20. Van Krevelen plots for all six fractions, which were analyzed by ESI- and GALDI-FT-ICR-MS. The observed intensity is presented logarithmic and color-coded (blue: low intensity, yellow: medium intensity, red: high intensity).

S14. Structural suggestions for exemplary lignin compounds

Figure S21 presents structural suggestions for four selected lignin compounds that were structurally characterized by means of ESI-MS/MS and GALDI-MS/MS. These suggestions are based on the detected fragment ions, which are indicative for specific structural sub-units and functional groups.[29,35,47]

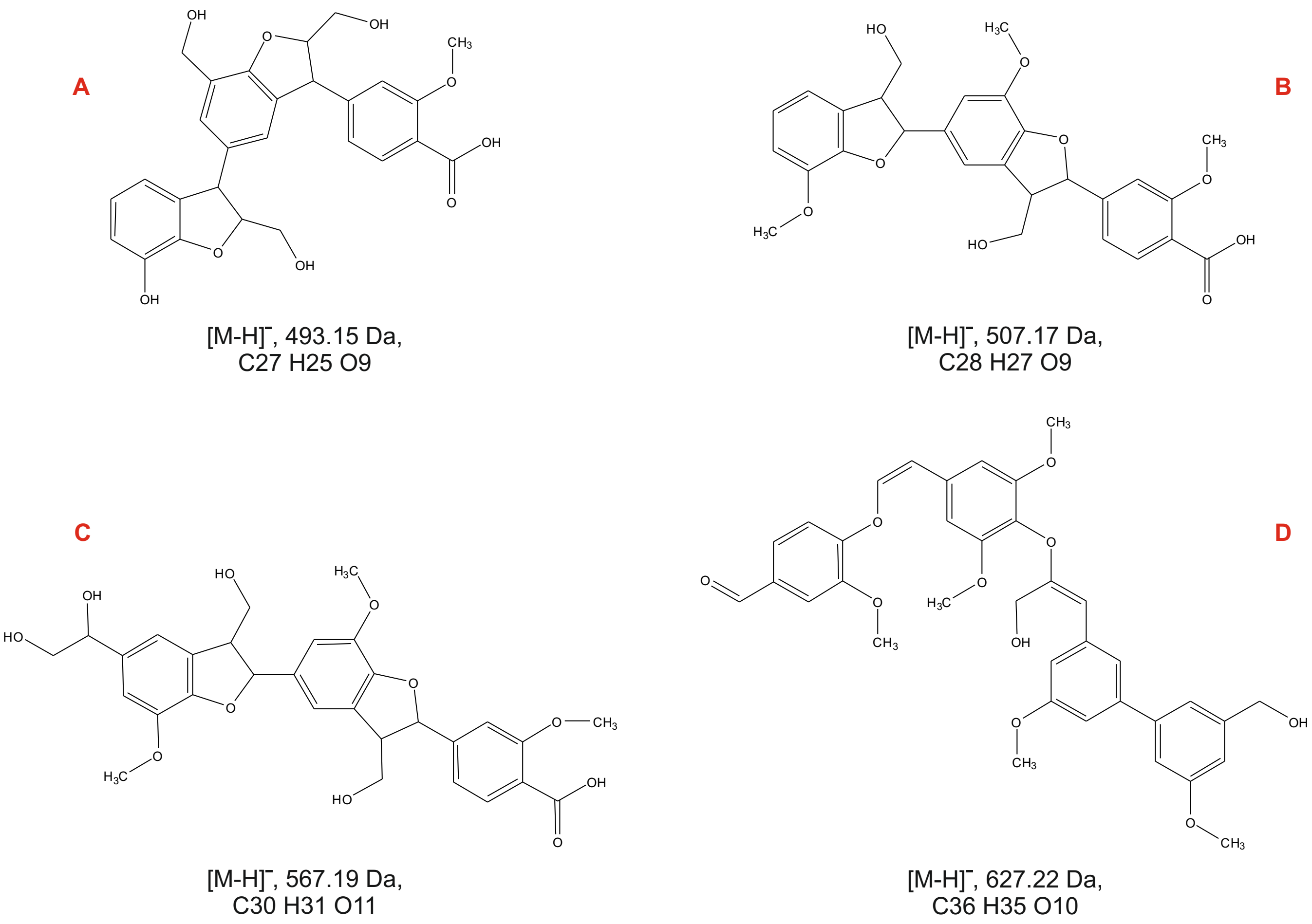

Figure S21. Structural suggestions for four different lignin compounds, which were characterized by ESI- and GALDI-MS/MS. A - 493.15 Da ($C_{27}H_{25}O_9$, $[M\text{-}H]^-$), B - 507.17 Da ($C_{28}H_{27}O_9$, $[M\text{-}H]^-$), C - 567.19 Da ($C_{30}H_{31}O_{11}$, $[M\text{-}H]^-$), D - 627.22 Da ($C_{36}H_{35}O_{10}$, $[M\text{-}H]^-$).

According to the molecular formulae of these four presented ions, we already knew that these compounds are most likely lignin trimers and/or tetramers. The absence of a fragment ion that can be assigned to a combined H_2O and CH_2O loss in all MS/MS spectra can be contributed to basic lignin structures, which consist of G units and are cross-linked by β-5' bonds. Detectable $[M\text{-}H\text{-}CH_3^{\bullet}]^-$ and $[M\text{-}H\text{-}2CH_3^{\bullet}]^-$ fragment ions speak for a presence of multiple methoxy groups in all molecules. $[M\text{-}H\text{-}OH]^-$, $[M\text{-}H\text{-}H_2O]^-$ and $[M\text{-}H\text{-}CH_2O]^-$ ions are also found for all four precursor ions, which indicate to the presence of primary hydroxy groups. Terminal carboxy groups are also expectable for molecules A - C, according to the observed $[M\text{-}H\text{-}CO_2]^-$ and $[M\text{-}H\text{-}HCOOH]^-$ ions in the MS/MS spectra. Interestingly, none of these two fragment ions were detected for molecule D. Instead, a CO loss was observed for this molecule, which is typical for a terminal carbonyl group that is located at cyclic and highly unsaturated structures (e.g., aromatic rings).[29,35]

S15. Further chemometric evaluation of tandem MS analyses of all six LBL fractions

Beside the chemometric evaluation of the tandem MS analyses of precursor ion 507.17 Da ($C_{28}H_{27}O_9$, [M-H]$^-$) by correlation analysis, also hierarchical cluster analysis was applied to compare the different MS/MS analyses of this ion. The results of this chemometric approach are presented hereinafter in Figure S22 as dendrogram.

The dendrogram presents comparable results as the correlation heatmap, which is illustrated in Figure 7 in the main part of this paper. We can observe a clustering of the ESI-MS/MS data, as well as of the GALDI-MS/MS data, with each other. Interestingly, lower distances are found for the GALDI-MS/MS data in comparison to the ESI-MS/MS data. Especially the tandem MS results from fractions F5 - F7, which were analyzed by ESI-MS/MS, have a low distance, which speaks for a high structural similarity of molecules with a molecular formula of $C_{28}H_{28}O_9$ in these samples. Low distances are also observable for F1 and F7 (GALDI) and for F4 and F6 (GALDI). These data sets already showed high correlations with each other. Thus, the low distance values also can be attributed to a high structural similarity of molecules with a molecular formula of $C_{28}H_{28}O_9$ that are contained in these samples.

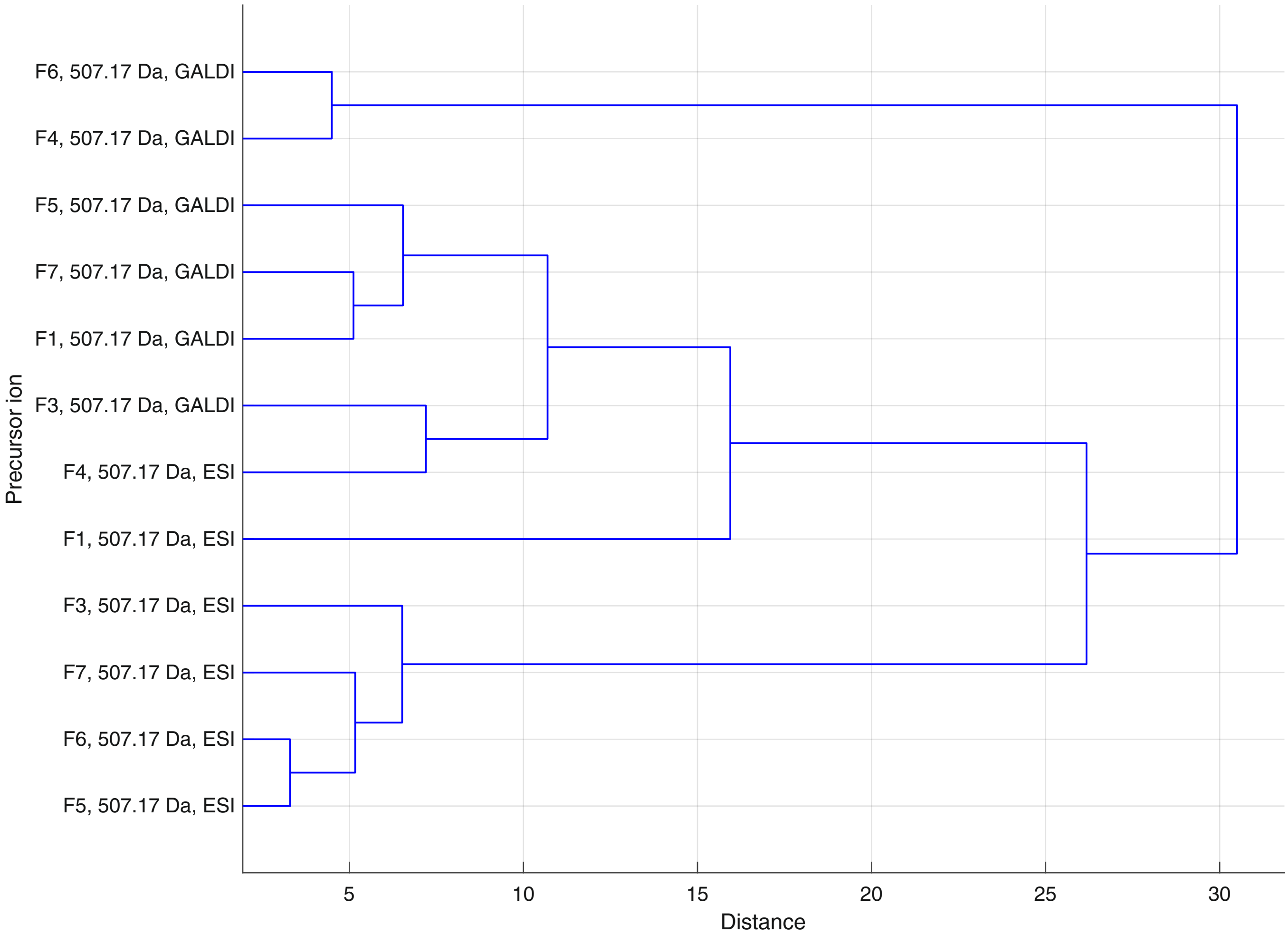

Figure S22. Dendrogram of the hierarchical cluster analysis of the ESI- and GALDI-MS/MS data sets of the six samples for precursor ion 507.17 Da ($C_{28}H_{27}O_9$, [M-H]$^-$), using the relative intensity information of selected fragment ions. For the cluster analysis, Euclidean distance metric and Ward linkage for distance computing were applied.

In Table S8, a detailed comparison of the MS/MS results for precursor ion 507.17 Da in two different fractions and by two different ionization methods is presented (F3 - ESI and F5 - GALDI).

Table S8. Comparison of observable fragment ions for precursor ion 507.17 Da ($C_{28}H_{27}O_9$, $[M\text{-}H]^-$) in data sets of samples F3 - ESI and F5 - GALDI.

**Fragment ion**	Δ m/z [Da]	**F3 - ESI** Intensity [Counts]	**F5 - GALDI** Intensity [Counts]
$[M\text{-}H\text{-}CH_3^{\cdot}]^-$	15.023475	6.95E+06	2.10E+06
$[M\text{-}H\text{-}CH_4]^-$	16.031300	4.79E+06	2.33E+06
$[M\text{-}H\text{-}H_2O]^-$	18.010564	1.34E+06	2.89E+06
$[M\text{-}H\text{-}CO]^-$	27.994914	–	1.44E+06
$[M\text{-}H\text{-}CHO]^-$	29.002739	–	–
$[M\text{-}H\text{-}CH_2O]^-$	30.010564	–	2.59E+06
$[M\text{-}H\text{-}CH_4O]^-$	32.026214	2.31E+06	2.34E+06
$[M\text{-}H\text{-}CH_2CO]^-$	42.010564	–	–
$[M\text{-}H\text{-}CO_2]^-$	43.989829	1.29E+06	–
$[M\text{-}H\text{-}CH_2O\text{-}CH_3^{\cdot}]^-$	45.034039	–	–
$[M\text{-}H\text{-}HCOOH]^-$	46.005479	–	1.79E+06
$[M\text{-}H\text{-}H_2O\text{-}CH_2O]^-$	48.021129	–	1.43E+06
$[M\text{-}H\text{-}2CO]^-$	55.989829	–	–
$[M\text{-}H\text{-}CH_2O\text{-}CO]^-$	58.005479	–	1.25E+06
$[M\text{-}H\text{-}2CH_2O]^-$	60.021129	–	1.41E+06
$[M\text{-}H\text{-}H_2O\text{-}CO_2]^-$	62.000394	1.30E+06	–
$[M\text{-}H\text{-}2CO_2]^-$	87.979658	1.25E+06	–

In general, the MS/MS results of both ions showed no significant correlation ($\rho = 0.38$). If we take a detailed look at the detectable fragment ions and their intensities in Table S8, we can understand why the correlation coefficient was relatively low. Only four of 17 potential fragment ions were detected in both MS/MS analyses ($[M\text{-}H\text{-}CH_3]^-$, $[M\text{-}H\text{-}CH_4]^-$, $[M\text{-}H\text{-}H_2O]^-$ and $[M\text{-}H\text{-}CH_4O]^-$). Four other fragment ions were not detected in either of the two analyses ($[M\text{-}H\text{-}CHO]^-$, $[M\text{-}H\text{-}CH_2CO]^-$ and $[M\text{-}H\text{-}CH_2O\text{-}CH_3]^-$). Thus, the other nine fragment ions and the ion intensities, which were observed for these ions, are the main reason for the relatively low correlation coefficient.

The commonly detected ($[M\text{-}H\text{-}CH_3]^-$, $[M\text{-}H\text{-}CH_4]^-$, $[M\text{-}H\text{-}H_2O]^-$ and $[M\text{-}H\text{-}CH_4O]^-$ fragment ions can be assigned to lignin structures, which possess methoxy and primary hydroxy groups. Furthermore, the absence of the $[M\text{-}H\text{-}H_2O\text{-}CH_2O]^-$ ion in the ESI-MS/MS spectrum of F3 speaks for β-5' bonds between the lignin sub-units. During the GALDI-MS/MS analysis of F5, $[M\text{-}H\text{-}HCOOH]^-$ and $[M\text{-}H\text{-}H_2O\text{-}CH_2O]^-$ fragment ions were observed, which are typical for resinol structures with β-β' linkages. No CO_2 loss was detected for F5 - GALDI, whereas two CO_2 losses are observed for F3 - ESI. Hence, a minimum number of two carboxy groups is assumable for the lignin precursor ion in F3 - ESI, whereas the molecule in F5 - GALDI seems to possess no terminal carboxy groups.[29,35]

Beside the chemometric evaluation of precursor ion 507.17 Da ($C_{28}H_{27}O_9$, $[M\text{-}H]^-$), also the results for the correlation analysis of the tandem MS data sets for all characterized fragment ions are presented in Figure S23. In this plot, high correlation coefficients can be attributed to a high number of structural similarities between two ions. For instance, the correlation coefficient between the MS/MS data for F5 - 327.09 Da - GALDI and F7 - 591.17 Da - GALDI is relatively high with $\rho = 0.73$. Hence, these two molecules in the two samples share comparable lignin sub-units and functional groups. Their greatest difference is, most likely, the oligomeric size. The generally low correlation coefficients for precursor ions F1 - 581.18 Da - ESI; F4 - 627.22 Da - ESI; F4 - 821.28 Da - ESI and F4 - 301.22 Da - GALDI with all other precursor ions are attributable to a generally low number of detectable, lignin-specific, fragment ions. For instance, only a $[M\text{-}H\text{-}2CH_2O]^-$ fragment ion and no other lignin-typical fragment ion is observable for the precursor ion 301.22 Da of sample F4 - GALDI.

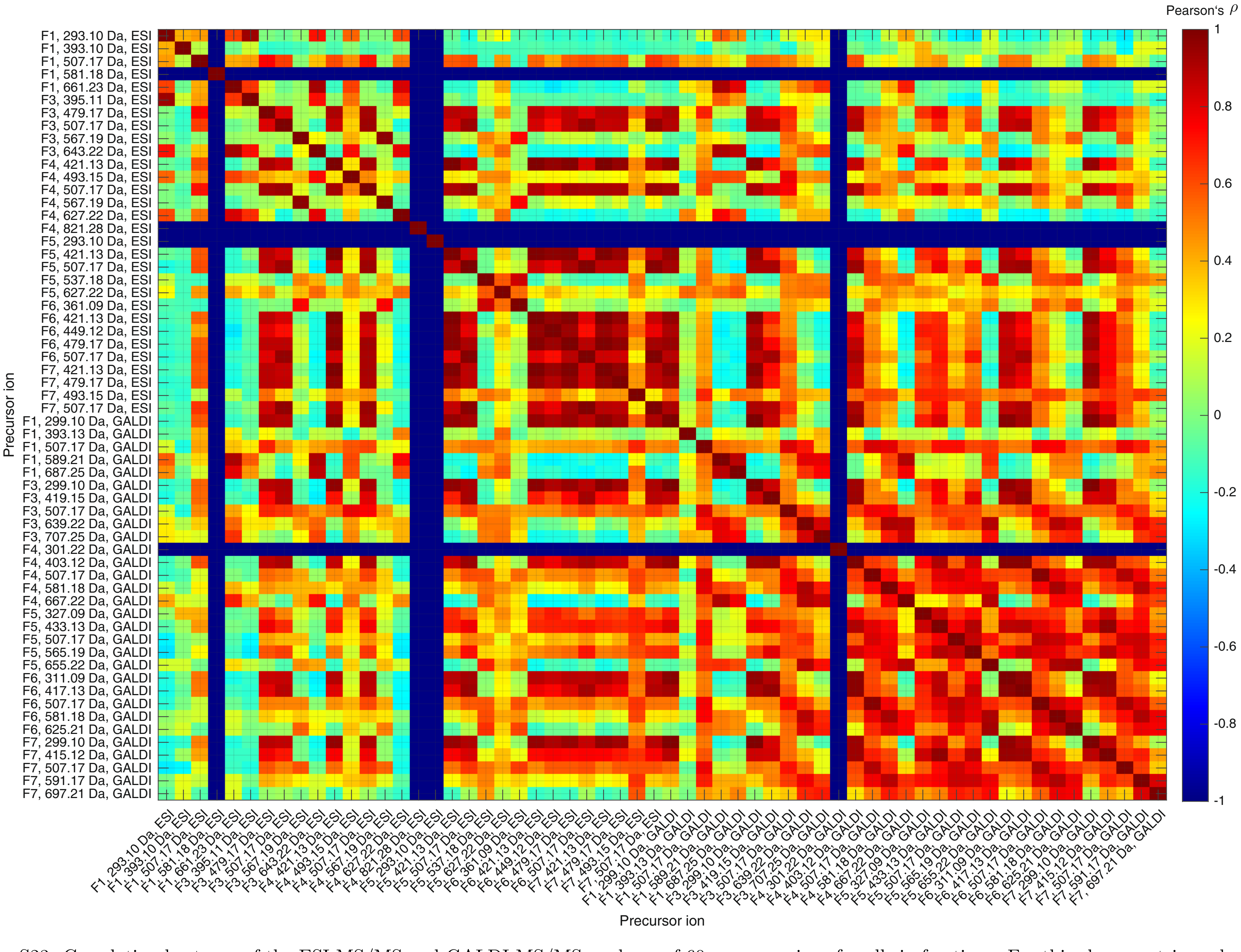

Figure S23. Correlation heatmap of the ESI-MS/MS and GALDI-MS/MS analyses of 60 precursor ions for all six fractions. For this chemometric evaluation the relative intensity information and a confidence interval of 99.9 % was used.

S16. Additional hetero spectroscopic correlation results

Beside the hetero spectroscopic correlation results, which are presented in the main part of the paper, also further correlation analyses were conducted to combine the spectral information of FT-ICR-MS, tandem MS and NMR data sets.

Correlation heatmaps, as a result of this data combination process for quantitative ^{13}C-NMR and GALDI-MS/MS data sets, ^{1}H-NMR and ESI-MS/MS data sets as well as ^{1}H-NMR and GALDI-MS/MS data sets are presented in Figure S24 - S26. Furthermore, a contour plot as result of the hetero spectral combination of ^{1}H-NMR and GALDI-MS data sets is illustrated in Figure S27.

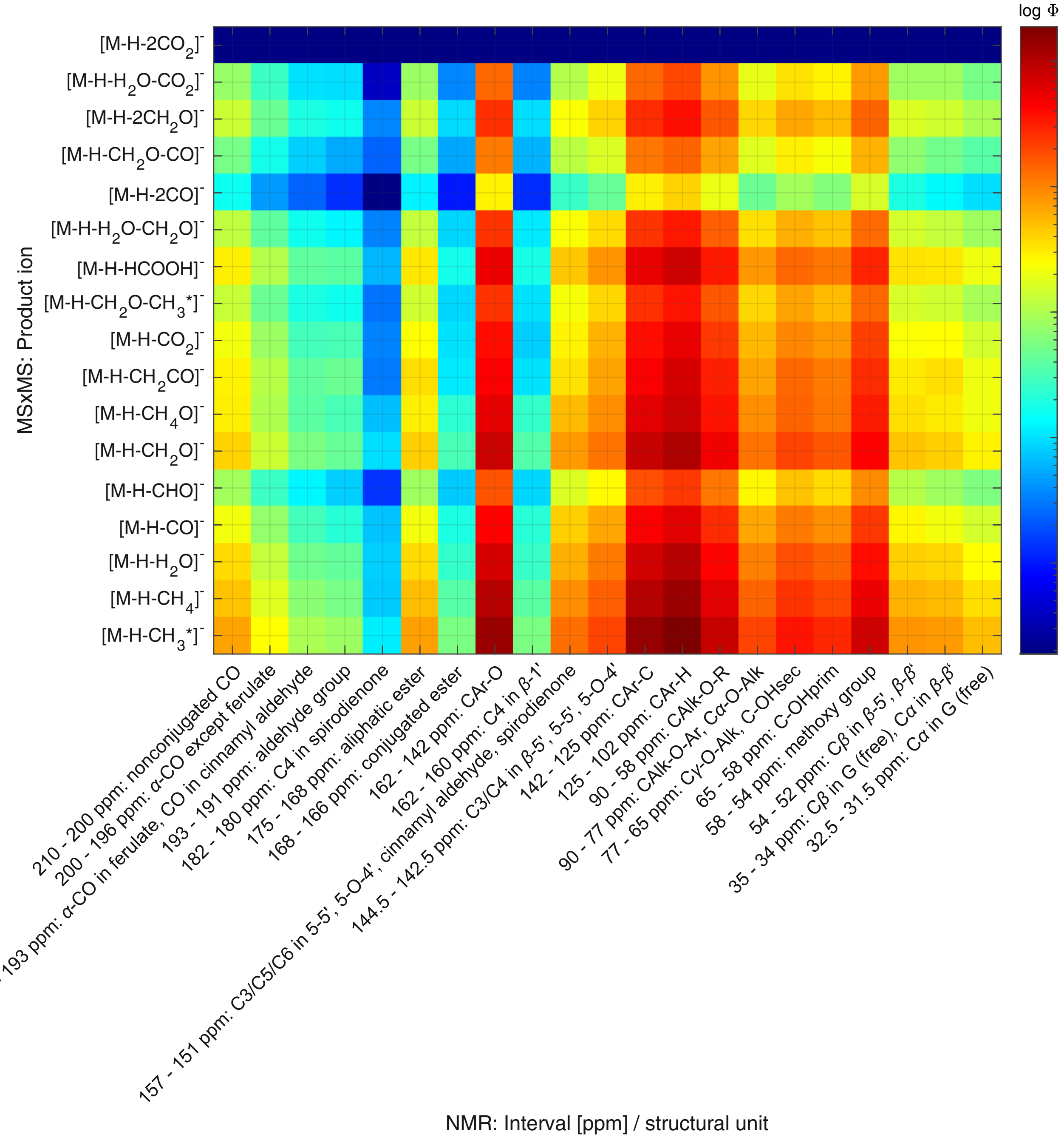

Figure S24. Correlation heatmap as result of the hetero spectral combination of quantitative ^{13}C-NMR and GALDI-MS/MS data sets. The calculated correlation coefficients Φ are presented logarithmic and color-coded.

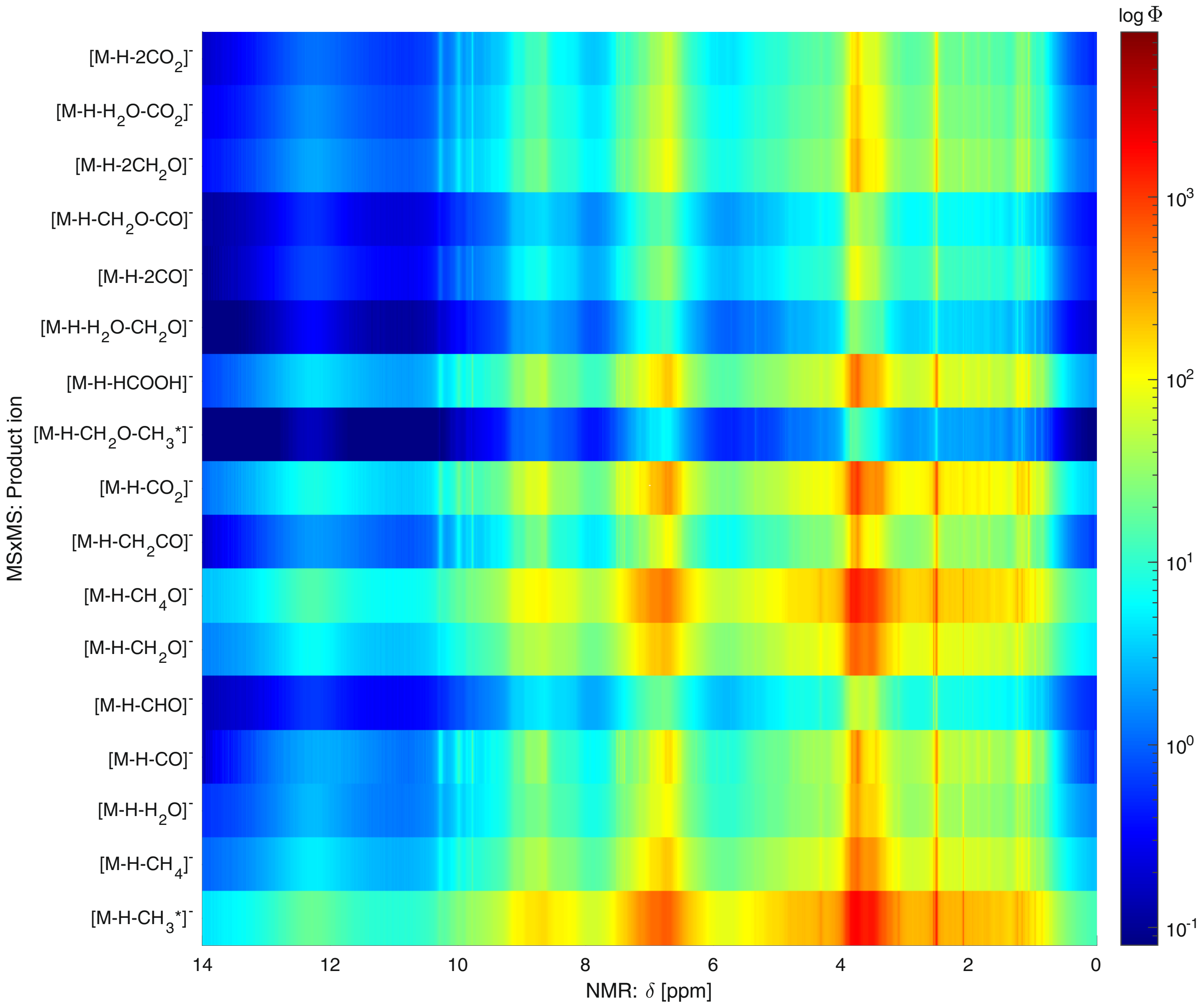

Figure S25. Correlation heatmap as result of the hetero spectral combination of ^{1}H-NMR and ESI-MS/MS data sets. The calculated correlation coefficients Φ are presented logarithmic and color-coded.

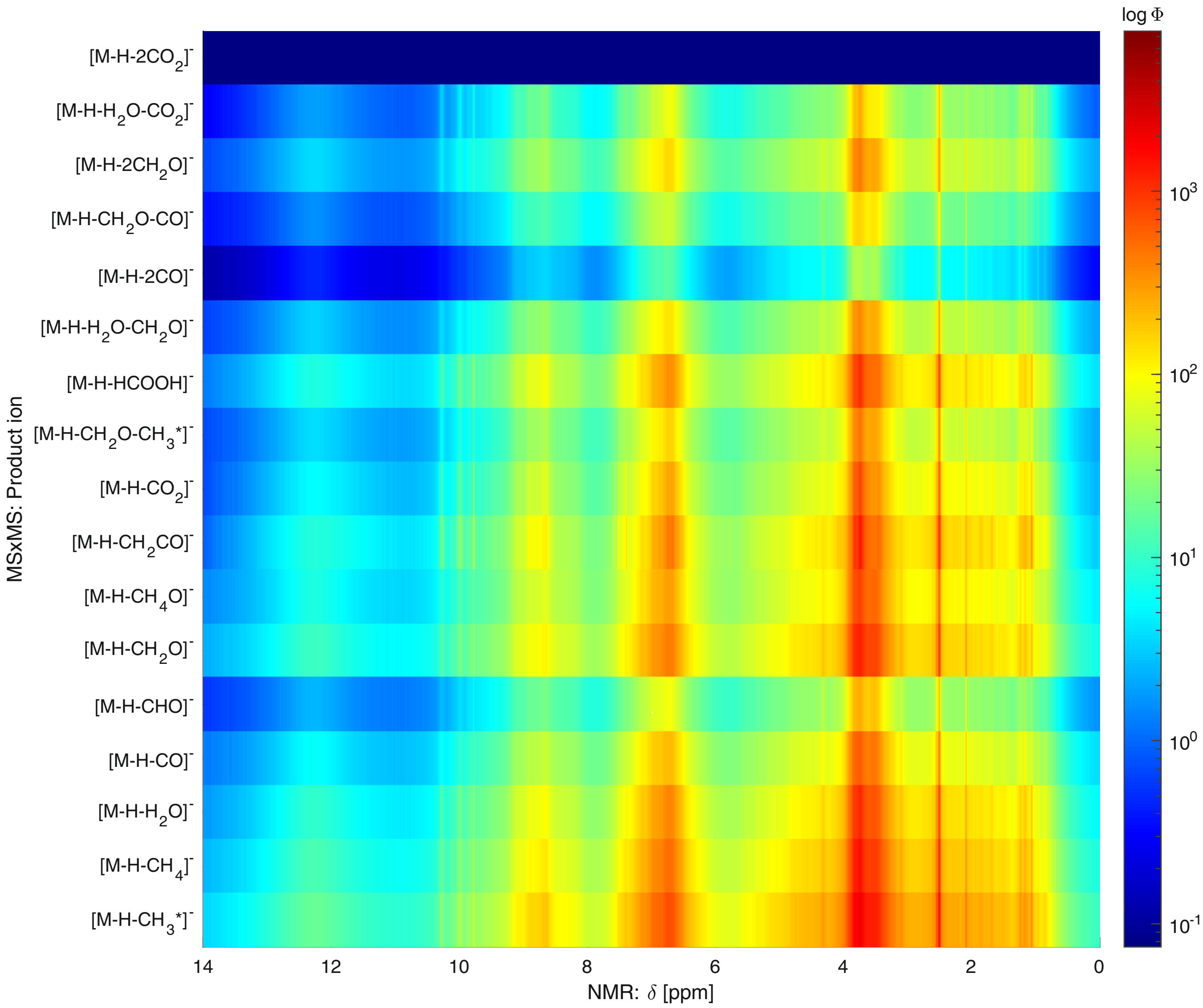

Figure S26. Correlation heatmap as result of the hetero spectral combination of ^{1}H-NMR and GALDI-MS/MS data sets. The calculated correlation coefficients Φ are presented logarithmic and color-coded.

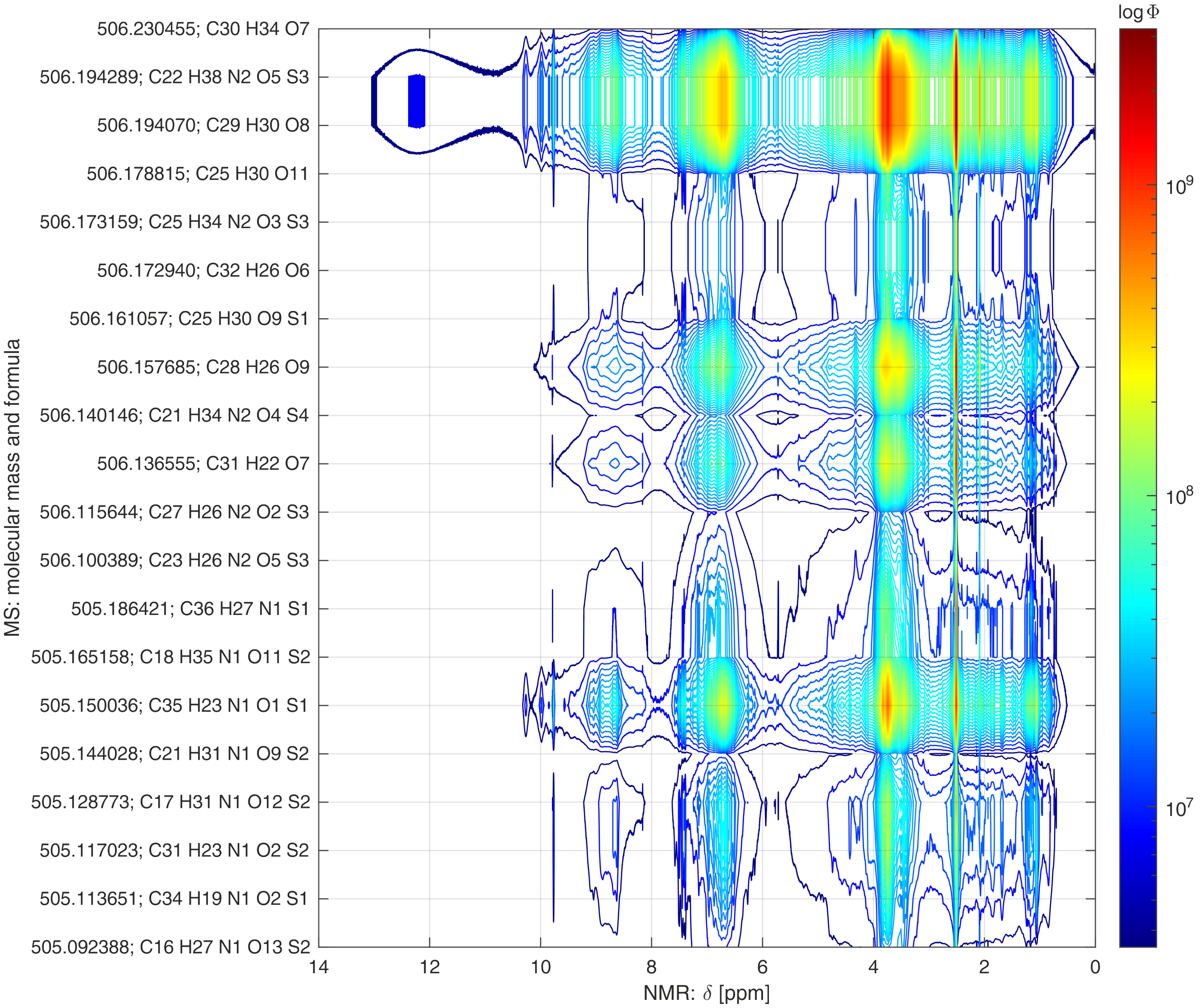

Figure S27. Contour plot as result of the hetero spectral combination of ^{1}H-NMR and GALDI-MS data sets in a mass range from 505 to 506 Da. m/z values and ion formulae were proton and electron corrected to obtain molecular masses and formulae. The calculated correlation coefficients Φ are presented logarithmic and color-coded.
